# Family Game Show-style Didactic for Teaching Nervous System Disorders during Emergency Medicine Training

**DOI:** 10.21980/J8D357

**Published:** 2020-04-15

**Authors:** Alaina Brinley Rajagopal, Gabriel Sudario, David Weiland, Megan Boysen Osborn

**Affiliations:** * University of California, Irvine, Department of Emergency Medicine, Orange, CA

## Abstract

**Audience:**

Emergency medicine residents and medical students

**Introduction:**

The field of emergency medicine requires learners to build a vast library of illness scripts to be accessible in a rapid manner. Illness scripts are refined and reinforced as senior physicians teach learners common associations between diagnoses, presentation, workup findings, and treatment modalities.^1^ In order to examine these associations, we developed a didactic session based on the popular television game show “Family Feud” to teach important neurologic conditions related to emergency medicine. This lecture was designed to be an interactive competition, leveraging group participation, competition, and expert opinion.

Neurologic emergencies are very common, affecting millions of Americans yearly. It is important for emergency medicine physicians to quickly recognize these conditions and initiate treatment because delay can lead to devastating outcomes.^2^ The neurologic conditions covered in the lecture were chosen based on the 2016 EM model of clinical practice, sections 7.0: Head, ear, eye, nose, throat disorders, 10.0: Systemic infectious disorders, 12.0: Nervous system disorders, and 19.0: Procedures and skills integral to the practice of emergency medicine, as well as author experience.

**Educational Objectives:**

By the end of this didactic exercise the learner will: 1) name 13 important neurologic conditions related to emergency medicine: TPA (tissue plasminogen activator) contraindications/TPA eligibility, optic neuritis, botulism, giant cell (temporal) arteritis, viral encephalitis, neurocysticercosis, rabies, myasthenia gravis, neurosyphilis, status epilepticus, Bell’s palsy, dementia vs. delirium, acute inflammatory demyelinating polyneuropathy (Guillain-Barré); 2) recognize five pattern words associated with each neurologic condition; 3) understand exam findings, diagnostic tests, and/or treatments for 13 important neurologic conditions.

**Educational Methods:**

A survey was sent through a national emergency medicine education listserv (Council of Residency Directors in Emergency Medicine [CORD-EM]) asking educators to list common word or phrase associations that come to mind with a list of neurological diagnoses. A PowerPoint lecture was created in the form of the game, Family Feud, using the data from this national survey. The game Family Feud requires participant teams to guess answers to certain questions by attempting to guess the most popular answers of survey respondents. At our weekly residency conference, residents were divided into teams and offered the opportunity to compete in a game testing knowledge of nervous system disorders. Each neurology topic was then addressed by a mini-lecture to review pertinent concepts in the disease process. There was no formal assessment at the end of this lecture; however, learners actively participated throughout the lecture. Questions were discussed at the end of each round giving learners the opportunity to fully understand topics.

**Research Methods:**

Efficacy of the educational content was assessed based on learner feedback as well as observation of the learners during the exercise.

**Results:**

Learners were engaged with the exercise and verbal feedback was uniformly positive. Learners were enthusiastic about the format and requested more sessions created in a similar game.

**Discussion:**

Based on feedback as well as observation of the learners, the lecture was both an effective highyield neurology refresher and team-building exercise. Learners enjoyed the opportunity to compete as a team. Gamification seemed to improve student enjoyment, engagement, and attention, which has also been shown in the literature.^3^ Our residency program intends to implement similar lectures in the future.

**Topics:**

Neurology, TPA contraindications, TPA eligibility, upper motor neuron lesion, lower motor neuron lesion, optic neuritis, aphasia, botulism, ACA (anterior cerebral artery) stroke, giant cell (temporal) arteritis, Bell’s palsy, viral encephalitis, Todd’s paralysis, neurocysticercosis, tonic-clonic seizure, rabies, epidural hematoma, myasthenia gravis, spinal cord injury, neurosyphilis, Glasgow Coma Score (GCS), status epilepticus, Horner’s syndrome, subarachnoid hemorrhage, dementia, delirium, Parkinson’s disease, acute inflammatory demyelinating polyneuropathy (Guillain-Barré).

## USER GUIDE

[Table t2-jetem-5-2-l1]

**Table t2-jetem-5-2-l1:** 

List of Resources:	
Abstract	1
User Guide	2
Appendix A: Pre-round Questions	8
Appendix B: Round Topics with Explanation/Important Points for Learners	13
Appendix C: Family Feud – Neurology PowerPoint	19

**Learner Audience:**

Medical students, Interns, Junior Residents, Senior Residents

**Time Required for Implementation:** Instructors may use 13 or fewer rounds, depending on the time available. We recommend 1.5–2 hours for all 13 rounds. A one-hour length can be easily accommodated, but some rounds would be eliminated. We implemented the session during a 90-minute block during residency conference.

**Recommended Number of Learners per Instructor:**

We divided learners into four groups or “families.” For maximal participation, the recommended number of learners should be 1–6 per group; however, anyone watching the lecture/game will be able to learn the content by attentiveness to the questions and explanations.

**Topics:**

Neurology, TPA contraindications, TPA eligibility, upper motor neuron lesion, lower motor neuron lesion, optic neuritis, aphasia, botulism, ACA (anterior cerebral artery) stroke, giant cell (temporal) arteritis, Bell’s palsy, viral encephalitis, Todd’s paralysis, neurocysticercosis, tonic-clonic seizure, rabies, epidural hematoma, myasthenia gravis, spinal cord injury, neurosyphilis, Glasgow Coma Score (GCS), status epilepticus, Horner’s syndrome, subarachnoid hemorrhage, dementia, delirium, Parkinson’s disease, acute inflammatory demyelinating polyneuropathy (Guillain-Barré).

**Objectives:**

By the end of this didactic exercise the learner will:

1. Name 13 important neurologic conditions related to emergency medicine: TPA contraindications/TPA eligibility, optic neuritis, botulism, giant cell (temporal) arteritis, viral encephalitis, neurocysticercosis, rabies, myasthenia gravis, neurosyphilis, status epilepticus, Bell’s palsy, dementia vs. delirium, acute inflammatory demyelinating polyneuropathy (Guillain-Barré).
2. Recognize five pattern words associated with each neurologic condition.
3. Understand exam findings, diagnostic tests, and/or treatments for 13 important neurologic conditions.

### Linked objectives and methods

Emergency medicine residents are required to have didactic training one time per week. Gamification is an increasing trend in medical education, showing increased engagement and possible benefits in learning outcomes.^3^ This family-style trivia game was designed to improve participation through a competitive game; the trivia design of the game addressed objectives one and two. After each round, the topics were briefly reviewed in mini-lectures before moving to the next topic, which helped learners meet objective three. We covered major points for each topic/disease over a series of 1–3 slides. Students were able to ask questions to clarify any knowledge gaps. The rapid transition between topics as well as the possibility of being called upon encouraged attention.

### Recommended pre-reading for instructor

Review of neurology topics using textbooks, primary journal articles, or web-based resources would be recommended. We recommend refreshing one’s knowledge with the following:

- Portrale JV, Estreicher MB, Lopez BL. Nervous system disorders. In: Schofer JM, ed. *Emergency Medicine: A Focused Review of the Core Curriculum*. 2nd ed. AAEM Resident & Student Association, Inc; 2015:309–340.^4^

### Learner responsible content (LRC, optional)

Learners should review the following prior to playing the game:

- Portrale JV, Estreicher MB, Lopez BL. Nervous System Disorders. In: Schofer JM, ed. *Emergency Medicine: A Focused Review of the Core Curriculum*. 2nd ed. AAEM Resident & Student Association, Inc; 2015:309–340.^4^

### Survey development

In order to identify survey answers for the game, we sent an online survey to Emergency Medicine (EM) board certified and board eligible faculty on the CORD-EM listserv and to faculty at the Department of EM at the University of California, Irvine via email. The survey asked the following question: “What is the first thing that comes to mind when you think of…?” followed by the opportunity to provide free text answers associated with several conditions. We chose conditions based on the 2016 EM model of clinical practice, sections 7.0: Head, ear, eye, nose, throat disorders, 10.0: Systemic infectious disorders, 12.0: Nervous system disorders, and 19.0: Procedures and skills integral to the practice of emergency medicine, as well as author experience. We piloted the survey on our faculty at the University of California, Irvine. Based on the results of that survey, we changed one condition from Multiple Sclerosis to Optic Neuritis.

We included the following conditions in the survey on the CORD listserv: Optic Neuritis, Guillain Barré, NIH Stroke Scale, TPA Contraindications, Botulism, Tick Paralysis, Neurosyphilis, Status Epilepticus, Delirium, Lumbar Puncture, Myasthenia Gravis, Bell’s Palsy, Rabies, Neurocysticercosis, Temporal Arteritis, and Viral Encephalitis. We coded responses “keywords” to each question and grouped common themes together. Of the departmental faculty and fellows surveyed, we received 18 responses (response rate: 67%). We received 77 responses from our query on the CORD-EM listserv. These responses were collected in August 2018. We included survey results from faculty and CORD-EM faculty in our final results (Table I). Based on which conditions had the most straightforward survey responses, the senior author selected the following conditions for inclusion in the game: TPA contraindications, Botulism, Idiopathic facial nerve palsy (Bell’s palsy), Giant-cell arteritis (Temporal arteritis), Viral encephalitis, Neurocysticercosis, Status epilepticus, Rabies, Myasthenia gravis, Neurosyphilis, Delirium, and Acute inflammatory demyelinating polyneuropathy (AIDP/Guillain-Barré Syndrome, and Optic neuritis.

Table I: (see page 7) Keywords associated with each condition as determined by survey results. The numbers adjacent to each keyword reflect the number of survey respondents who offered that association. The numbers do not always add up to the same value because the pilot survey did not include optic neuritis. Also, some respondents skipped some questions during the survey. Some infrequent answers were excluded if they were deemed to not be educationally valuable by the senior author. Abbreviations: FVC, Forced Vital Capacity; NIF, Negative Inspiratory Force, VC, Vital Capacity; INR, international normalized ratio; ESR, erythrocyte sedimentation rate; INH, isoniazid.

### Slide preparation

The host prepares the PowerPoint by preparing slides for the a) pre-round question (insert a PowerPoint slide with the pre-round question into the deck) b) the survey responses c) summary slides. Each round will consist of a pre-round question, an animated survey slide, and a summary slide. We imported survey results, which are included in Table 1, into a game style PowerPoint, which can be found at: http://www.youthdownloads.com/games/family-fuedpowerpoint.^5^ The following YouTube (https://www.youtube.com/watch?v=Lo4VkGHzt3k) video provides an overview on how to import answers into slides.^6^ Exemplar slides are available in the supplemental files of this publication.

### Game play

It is recommended that the pre-round question be used to determine which team will play the round (see Appendix 1). The educational host reads the pre-round question. Teams can buzz in to try to answer the pre-round question; teams are disqualified from the pre-round question if they buzz in before the entire question is read. The winner of the pre-round question becomes the team that will have the first opportunity to guess the keywords for that round. The host reads the survey question as: “What comes to mind when you think of [condition].” Teams earn points for every keyword answered correctly. For example, if a team guesses “honey” for the botulism question, they would earn 38 points, corresponding to how many survey respondents gave that answer. Teams can either discuss answers as a group or answer one at a time, depending on the learner comfort and level. If the team was not able to guess all keywords prior to having three incorrect answers (“strikes”), the remaining teams will have the opportunity to buzz in to guess any remaining keywords. The first team to buzz in with a correct keyword earns the points from that keyword and the opportunity to guess any remaining keywords. Play is continued in this fashion until all keywords have been guessed, allowing each team up to three strikes per round until all keywords have been guessed. In our experience, no more than two teams usually participate in each round. After all keywords are guessed, the host provides teaching pearls about the condition. Teaching pearls for each condition are included in Appendix 2.

### Results and tips for successful implementation

This exercise was presented during residency conference for a group of approximately 24 emergency medicine residents and medical students. We did not obtain a direct assessment of learner acquisition of knowledge. Efficacy of the educational content was assessed based on learner feedback as well as observation of the learners during the exercise. We observed high learner engagement and enjoyment. No modifications were needed after initial implementation, although we found it is best implemented in a group setting with between one and six learners per group and two to six groups.

### Associated content (optional)

Please find the attached Appendices 1  and 2 for pre-round questions and teaching pearls from each round.

### Technology necessary

A computer and projector are required to present the questions in PowerPoint format. Audience response buzzers are required to determine which team will play for points during the next round. There are a multitude of audience response systems available. We used “Eggspert” buzzers; however, one could use any audience response system that can allow a team to buzz in first.^7^ We used a premade family trivia-style PowerPoint, which can be found at http://www.youthdownloads.com/games/family-fuedpowerpoint.^5^ If using the PowerPoint templates and/or buzzers is too resource intensive, one could easily play the game using a dry erase board to record answers and points and asking questions verbally. One could also have students raise their hands instead of using an audience response system. The PowerPoint is advantageous because summary slides can also be added; however, these summaries could also be discussed verbally without PowerPoint. Similarly, the pre-round questions could also be read aloud instead of shown on slides.

### Assessment (optional)

We did not formally evaluate learners after this intervention. However, learner understanding was assessed by the interactive nature of the game throughout play. Learners were engaged throughout the entirety of the implementation. After completing the exercise, learners expressed that they found the exercise entertaining as well as valuable for refreshing neurology knowledge.

## Supplementary Information

Please see associated PowerPoint file

## Figures and Tables

**Table 1:** Keywords associated with each condition as determined by survey results. The numbers adjacent to each keyword reflect the number of survey respondents who offered that association. The numbers do not always add up to the same value because the pilot survey did not include optic neuritis. Also, some respondents skipped some questions during the survey. Some infrequent answers were excluded if they were deemed to not be educationally valuable by the senior author.

Condition	Keyword 1 (n)	Keyword 2 (n)	Keyword 3 (n)	Keyword 4 (n)	Keywords 5–8 (n)
Acute inflammatory demyelinating polyneuropathy (AIDP/Guillain-Barré Syndrome)	Ascending weakness (56)	Areflexia (6)	Vaccines (5)	Campylobacter/diarrhea (5)	NIF (2)
Botulism	Honey (38)	Floppy baby (14)	Canned food (10)	Cranial nerve palsy (8)	IV drugs (6)
Delirium	Withdrawal/Alcohol (19)	Urinary tract infection (16)	Waxing/waning (9)	Confusion (5)	Elderly (5)
Giant-cell arteritis (Temporal arteritis)	ESR (33)	Elderly (14)	Jaw claudication (7)	Headache (7)	Biopsy (5) Steroids (5) Temporal tenderness (3)
Bell’s Palsy	Forehead involvement (24)	Facial nerve (19)	Lyme disease/tick (11)	Steroids (5)	Eye drops (2)
Myasthenia gravis	Ptosis/diplopia (27)	Edrophonium/tensilon test (15)	NIF/VC/FVC (15)	Ice pack test (7)	Fatigue/tired/gets worse (4)
Neurocysticercosis	Pork (39)	Latin American (14)	Seizures (10)	CT hyperdensities/calcifications (7)	HIV/Immunodeficiency (4) Tapeworm (3)
Neurosyphilis	Penicillin (15)	Dementia/crazy (10)	Tabes dorsalis/posterior column (8)	Tuskegee (6)	Elderly (3) Missed (3) Lumbar puncture (3) Argyll-Robinson pupil (3)
Optic neuritis	Multiple sclerosis (59)	Red color desaturation (9)	Vision loss (9)	Painful (5)	Steroids (1)
Rabies	Bats (36)	Multiple vaccines (15)	Racoon (10)	Skunk (5)	Uncommon (2)
Status epilepticus	Benzodiazepine (39)	Intubation (8)	Propofol (6)	Phenytoin/Fosphenytoin/20mg/kg (5)	INH overdose/pyridoxine (4) Keppra (4) Phenobarbital (2)
TPA contraindications	Bleeding (47)	Elevated INR/Anticoagulant use (9)	Blood pressure/Hypertension (5)	Time (3)	Age (3)
Viral encephalitis	Herpes (23)	West Nile virus (21)	Confusion/Altered mental status/Psychiatric (9)	Acyclovir (6)	Lumbar puncture (5) Mosquito (4)

Abbreviations: FVC, Forced Vital Capacity; NIF, Negative Inspiratory Force, VC, Vital Capacity; INR, international normalized ratio; ESR, erythrocyte sedimentation rate; INH, isoniazid

**Figure 1:**
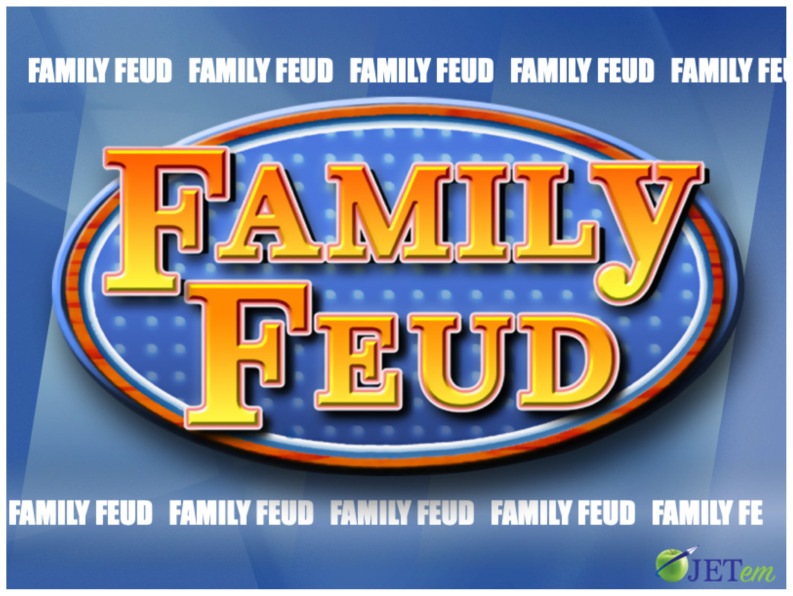
Please see associated PowerPoint File

## Appendix A Pre-round Questions

The following questions were used to determine which team would play for the next round. For example, the pre-round question for round one was asked at the beginning of round one. The teams used an audience response system to buzz in sequentially after the question was read by the host. The team who activated their audience response buzzer first would be able to answer the pre-round question first. If the pre-round question was answered correctly, that team would have the opportunity to guess the round. If the team answered incorrectly, the remaining teams were given the opportunity to buzz in to answer the question. If the second team answered correctly, they would then get to guess the round. If they were incorrect, the remaining teams would then have the opportunity to buzz in until the question was answered correctly and the team who would guess the round was determined.

### Pre-round question 1

Question: A patient comes in with right sided hemiparesis. What is the ONE lab you need to check first?

Answer: Glucose

Explanation: Hypoglycemia is known to cause stroke-like symptoms. It is even possible to see abnormal findings in imaging studies in the setting of hypoglycemia. Hypoglycemia is usually an easily correctable condition. Once glucose is corrected, the hemiparesis often resolves. Therefore, it is critical for learners to recognize that evaluating blood glucose levels, particularly before giving therapeutic agents such as tissue plasminogen activator (TPA), is a critical action in patients presenting with neurologic deficits.^8^

### Pre-round question 2

Question: Does the following describe an upper or lower motor neuron lesion?

-Spasticity present
-No wasting
-Hyperactive deep tendon reflexes
-Positive Babinski’s sign
-No fasciculations

Answer: Upper motor neuron lesion

Explanation: Motor neuron disease is comprised of many neurodegenerative disorders which are characterized by both upper and lower motor neuron lesions. Upper motor neuron symptoms include hypertonia, hyperreflexia, Babinski’s sign, spasticity, slow speech, and Hoffman’s sign while lower motor neuron symptoms include weakness, muscle atrophy, fasciculations, dysarthria, tongue atrophy, and reduced reflexes. Some motor neuron diseases include Amyotrophic lateral sclerosis (ALS), progressive muscular atrophy, pseudobulbar palsy, spinal muscular atrophy, hereditary spastic paraplegia, and primary lateral sclerosis with ALS being most common.^9^–^11^

### Pre-round question 3

Question: What type of aphasia is this?

-Non-fluent speech
-Poor repetition and naming
-Good auditory comprehension

Answer: Broca’s (expressive) aphasia

Explanation: Broca’s aphasia is an expressive aphasia meaning the patient has good comprehension but is unable to express his responses verbally or in written form. Patients may experience difficulty with word finding, hesitancy, non-fluency, repetition, or appear confused with relatively spared auditory comprehension. These symptoms are usually associated with infarction (middle cerebral artery), infection, inflammation, or other damage to Broca’s area of the brain (motor center in the left posterior inferior frontal cortex and insula).^12^,^13^

### Pre-round question 4

Question: Where is the stroke?

-The patient has isolated leg paresis

Answer: Anterior cerebral artery (ACA)

Explanation: The homunculus describes anatomical connectivity between limb and brain. It was developed in 1937 and since has undergone some revision. It still roughly correlates brain lesions to anatomic structures. The ACA is associated with lesions such as lower and upper extremity weakness, apraxia, aphasia, and language dysfunction. Lower extremity weakness is usually worse than upper extremity weakness.^14^–^16^

### Pre-round question 5

Question: A 32-year-old female in 3rd trimester of pregnancy presents with sudden onset hemifacial weakness, diminished taste, hyperacusis, and difficulty closing one eye. Diagnosis?

Answer: Idiopathic facial nerve palsy (Bell’s palsy).

Explanation: Pregnancy is a hypercoagulable state, and thus pregnant patients are at increased risk for stroke. It is important to be able to distinguish the features of stroke with more benign conditions such as idiopathic facial nerve palsy. Idiopathic facial nerve palsy has an incidence of 24–40 per 100,000 people, is more common in women than men, and may be approximately 6-fold higher in pregnant women than non-pregnant women, though this is controversial. It occurs most commonly in the 3rd trimester or peripartum period. Steroids should be avoided in the first trimester; however, acyclovir is pregnancy category B and can be started within three days of onset.^17^

### Pre-round question 6

Question: The patient has a focal nerve deficit after a seizure. What is the common name for this?

Answer: Todd’s paralysis.

Explanation: Ictal paresis and postictal paresis (Todd’s paralysis) are rare seizure manifestations. Patients present with transient motor deficits during or after a seizure. Symptoms can occur in patients presenting with first time seizure as well as recurrent epilepsy. Magnetic resonance imaging (MRI) and electroencephalogram (EEG) findings will be suggestive of seizure activity without evidence of ischemia. The deficits may last for one week after seizure, though most often duration is minutes to hours. It may be related to seizure activation or inhibition of a sensorimotor region of the brain.^18^–^20^

### Pre-round question 7

Question: What type of seizure is this?

-Stiffening of the body followed by jerking of the body
-Loss of consciousness
-Post-ictal period
-Incontinence

Answer: Tonic-clonic (grand mal)

Explanation: Tonic-clonic or grand mal seizures, also characterized as general seizures, are full-body, nonfocal seizures with loss of consciousness. Seizures lasting longer than five minutes require treatment with benzodiazepines or anticonvulsants. Most (60%-70%) patients with recurrent seizures gain symptom control with anti-epileptic medications.^21^,^22^

### Pre-round question 8

Question: A football player is knocked unconscious after he is tackled without his helmet on. He then wakes up and feels fine. He is sitting on the bench...then he becomes lethargic. What is the injury?

Answer: Epidural hematoma

Explanation: Epidural hematoma can occur from trauma of various types and is defined as a collection of blood between the dura and the skull. It appears as a lenticular shaped hematoma on imaging, usually due to injury of the middle meningeal artery or vein. Patients sustain the injury, then usually have loss of consciousness followed by a period of alertness (but often appear confused). This alert period is characterized as the lucid period. The patient may then become progressively confused and lethargic. Epidural hematomas can develop quickly leading to rapid decline in mental status. Patients with these injuries require immediate neurosurgical intervention.^23^,^24^

### Pre-round question 9

Question: A trauma patient does not have triceps function. Where is the spinal cord lesion?

Answer: C6/C7.

Explanation: Spinal cord lesions are associated with specific motor deficits. The cervical spine accounts for over 50% of spinal cord injuries. The triceps, wrists, fingers, torso, and lower limbs all have some function from the C5–C7 level. An injury at this level is expected to cause deficits in the above anatomic areas. Primary injury is caused by shearing/compression forces, associated vascular disruption, and cell death. Secondary injury is caused by ischemia, inflammation, and excitotoxicity.^25^

### Pre-round question 10

Question: What is the Glasgow Coma Scale (GCS) of the patient below?

A 23-year-old male presents after a motor vehicle accident. He opens his eyes when asked, is disoriented and confused, and is able to pinpoint the location of his pain.

Answer: GCS 12 (eyes=3, verbal=4, motor=5).

Explanation: The GCS is a widely used scoring system to assess a patient’s level of neurologic function. The GCS was initially designed for patients with head trauma but is also used to assess level of consciousness in intensive care units, emergency departments, and any other clinical care setting where critical patients are seen. The scale is calculated based on three main categories: eye opening, verbal performance, and motor responsiveness. Each category has specific criteria for each score value. A maximum value of 15 is attainable.^26^,^27^

### Pre-round question 11

Question: Name the syndrome

-Miosis
-Ptosis
-Anhidrosis

Answer: Horner’s syndrome

Explanation: Horner’s syndrome is a clinical syndrome resulting from damage to the ipsilateral oculosympathetic pathway. It consists of unilateral ptosis, ipsilateral miosis, and ipsilateral facial anhidrosis. The pupil will remain reactive. Lesions of the hypothalamus, pons, lateral medulla, lower cervical and upper thoracic spinal cord can all cause Horner’s syndrome. Horner’s syndrome can also occur as a part of a larger clinical syndrome such as Wallenberg Syndrome.^28^

### Pre-round question 12

Question: Patient has a sudden, severe, thunderclap headache. Computed tomography (CT) is negative. What is the next test?

Answer: Lumbar puncture

Explanation: Severe, sudden onset headache is also known as a thunderclap headache. It can occur due to many causes; however, the most concerning of these causes is a subarachnoid hemorrhage (SAH) due to aneurysm rupture. A broad differential should be assumed; however, sudden, non-traumatic headache onset in patients with risk factors should be evaluated for SAH. SAH is defined as bleeding into the subarachnoid space between the arachnoid membrane and pia mater. Treatment is by surgical clipping or endovascular repair. Diagnoses can be by computed tomography (CT) and/or lumbar puncture if CT is negative.^29^,^30^

### Pre-round question 13

Question: A patient exhibits the following symptoms. What is the diagnosis?

-Pill rolling tremor
-Akinesia/bradykinesia
-Rigidity
-Kyphosis
-Shuffling gait

Answer: Parkinson’s disease

Explanation: Parkinson’s disease is a progressive disease encompassing movement as well as cognitive dysfunction. The symptoms above are characteristic of some of the gait and tremor abnormalities associated with the disease. Other nonmotor symptoms include sleep disorders, constipation, and hyposmia, as well as cognitive decline and psychiatric manifestations. The progressive nature is usually slow and on the order of years to decades. Levodopa therapy remains commonplace in Parkinson’s treatment; however, is not without complications. Surgical treatments are also available and are at various stages of development.^31^

## Appendix B Round Topics with Explanation/Important Points for Learners

The following topics were used for the rounds of questions. After answering the pre-round question and determining which team will guess during the round, the slides were populated by the answers determined by the expert associations. After the team guessed the expert responses, each topic was then reviewed by the host(s). The information reviewed with the learners for each topic is discussed below. These explanations may be used with the learners or adjusted based on the host preferences.

### Round 1: Tissue plasminogen activator (tPA) contraindications

The following are absolute contraindications to giving tPA: ICH (intracerebral hemorrhage), presentation suggestive of SAH, neurosurgery, head trauma, or stroke <3 months, uncontrolled HTN (hypertension) (SBP>185 or DBP >110), history of ICH, serious head trauma or stroke within 3 months, known intracranial AVM (arteriovenous malformation), neoplasm, or aneurysm, active internal bleeding, suspected or confirmed endocarditis, known coagulopathy (platelets<100,000, heparin within the last 48 hours with elevated PTT, INR>1.7), current use of direct thrombin/Factor Xa inhibitors, and abnormal glucose (<50 or >400 mg/dL).

The following are relative contraindications to giving TPA: minor or rapidly improving symptoms, major surgery or serious (non-head) trauma within 14 days, history of GI or urinary hemorrhage within 21 days, advanced age >75 years, severe stroke (NIHSS >21) or coma, arterial puncture of non-compressible vessel, seizure at onset, myocardial infarction within 3 months, central nervous system structural lesions (AVM, aneurysm, neoplasm), and dementia.^32^

### Round 2: Optic neuritis

Optic neuritis is an inflammatory demyelinating disorder of the optic nerve often associated with multiple sclerosis (MS). It is the most common optic neuropathy affecting young adults and is more prevalent in females. Typical optic neuritis is associated with MS, whereas atypical optic neuritis is associated with neuromyelitis optica and other systemic disorders. Clinical features of typical optic neuritis include subacute monocular vision loss with pain during eye movement. Other symptoms include phosphenes, which are flashes of light connected to eye movement, the Uhthoff phenomenon, which is a worsening of vision provoked by temperature increases, the Pulfrich effect, which is a perception of objects in motion, and red color desaturation, which is when red colors appear washed out in the affected eye. Diagnosis is based on history, ophthalmologic examination, and MRI demonstrating white matter abnormalities. MRI also helps stratify the future risk of conversion to multiple sclerosis. Treatment includes high-dose methylprednisolone (500mg per day orally for 5 days or 1g per day IV for 3 days). Steroids accelerate the recovery of visual function, but have not been shown to affect long-term outcomes.^33^

### Round 3: Botulism

Botulism is a rare, but deadly disease caused from exposure to the neurotoxin released by the *Clostridium botulinum* organism. Botulinum toxin inhibits acetylcholine transmission across the neuromuscular junction at presynaptic motor neuron terminal. The most common presentations of adult botulism are related to foodborne exposure or wound infection. Infant botulism affects children less than one year of age and is caused by colonization of the immature infant gut with *C. botulinum.* Both adult and infant botulism can be characterized by symmetric cranial nerve palsies, descending flaccid paralysis, and eventually respiratory arrest. First clinical symptoms in infants may be constipation, decreased ability to suck and swallow, and neck weakness. Treatment is centered around supportive care and antitoxin therapy. Adults are treated with an equine derived antitoxin while infants are given human immunoglobulin BabyBIG.^34^

### Round 4: Giant Cell Arteritis

Giant cell arteritis (GCA) is a systemic inflammatory vasculitis. Risks for developing GCA are an age greater than 50 years, being Caucasian, and being female. Symptoms on presentation include vision loss, diplopia, headache, fatigue, and jaw claudication. Diagnosis is made by history, tenderness to palpation of the temple, and elevated ESR/CRP (erythrocyte sedimentation rate/C-reactive protein). Permanent vision loss occurs in 20% of patients. Definitive diagnosis is made by temporal artery biopsy but should not delay treatment. Treatment includes high dose steroids 40–60mg/day and should be given as soon as the diagnosis is suspected. An alternative treatment includes 1000mg methylprednisolone per day for inpatient treatment.^35^

### Round 5: Viral encephalitis

Acute viral encephalitis is a condition characterized by altered mental status accompanied by fever, seizures, and neurologic deficits. Most cases can be attributed to herpes simplex virus (HSV) followed by varicella zoster virus, enteroviruses, and arboviruses (West Nile, equine, St. Louis, Zika). Some of these viruses, like HSV, affect all age groups equally and do not demonstrate a seasonal or geographic pattern, whereas others, like arboviruses, are known to be seasonal and endemic only in certain US territories. Risk factors include traveling to or residing in an endemic area, contact with animals, insect bites, sexual practices, and immunosuppression. Symptoms of acute viral encephalitis include altered mental status, headache, nausea, vomiting, fever, focal neurologic deficits, and seizures. The presence of altered mental status helps distinguish encephalitis from meningitis. Diagnosis is confirmed by CSF (cerebrospinal fluid) analysis with PCR (polymerase chain reaction) for DNA-viruses and reverse-transcriptase PCR for RNA viruses. Early empiric therapy includes acyclovir 10–15 mg/kg IV every 8 hours.^36^

### Round 6: Neurocysticercosis

Neurocysticercosis is an infection of the central nervous system (CNS) and meninges by the larval stage of the pork tapeworm *Taenia solium*. It is diagnosed in more than 2% of patients presenting with seizures in the emergency department. The infection is acquired through ingestion of foods or drinks contaminated with *Taenia solium* eggs. The organism resides in the small bowel before migrating to muscles and the CNS. Because the infection is not endemic in the United States, it is typically found in immigrants from endemic countries (Latin American countries, Asia, sub-Saharan Africa). It can affect any age group from infancy to old age, with a peak incidence at ages 20–50 years.

Neurocysticercosis is known as the great imitator because it can mimic almost any neurologic disorder. Common clinical manifestations include seizures, focal neurological deficits, increased intracranial pressure, and cognitive decline. Diagnosis is made with CT and MRI of the brain demonstrating the characteristic ring-enhancing or calcified lesions, and serologic testing (enzyme-linked immunoelectrotransfer blot) is used to confirm the diagnosis. Prior to initiation of therapy, all patients should have an ophthalmologic examination to rule out ocular cysticercosis, which can lead to vision loss. Treatment includes antiparasitic therapy (for one to two cysts: albendazole 15mg/kg in two daily doses; for more than two cysts: albendazole 15mg/kg in two daily doses plus praziquantel 50 mg/kg in three daily doses) for a duration of 10 to 14 days, and adjunctive corticosteroids (prednisone 1mg/kg or dexamethasone 0.1mg/kg per day) begun at least one day prior to the antiparasitic agents to decrease inflammation and seizure risk. Prognosis is worse with a greater number of brain lesions and extent of inflammation.^37^

### Round 7: Rabies

The rabies virus is a Lyssavirus which is transmitted through saliva, usually in a bite from a rabid animal. Animals implicated in rabies transmission include foxes, bats, dogs, cats, coyotes, wolves, skunks, groundhogs, and raccoons. Animals which do not transmit rabies include squirrels, chipmunks, mice, and rats. Cases of animal and human rabies are reported in the United States; however, 92% of animal cases are from wildlife rather than domesticated animals. Domesticated cases are primarily from cats. Human cases have also been transmitted by organ donation from an infected donor. Mortality is nearly 100% after symptom development. Symptoms on presentation include paresthesia at the bite site, paralysis, weakness, encephalitis, or altered mental status/aggressive behavior. The disease is rapidly progressive in most cases. Patients with an exposure should receive immediate wound washing and 20 IU/kg of human rabies immunoglobulin (HRIG) at the bite site and in the gluteal area. For previously unvaccinated patients, inactivated vaccine should be given on days 0, 3, 7, and 14 intramuscularly in the deltoid or thigh. Previously vaccinated patients should be given booster doses on days 0 and 3. Patients at high risk for exposure should be vaccinated.^38^,^39^

### Round 8: Myasthenia gravis

Myasthenia gravis is an autoimmune disease affecting the neuromuscular junction and is characterized by fatigable weakness of the skeletal muscles. It is caused by autoantibodies directed against acetylcholine receptors and other proteins in the postsynaptic membrane. The onset is usually in young adulthood and more often affects females. There is a strong association with thymomas. Clinical features include fatigable weakness that improves with rest. Extraocular muscles are usually affected first leading to diplopia and ptosis. Other muscle groups can also be affected leading to proximal limb weakness and dysphagia. Severe exacerbations causing respiratory insufficiency are termed myasthenic crises. Sensory and autonomic dysfunction do not occur in myasthenia gravis.

Diagnosis is based on history, neurologic examination, serologic studies, and electrophysiologic testing. The ice pack test, which involves applying ice to the eyelid, can be used to support the diagnosis if ptosis improves. The edrophonium or Tensilon test is no longer used in the United States, which involves administering an acetylcholinesterase inhibitor to assess for improvement of muscle weakness. Treatment includes acetylcholinesterase inhibitors (pyridostigmine), immunosuppressive agents, and thymectomy. Excess acetylcholinesterase inhibition can lead to paradoxical weakness mimicking a myasthenic crisis, which is termed a cholinergic crisis. This can be ruled out by temporarily stopping anticholinesterase agents. For myasthenic crises, IVIG (intravenous immune globulin) and plasmapheresis can be used.^40^

### Round 9: Neurosyphilis

Syphilis is a chronic bacterial infection caused by Treponema pallidum. There are five million new cases diagnosed every year worldwide, with most infections occurring in low and middle-income countries. With the exception of congenital syphilis, syphilis is spread through direct contact with lesions. There is typically a three-week incubation period before the appearance of primary syphilis (painless chancre). Without intervention at this time, the organism can disseminate through the bloodstream and progress to secondary syphilis (characteristic rash, condyloma lata, systemic manifestations), a latent period, and then late syphilis (neurologic, cardiovascular, gummatous).

Clinical manifestations of neurosyphilis can include ocular involvement (uveitis), aseptic meningitis, seizures, and focal neurologic deficits. Features of late neurosyphilis include general paresis, dementia, and tabes dorsalis, which results from involvement of the posterior columns and spinal nerve roots. Tabes dorsalis presents as radicular pain and ataxia due to loss of proprioception. Diagnosis is made by history, neurologic exam findings (Argyll Robertson pupils, loss of reflexes, impaired vibratory sense), and serologic testing. Serologic testing is used to confirm the presence of syphilis using a nontreponemal venereal disease research lab test (VDRL), rapid plasma reagin test (RPR) and fluorescent treponemal antibody absorption (FTA-ABS) testing followed by lumbar puncture (CSF VDRL and FTA-ABS) if neurosyphilis is suspected. Treatment for neurosyphilis is with IV penicillin G (2–4 million units every 4 hours) or IM penicillin G (2.4 million units) plus probenecid (500mg orally four times a day) for at least 10 days.^41^

### Round 10: Status Epilepticus

Status epilepticus is defined as 30 minutes or greater of prolonged epileptic activity or two or more sequential seizures without full recovery. It is diagnosed by recognition of these defining features. There is no diagnostic test for status epilepticus. Treatment includes management of airway, breathing, and circulation. Treatment of the seizures includes benzodiazepines, anticonvulsants (such as phenytoin, fosphenytoin, and levetiracetam), barbiturates (such as phenobarbital), and anesthetics (such as propofol).^22^,^42^,^43^

### Round 11: Idiopathic Facial Nerve Palsy/Bell’s Palsy

Idiopathic facial nerve palsy, or Bell’s palsy, is the most common acute mononeuropathy caused by a dysfunction of the facial nerve. It presents with rapid (<72 hours) onset unilateral facial weakness that may be partial or complete. It affects both sides of the face with equal frequency, and in rare cases (0.3%) can occur bilaterally. Although the etiology remains unknown, limited evidence suggests that reactivation of herpes simplex virus, or less commonly, varicella zoster virus can provoke facial nerve paralysis. Both sexes are equally affected with the peak incidence between ages 15 and 45. Risk factors include pregnancy, diabetes, upper respiratory tract infection, and immunocompromised states. Patients usually present with inability to close the eye, dysfunctional tear production, drooping of the corner of the mouth, eyebrow sagging and disappearance of the nasolabial fold. Additional symptoms include decreased taste, hyperacusis, and change in facial sensation. If significant pain is present, the condition is defined as Ramsey-Hunt syndrome, which is believed to be caused by varicella zoster virus.

Diagnosis is made by history, clinical presentation, and neurologic examination. It should be a diagnosis of exclusion after ruling out other causes of facial nerve palsy. If no improvement is seen within 3 weeks of symptom onset or if the condition worsens, additional tests such as contrast-enhanced CT, MRI, and electromyography are needed. Initial treatment includes high-dose oral corticosteroids within 72 hours of symptom onset. If corneal dryness and irritation develop due to incomplete closure of the eyelid and decreased tearing, artificial tears and taping the eyelid shut at night are recommended, as well as an ophthalmologist consultation. Bell’s palsy is typically a self-limited condition with 71% of cases resolving completely.^44^–^46^

### Round 12: Dementia vs. Delirium

Delirium is characterized by an acute onset (hours to days) reversible disturbance in attention, awareness, and cognition that fluctuates throughout the course of the day, represents a change from baseline, and cannot be better explained by a pre-existing neurologic condition. The prevalence of delirium in patients aged 65 and over in the emergency department has been reported to be as high as 10%. The pathophysiology of delirium is poorly understood, but is usually precipitated by a medical condition, substance intoxication or withdrawal, or medication side effect. Diagnosis is based on history (often obtained by proxy), clinical features, and laboratory testing to determine the underlying cause. If the workup is negative, then neuroimaging, lumbar puncture, and EEG may be considered. Treatment involves frequently orienting the patient, diagnosing and treating underlying illnesses, and avoiding known triggers, such as medications, immobilization, and sleepwake cycle disturbances. If the patient’s agitation is interfering with care or safety, a low-dose neuroleptic like haloperidol (0.5 to 1.0mg) can be used.

In contrast to delirium, the disturbances seen in dementia are irreversible, gradually occur over a longer period of time (months to years), and are progressive without fluctuation. Dementia is caused by a neurodegenerative disease, most commonly Alzheimer’s disease. Although memory and comprehension are impaired in patients with dementia, attention, orientation, and organized speech are typically preserved. Dementia is diagnosed by history, cognitive exams, and neuroimaging in select patients. Screening for depression is recommended. Treatment for dementia is based on the underlying etiology. Delirium can coexist with dementia which makes differentiating the two diagnoses difficult.^47^–^49^

### Round 13: Acute Inflammatory Demyelinating Polyneuropathy (AIDP/GBS)

Guillain-Barré syndrome (GBS) is the most common cause of acute flaccid symmetrical paralysis of the limbs and areflexia in the post-polio era. Risk of developing the syndrome is increased in the elderly, males, and those who have had a respiratory or gastrointestinal illness within the past six weeks. The pathogenesis of the syndrome is poorly understood, but is currently believed to result from an autoimmune response directed against myelin and axons triggered by a preceding infection. In the past, it was proposed that vaccines may trigger the syndrome; however, recent studies have shown there is little evidence to support a causal association. There are multiple subphenotypes of the syndrome, each with a different clinical presentation. Common presenting symptoms include a progressive, symmetric ascending weakness that typically starts in the lower limbs, areflexia, cranial nerve palsies, and autonomic dysfunction. Severe respiratory muscle weakness requiring ventilatory support occurs in 10%–30% of cases. An FVC <20mL/kg or NIF <30cm H2O is associated with pending respiratory failure. Diagnosis is based on history, clinical presentation, CSF analysis showing an albumin-cytological dissociation and abnormal nerve conduction studies. Treatment includes plasmapheresis and IVIG.^50^,^51^
