# Supplementary material for: Family Game Show-style Didactic for Teaching Nervous System Disorders during Emergency Medicine Training
Source: J Educ Teach Emerg Med. 2020 Apr 15;5(2):L1–L19. doi: 10.21980/J8D357 (PMC10332565; doi:10.21980/J8D357)
Supplement: Supplementary file 1 — Please see associated PowerPoint file [file jetem-5-2-l1-supp1.pptx]

## Slide 1
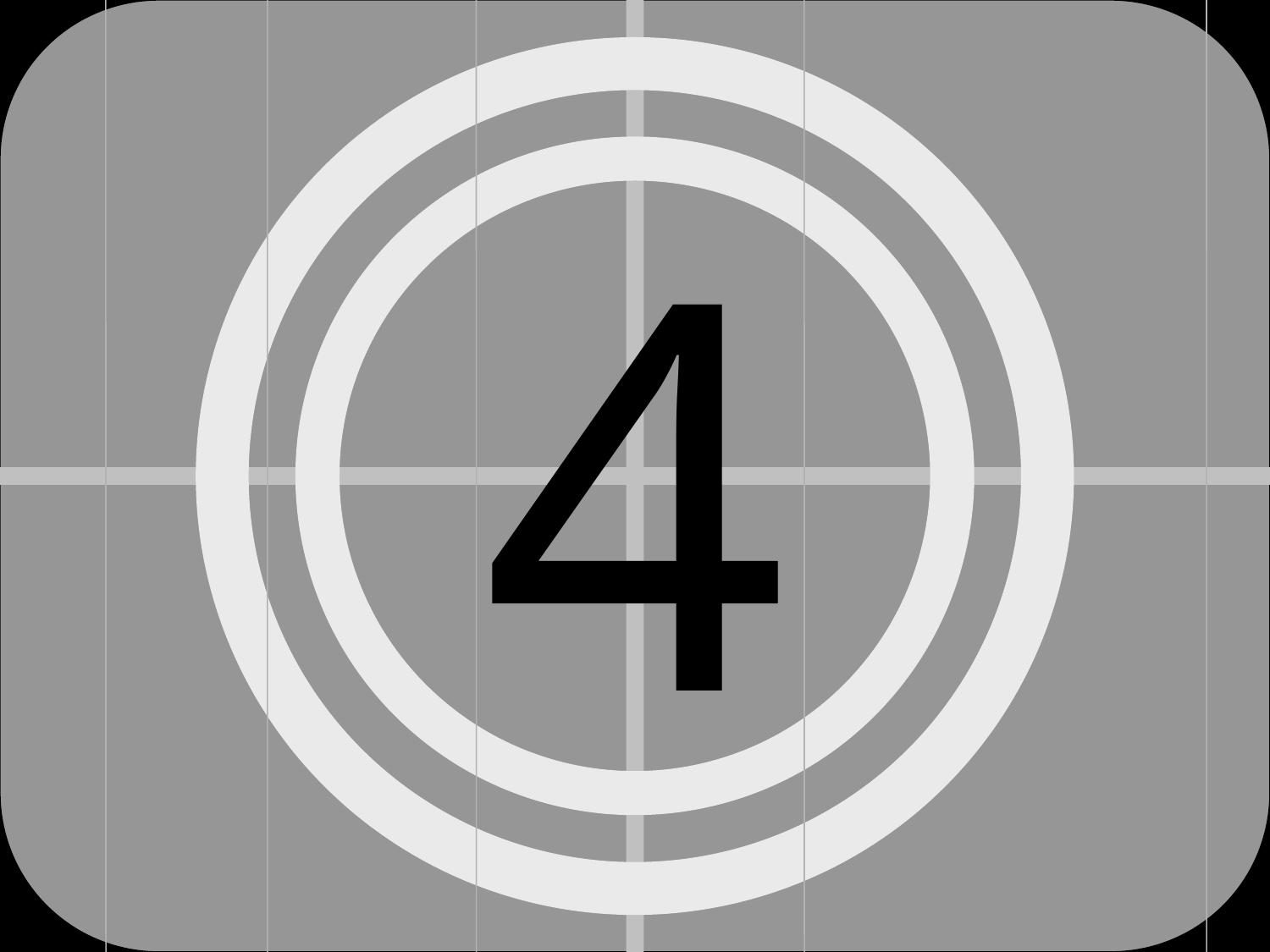

1
1
2
2
3
3
4
4

## Slide 2
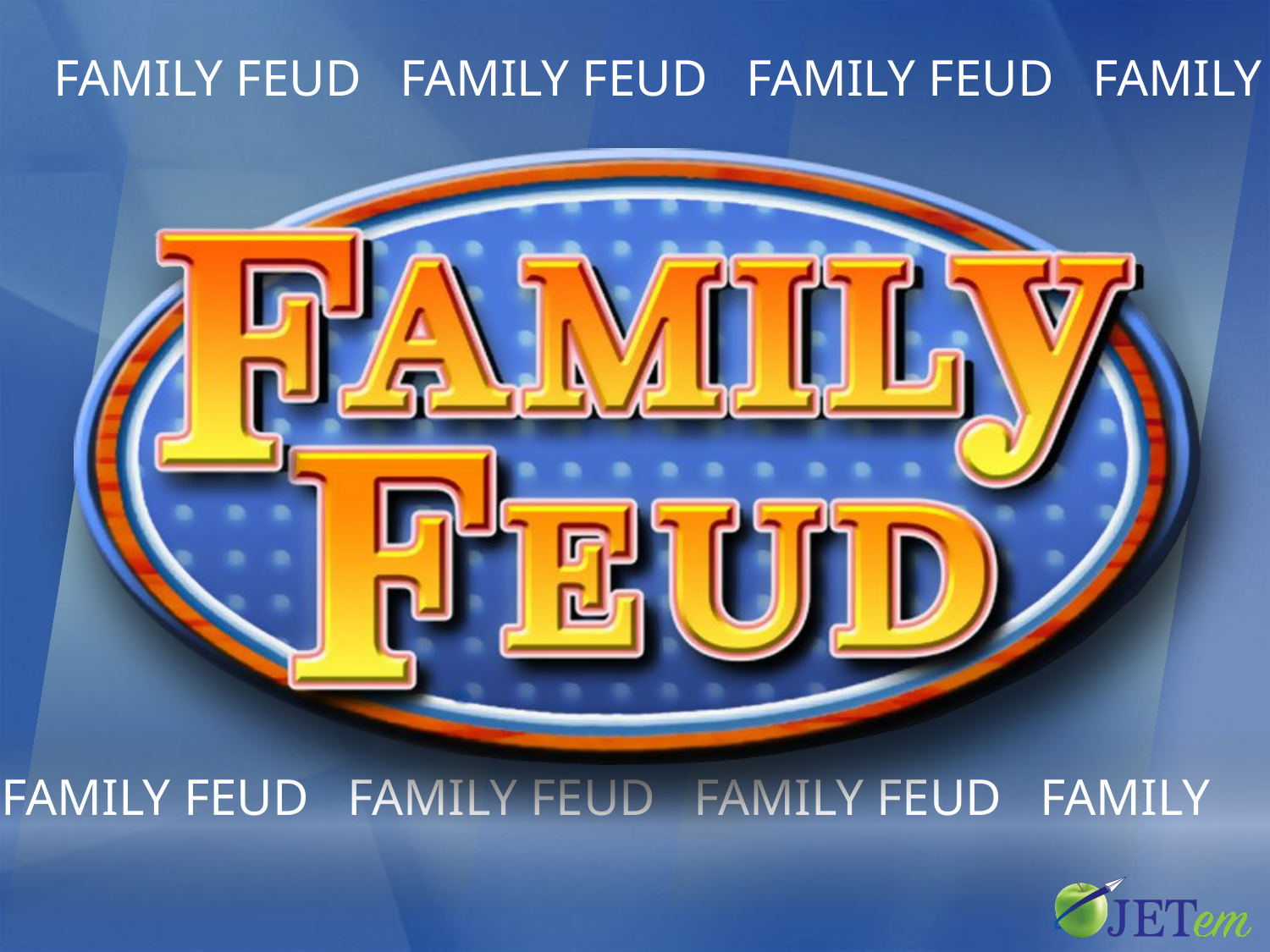

FAMILY FEUD FAMILY FEUD FAMILY FEUD FAMILY FEUD FAMILY FEUD FAMILY FEUD FAMILY FEUD FAMILY FEUD FAMILY FEUD FAMILY FEUD FAMILY FEUD FAMILY FEUD FAMILY FEUD FAMILY FEUD FAMILY FEUD FAMILY FEUD FAMILY FE
FAMILY FEUD FAMILY FEUD FAMILY FEUD FAMILY FEUD FAMILY FEUD FAMILY FEUD FAMILY FEUD FAMILY FEUD FAMILY FEUD FAMILY FEUD FAMILY FEUD FAMILY FEUD FAMILY FEUD FAMILY FEUD FAMILY FEUD FAMILY FEUD FAMILY FE

## Slide 3
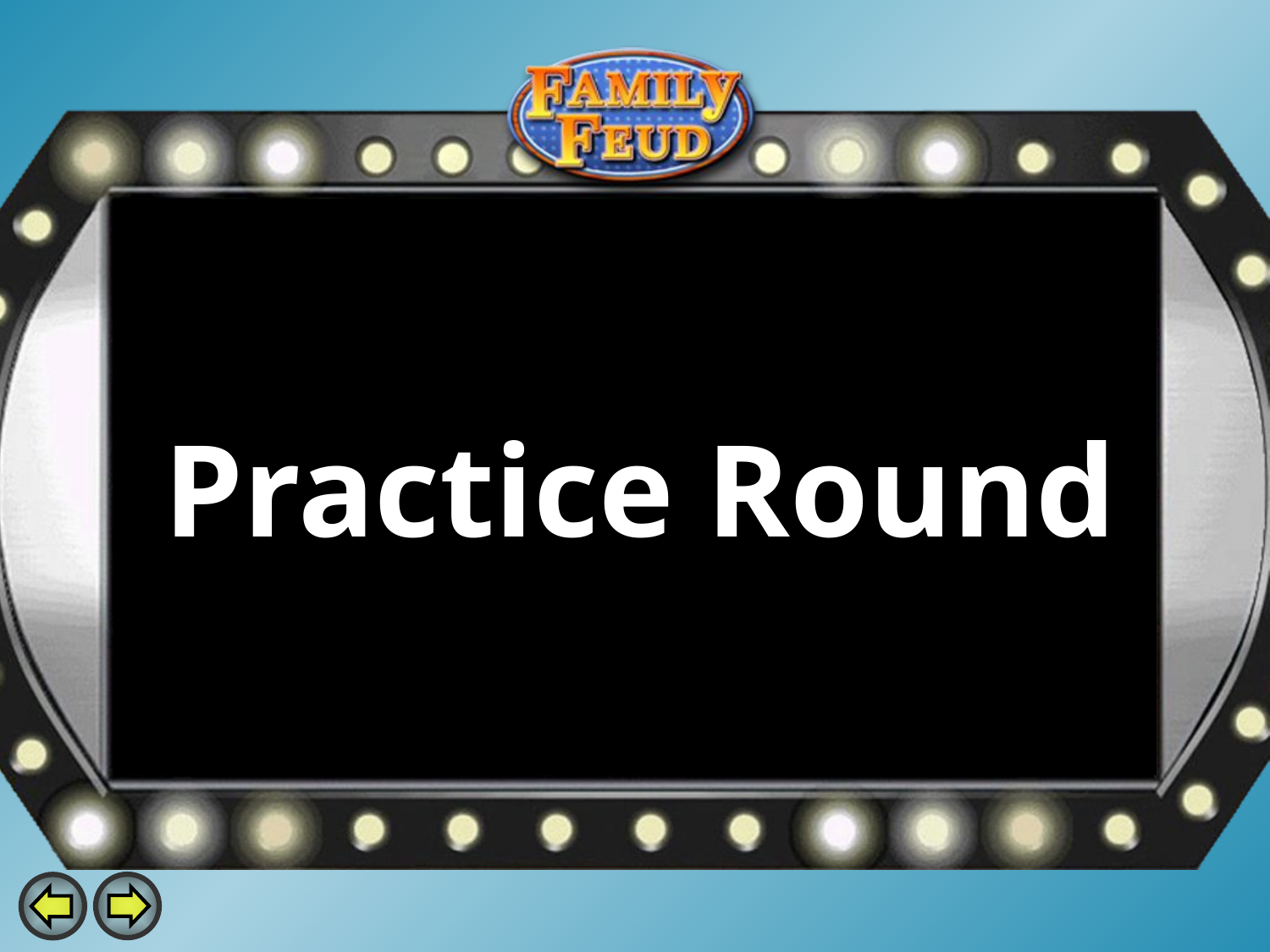

Practice Round

## Slide 4
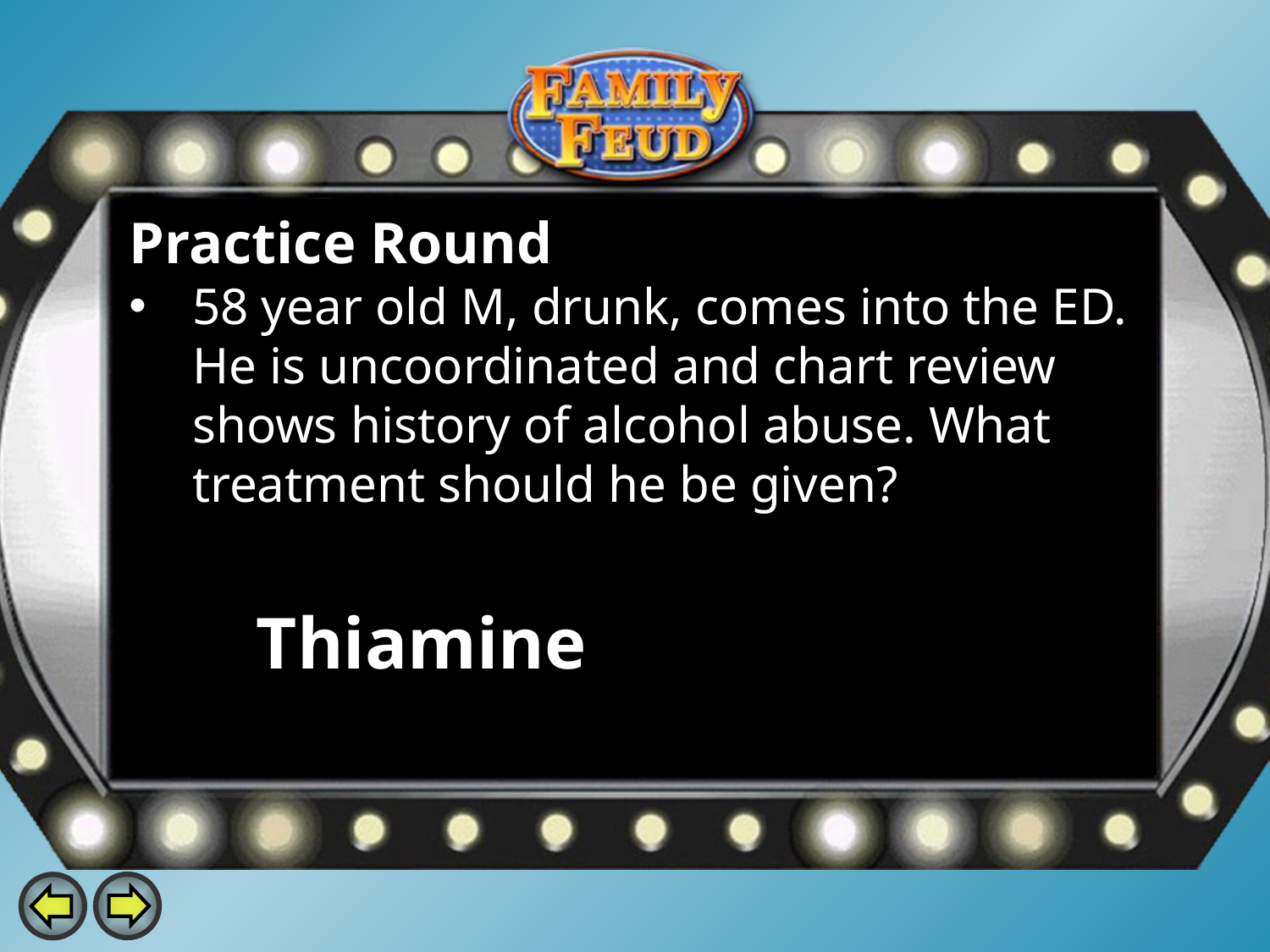

Practice Round
58 year old M, drunk, comes into the ED. He is uncoordinated and chart review shows history of alcohol abuse. What treatment should he be given?
Thiamine

## Slide 5
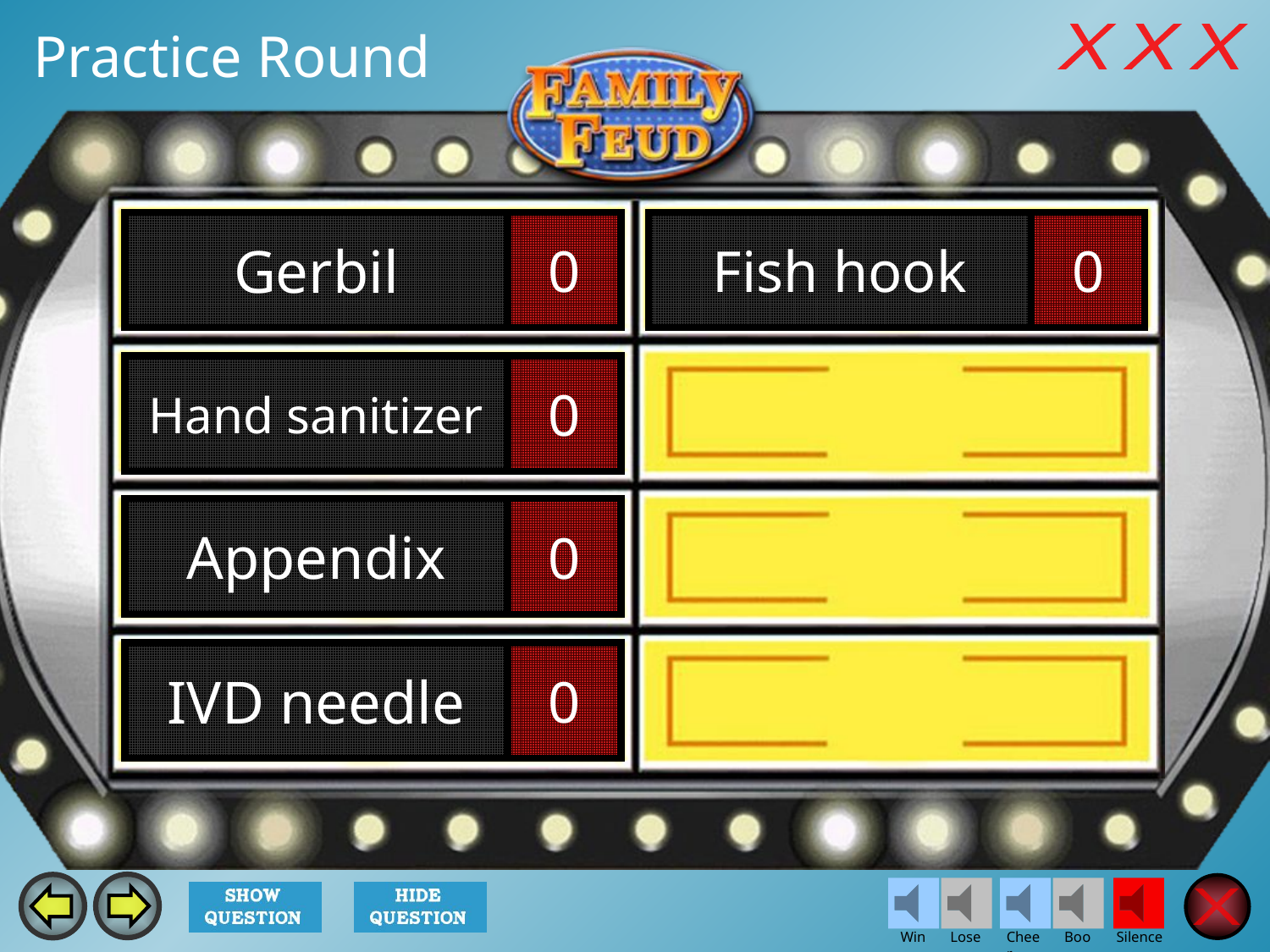

Things a doctor might pull out of a patient
X
X
X
X
X
X
Practice Round
X
X
X
Gerbil
0
Fish hook
0
Hand sanitizer
0
Appendix
0
IVD needle
0
Win
Lose
Cheer
Boo
Silence
X
X
X

## Slide 6
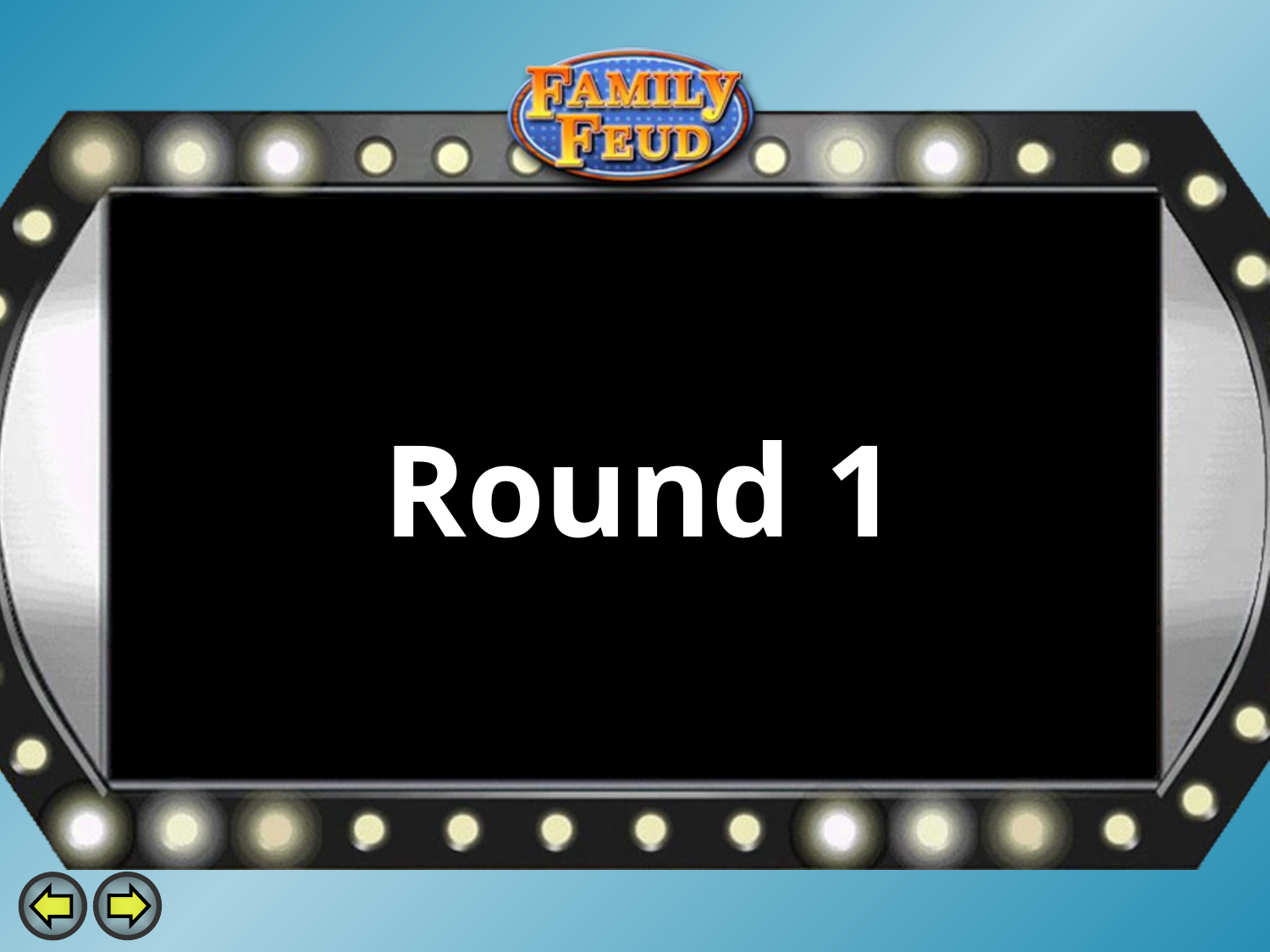

Round 1

## Slide 7
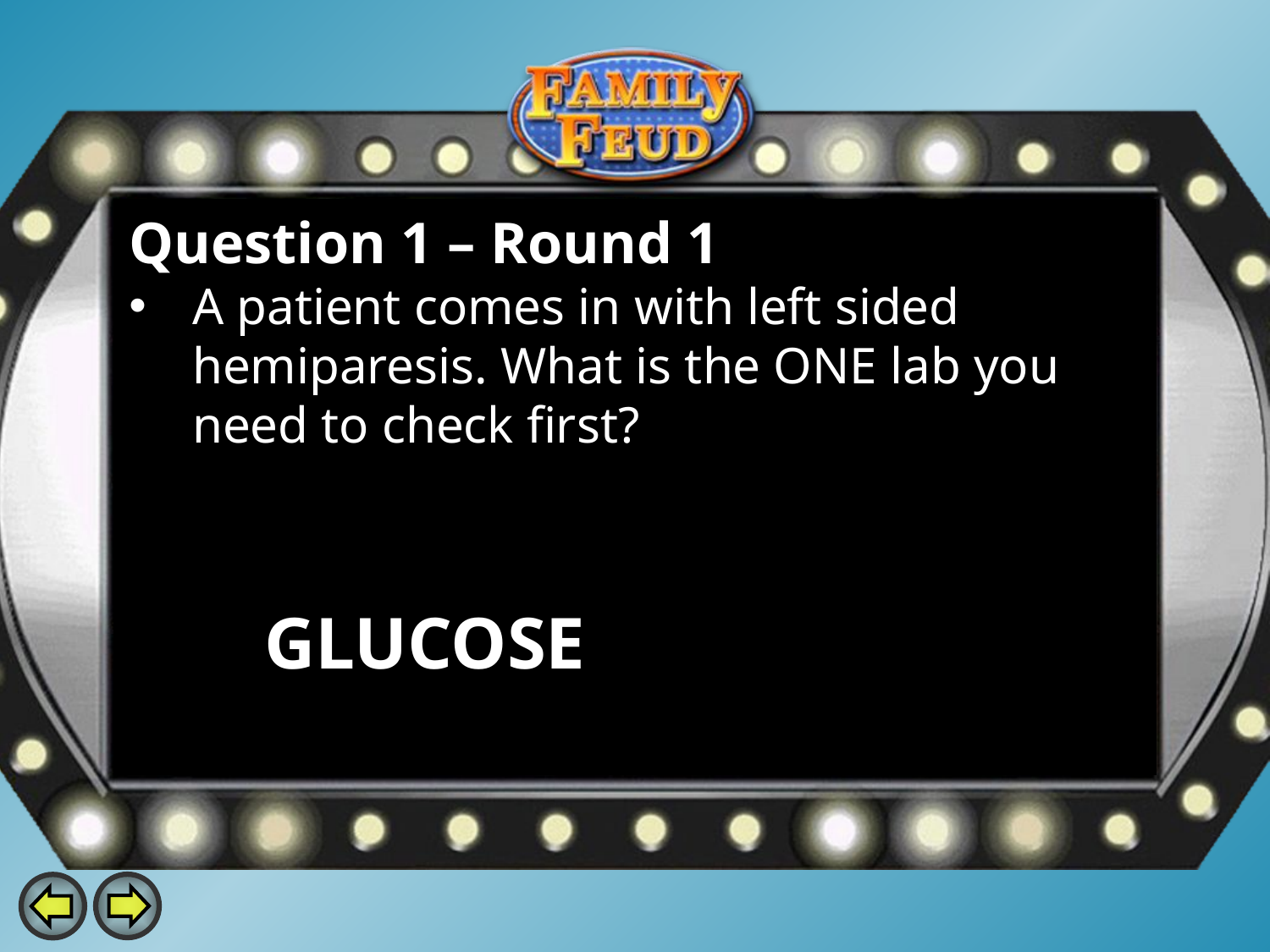

Question 1 – Round 1
A patient comes in with left sided hemiparesis. What is the ONE lab you need to check first?
GLUCOSE

## Slide 8
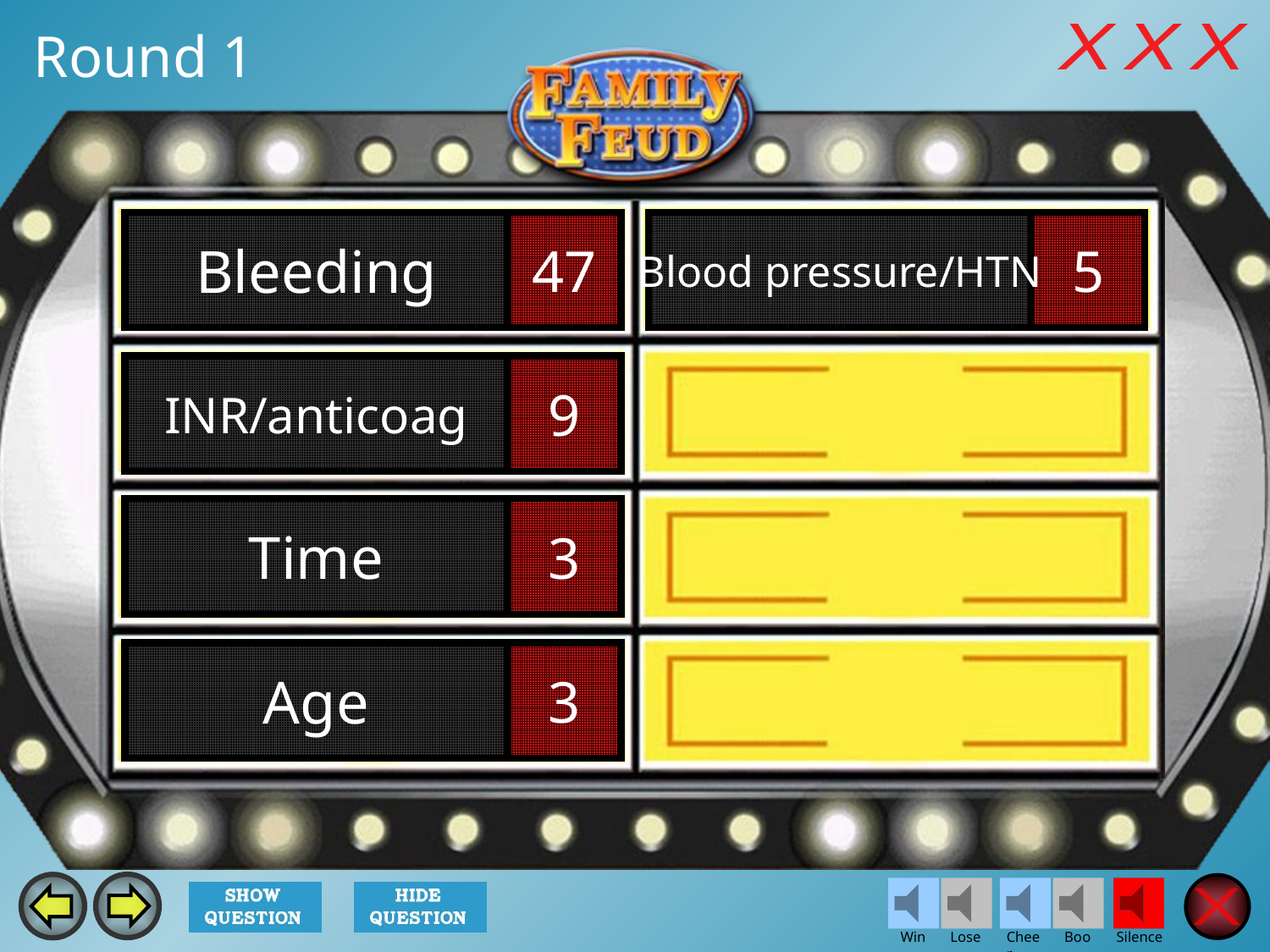

Contraindications to TPA
X
X
X
X
X
X
Round 1
X
X
X
Bleeding
47
Blood pressure/HTN
5
INR/anticoag
9
Time
3
Age
3
Win
Lose
Cheer
Boo
Silence
X
X
X

## Slide 9
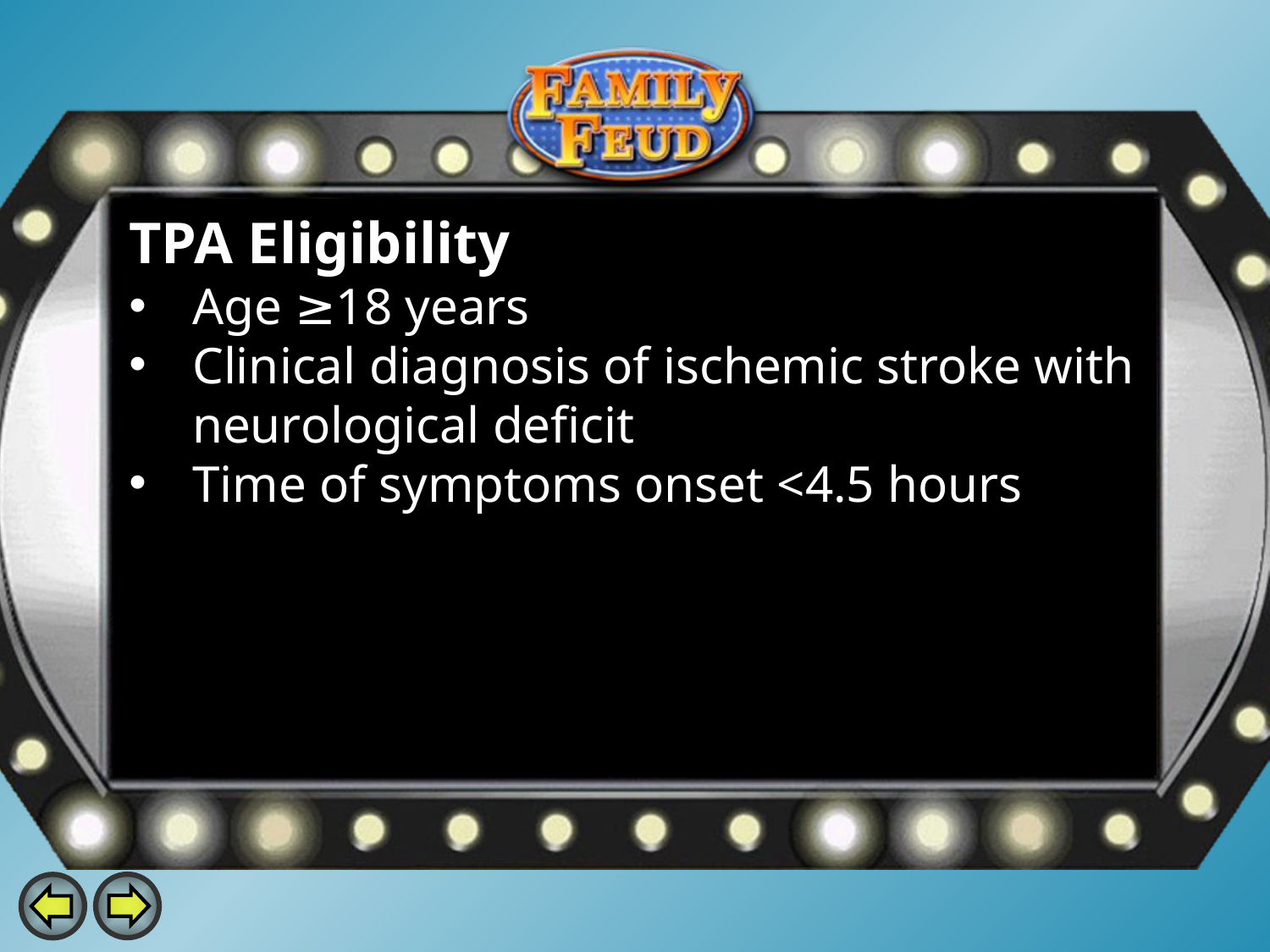

TPA Eligibility
Age ≥18 years
Clinical diagnosis of ischemic stroke with neurological deficit
Time of symptoms onset <4.5 hours

## Slide 10
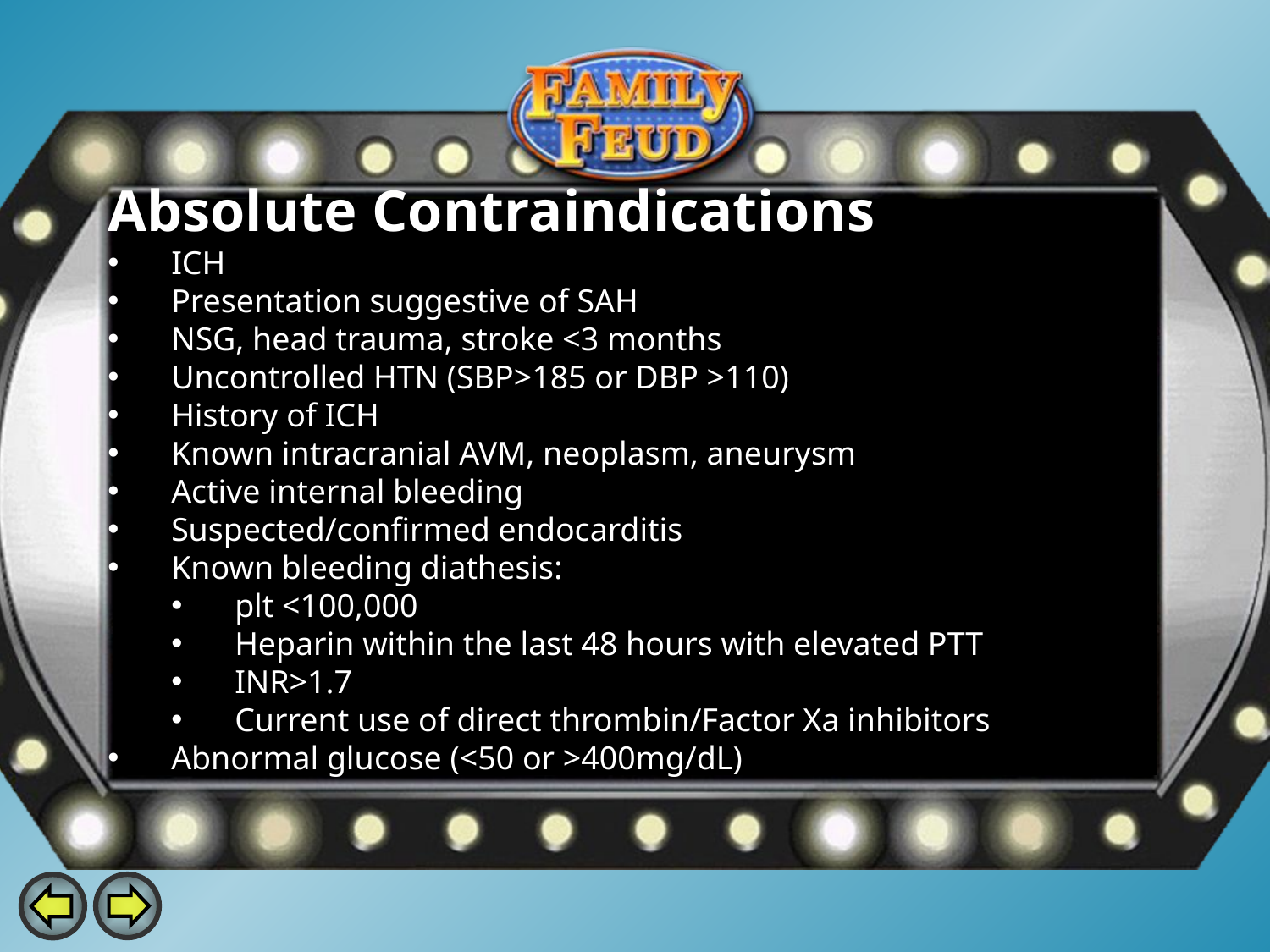

Absolute Contraindications
ICH
Presentation suggestive of SAH
NSG, head trauma, stroke <3 months
Uncontrolled HTN (SBP>185 or DBP >110)
History of ICH
Known intracranial AVM, neoplasm, aneurysm
Active internal bleeding
Suspected/confirmed endocarditis
Known bleeding diathesis:
plt <100,000
Heparin within the last 48 hours with elevated PTT
INR>1.7
Current use of direct thrombin/Factor Xa inhibitors
Abnormal glucose (<50 or >400mg/dL)

## Slide 11
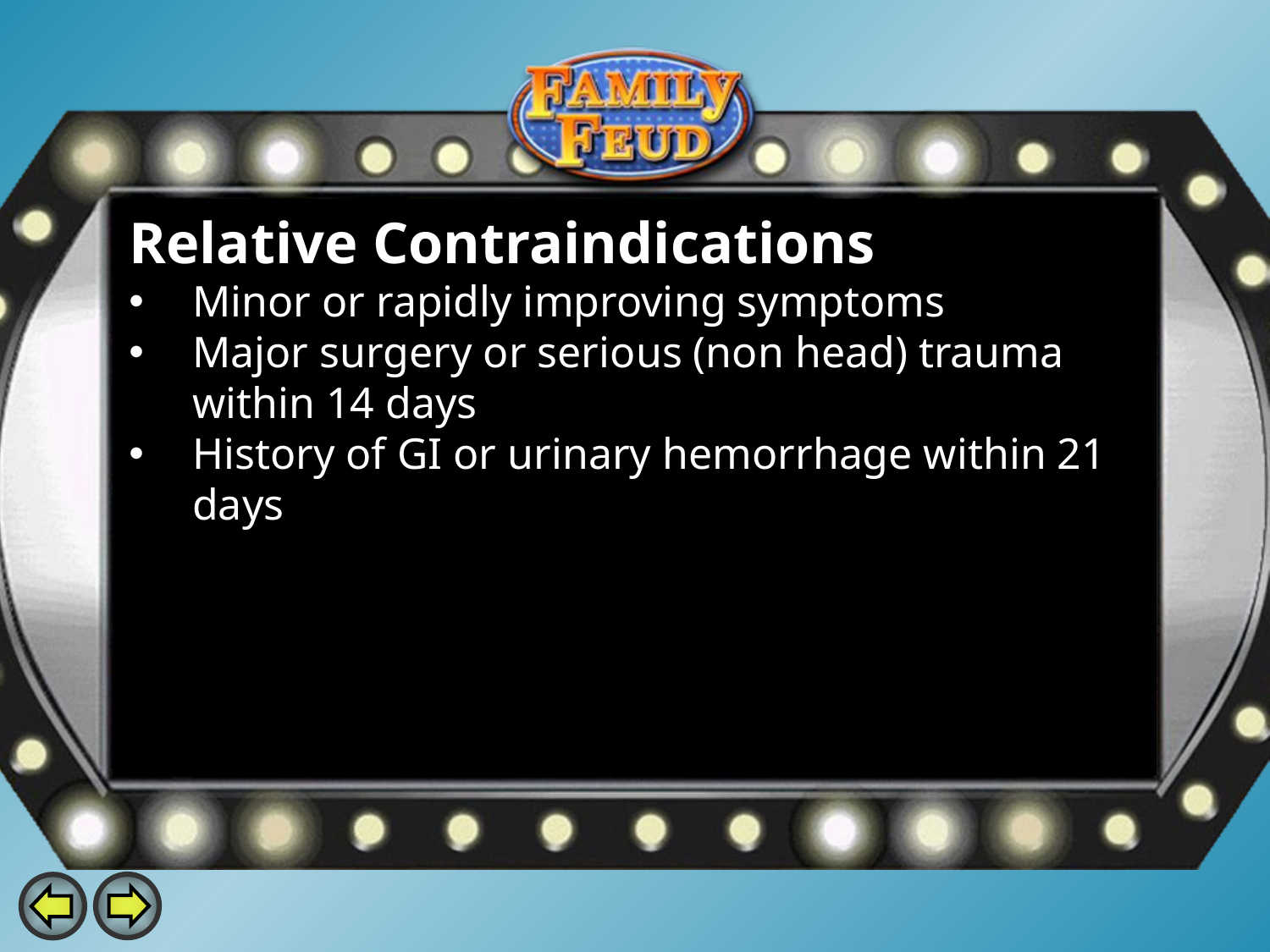

Relative Contraindications
Minor or rapidly improving symptoms
Major surgery or serious (non head) trauma within 14 days
History of GI or urinary hemorrhage within 21 days

## Slide 12
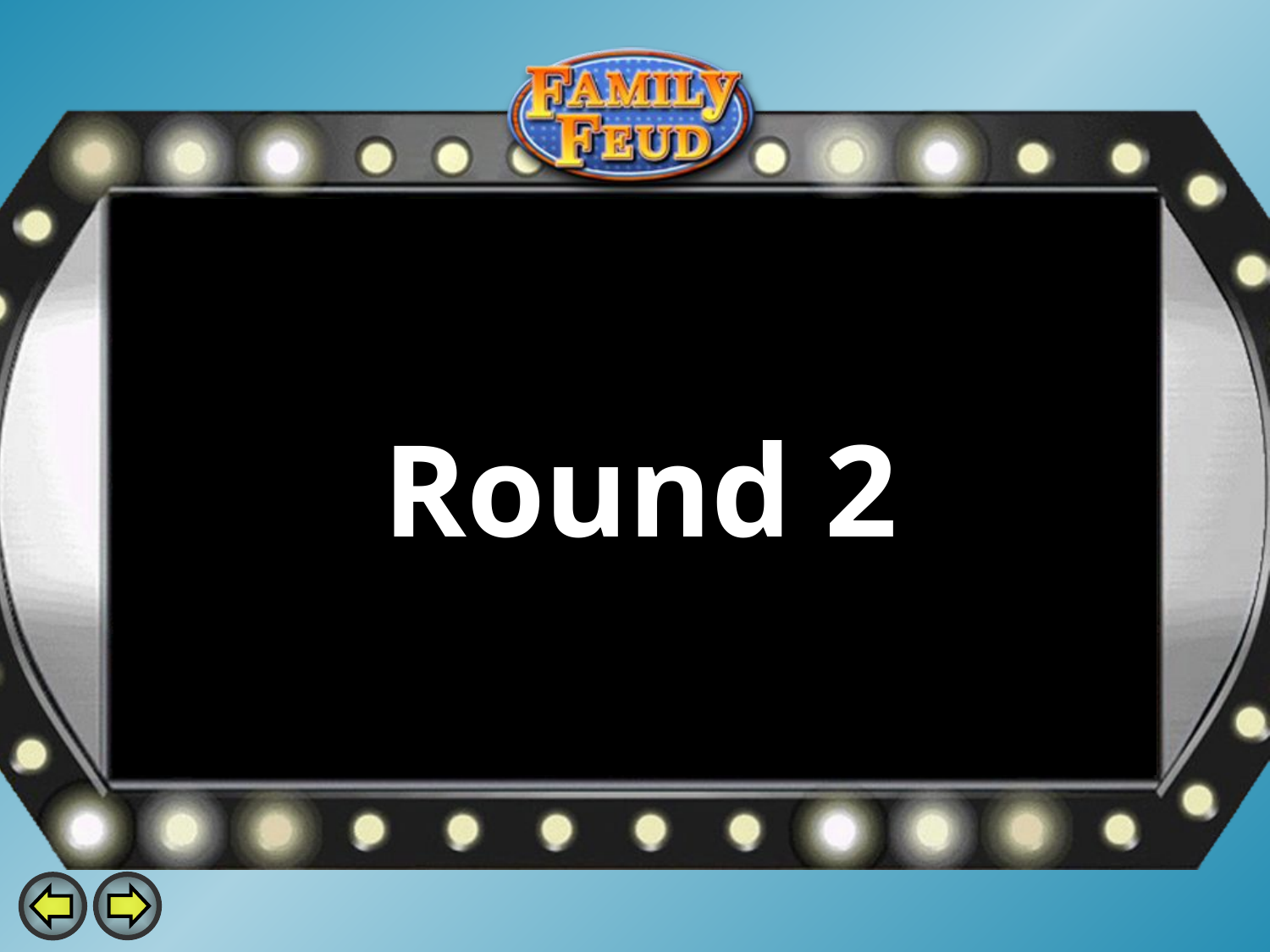

Round 2

## Slide 13
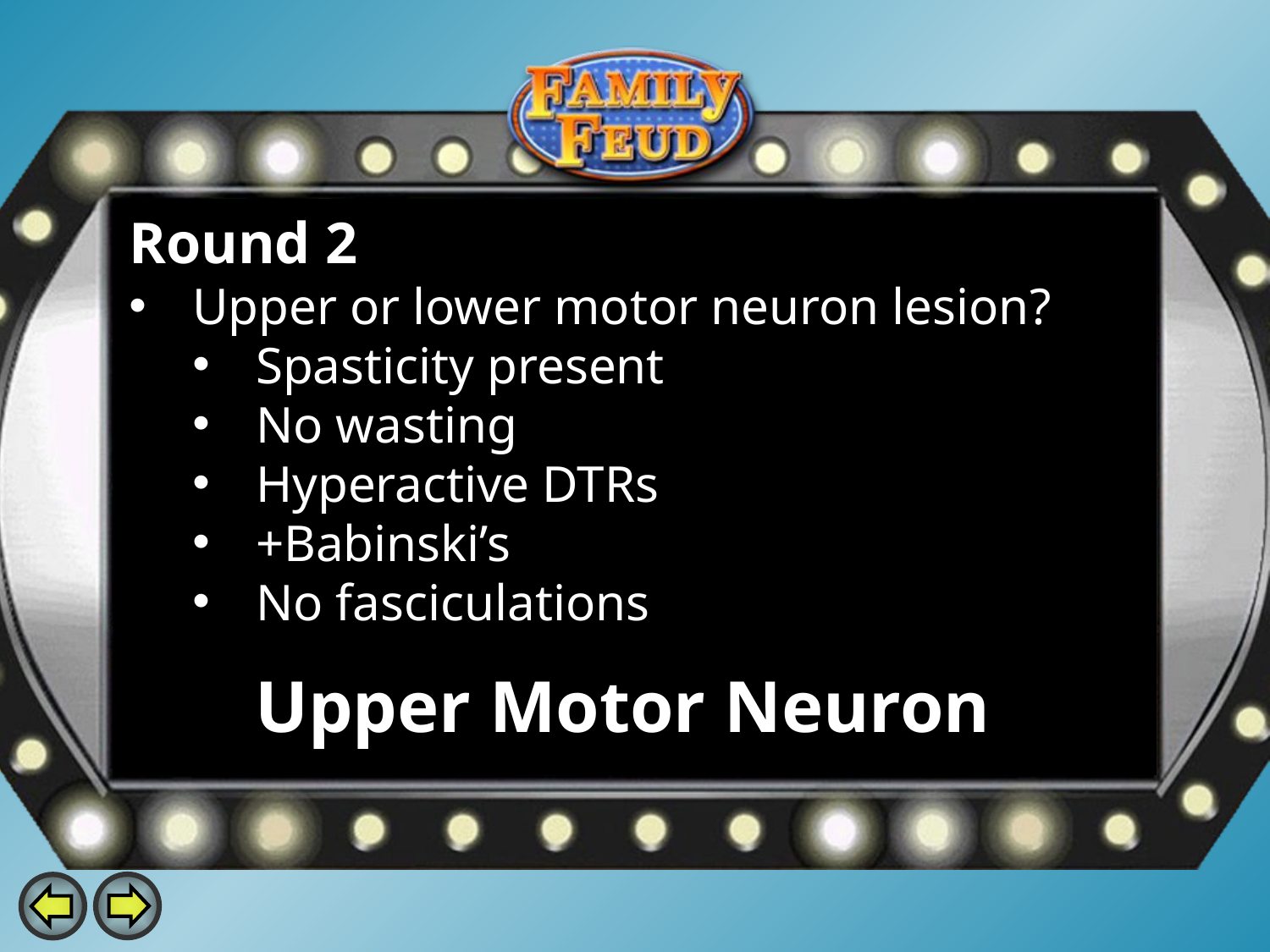

Round 2
Upper or lower motor neuron lesion?
Spasticity present
No wasting
Hyperactive DTRs
+Babinski’s
No fasciculations
Upper Motor Neuron

## Slide 14
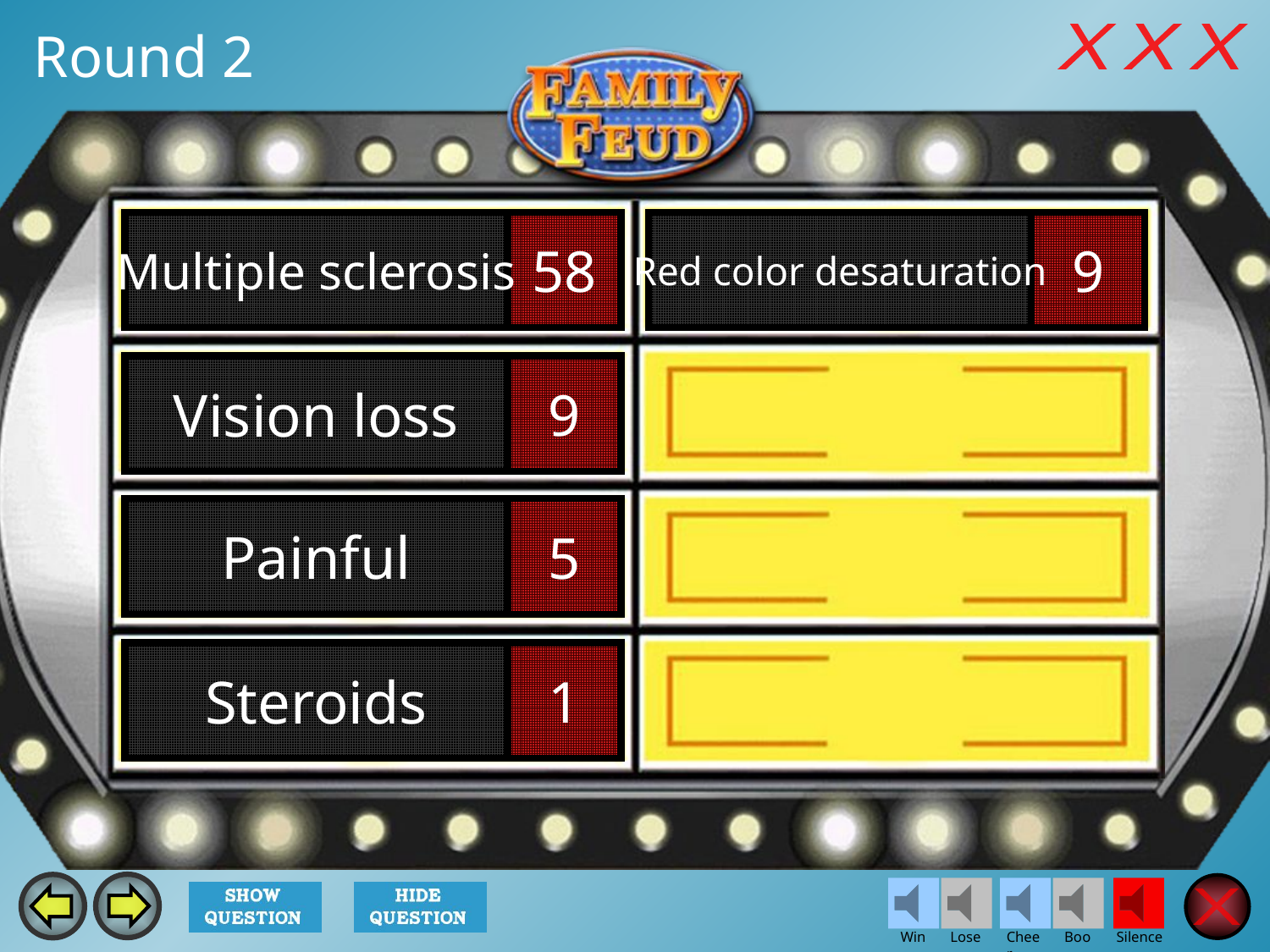

Five things suggestive of optic neuritis
X
X
X
X
X
X
Round 2
X
X
X
Multiple sclerosis
58
Red color desaturation
9
Vision loss
9
Painful
5
Steroids
1
Win
Lose
Cheer
Boo
Silence
X
X
X

## Slide 15
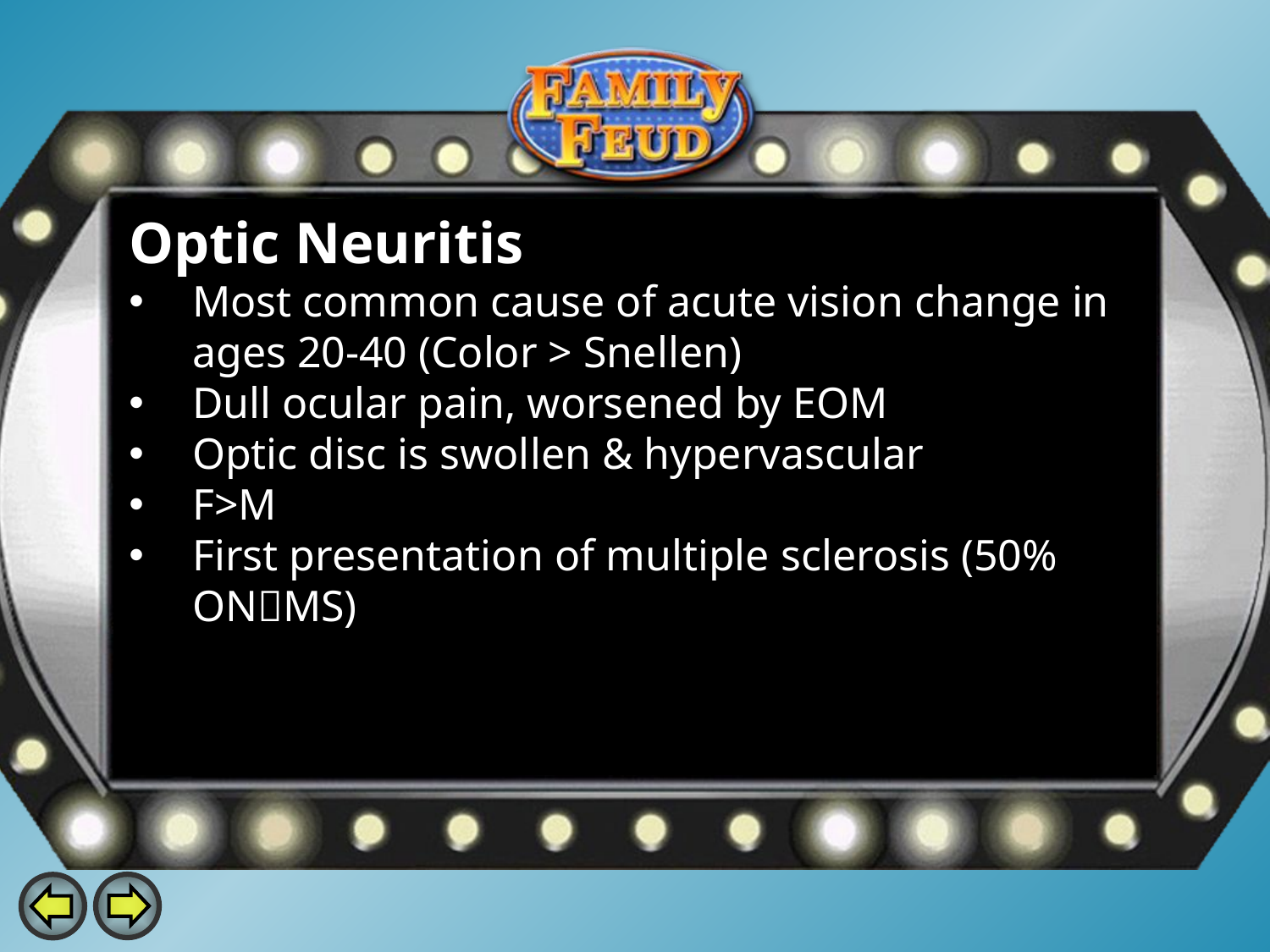

Optic Neuritis
Most common cause of acute vision change in ages 20-40 (Color > Snellen)
Dull ocular pain, worsened by EOM
Optic disc is swollen & hypervascular
F>M
First presentation of multiple sclerosis (50% ONMS)

## Slide 16
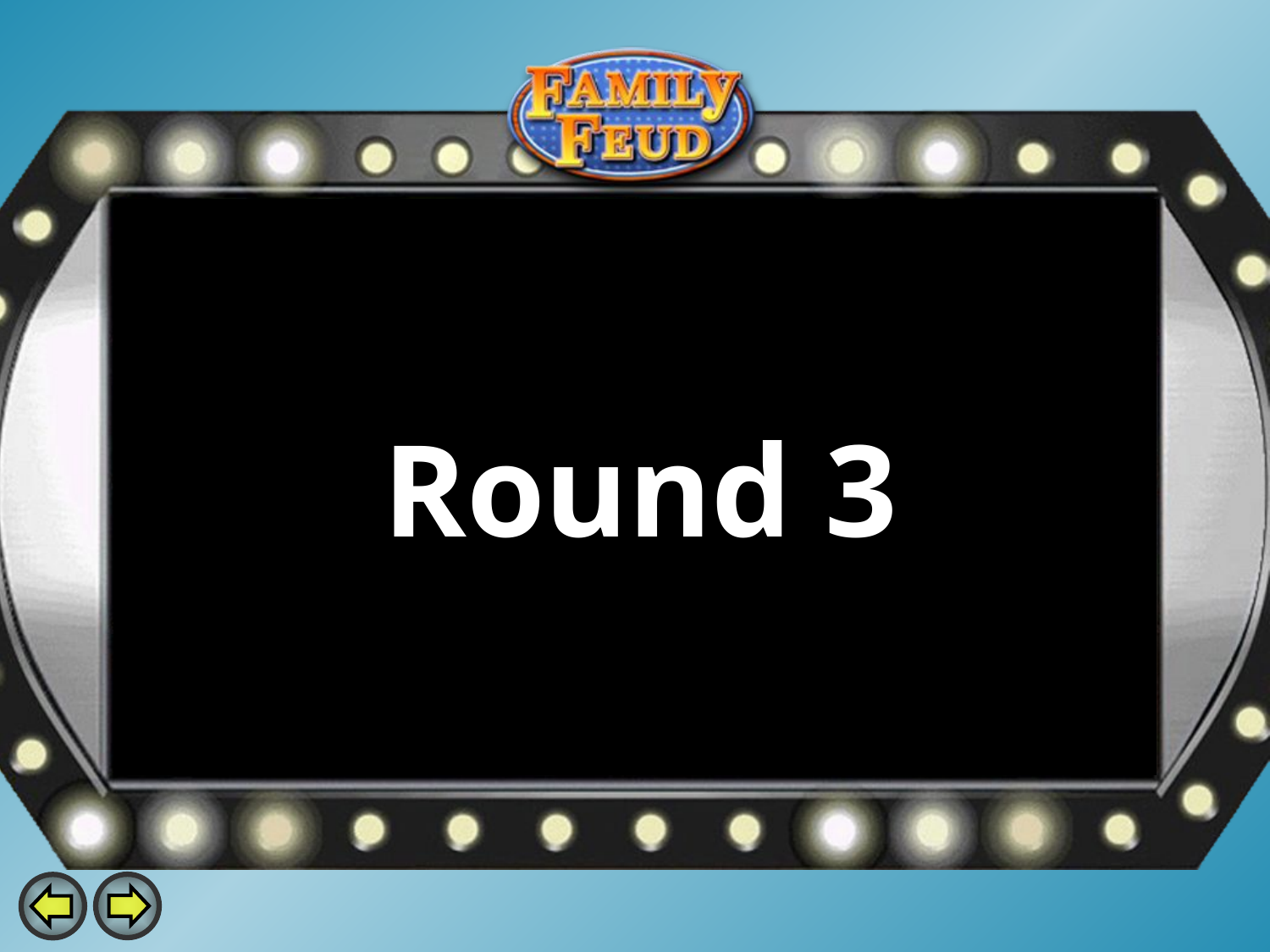

Round 3

## Slide 17
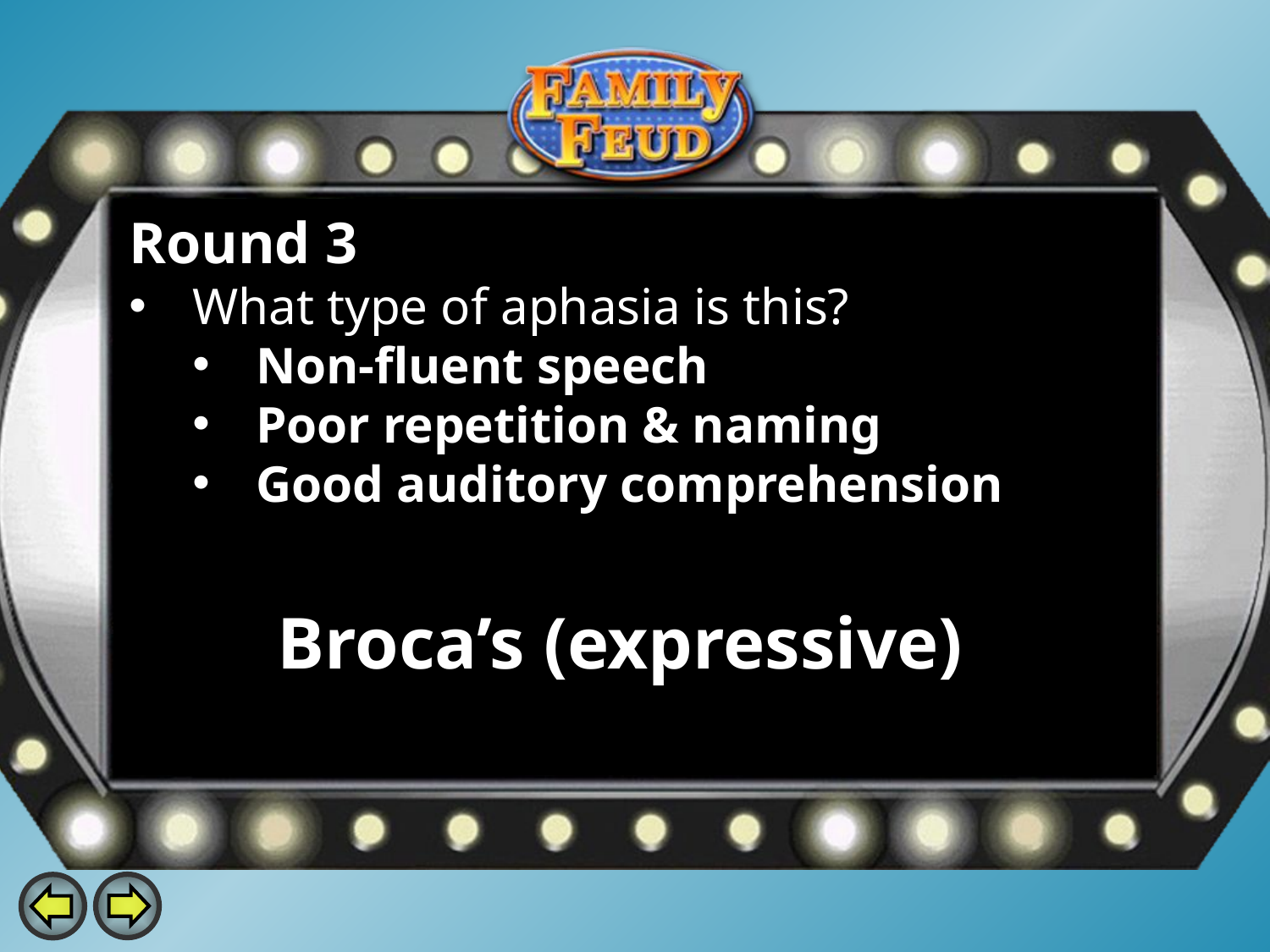

Round 3
What type of aphasia is this?
Non-fluent speech
Poor repetition & naming
Good auditory comprehension
Broca’s (expressive)

## Slide 18
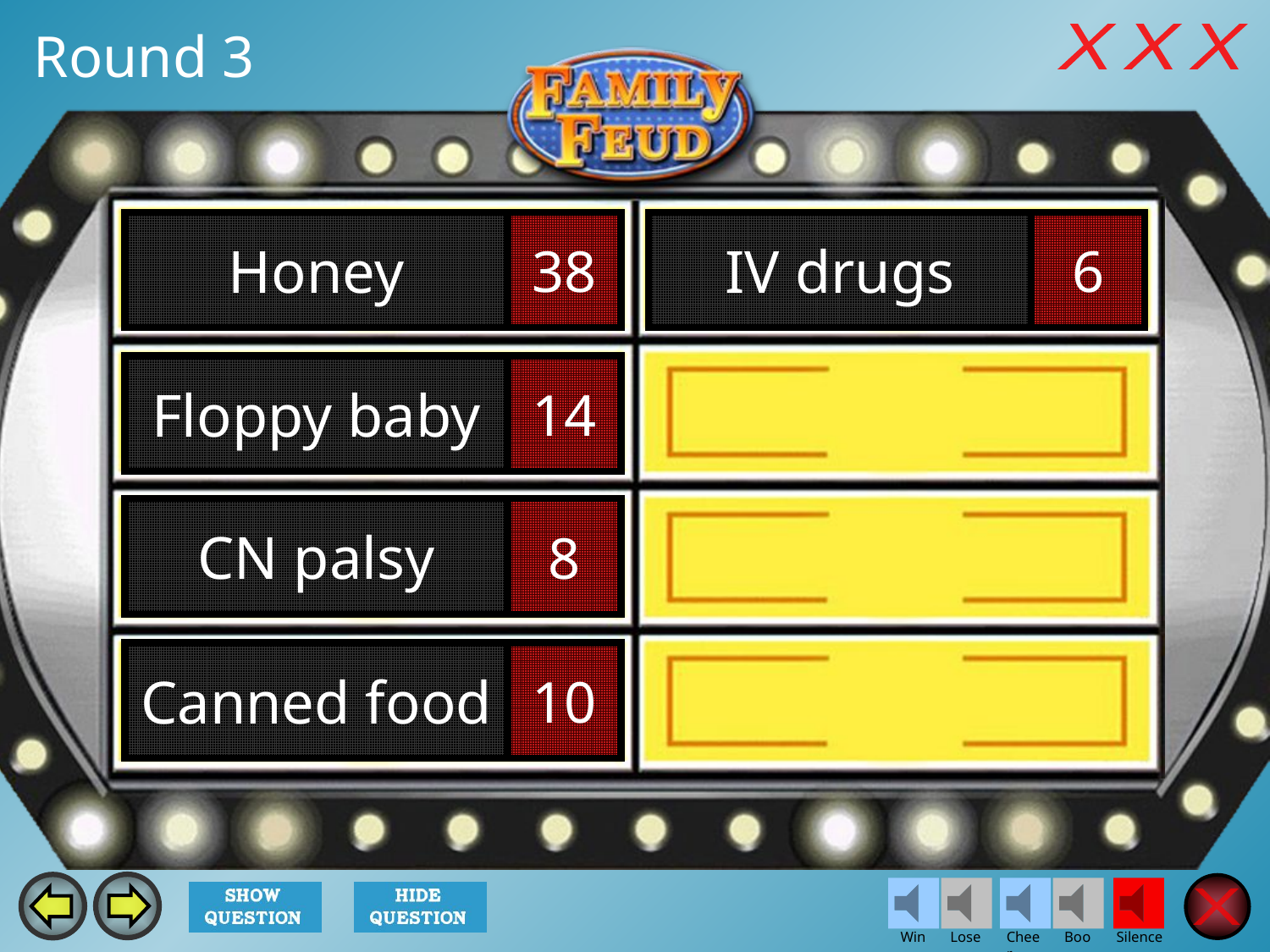

Five things suggestive of botulism
X
X
X
X
X
X
Round 3
X
X
X
Honey
38
IV drugs
6
Floppy baby
14
CN palsy
8
Canned food
10
Win
Lose
Cheer
Boo
Silence
X
X
X

## Slide 19
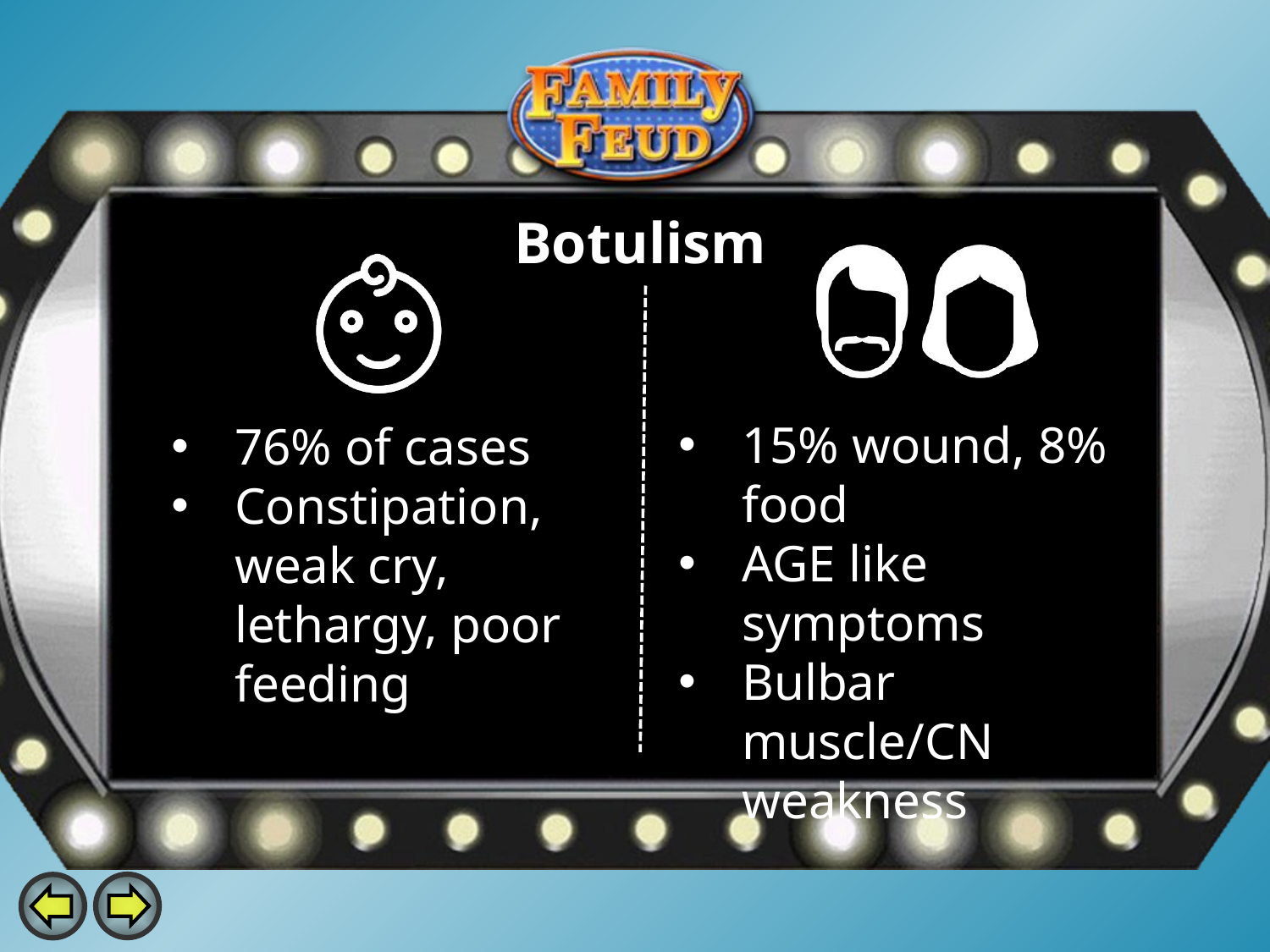

Botulism
15% wound, 8% food
AGE like symptoms
Bulbar muscle/CN weakness
76% of cases
Constipation, weak cry, lethargy, poor feeding

## Slide 20
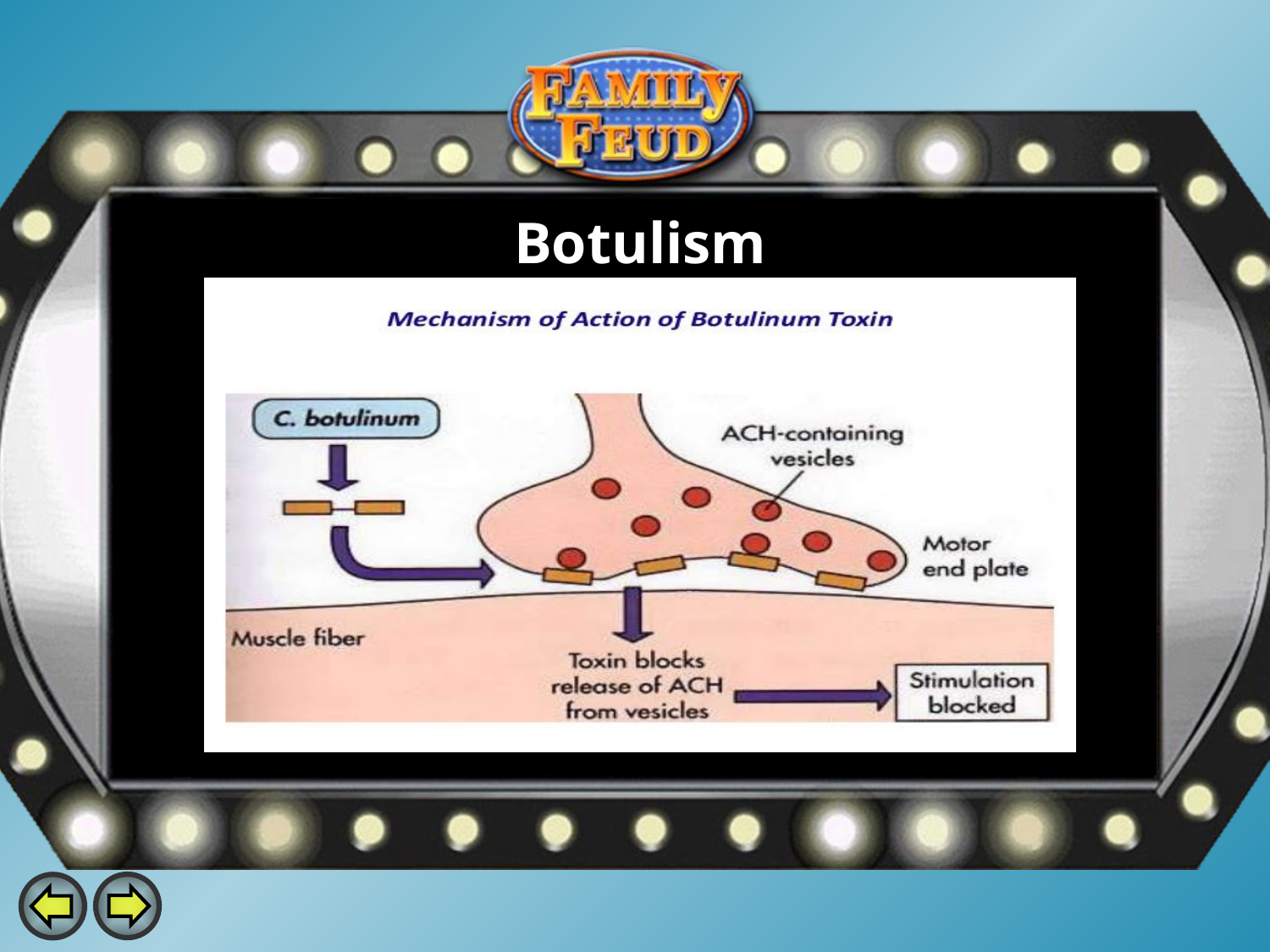

Botulism

## Slide 21
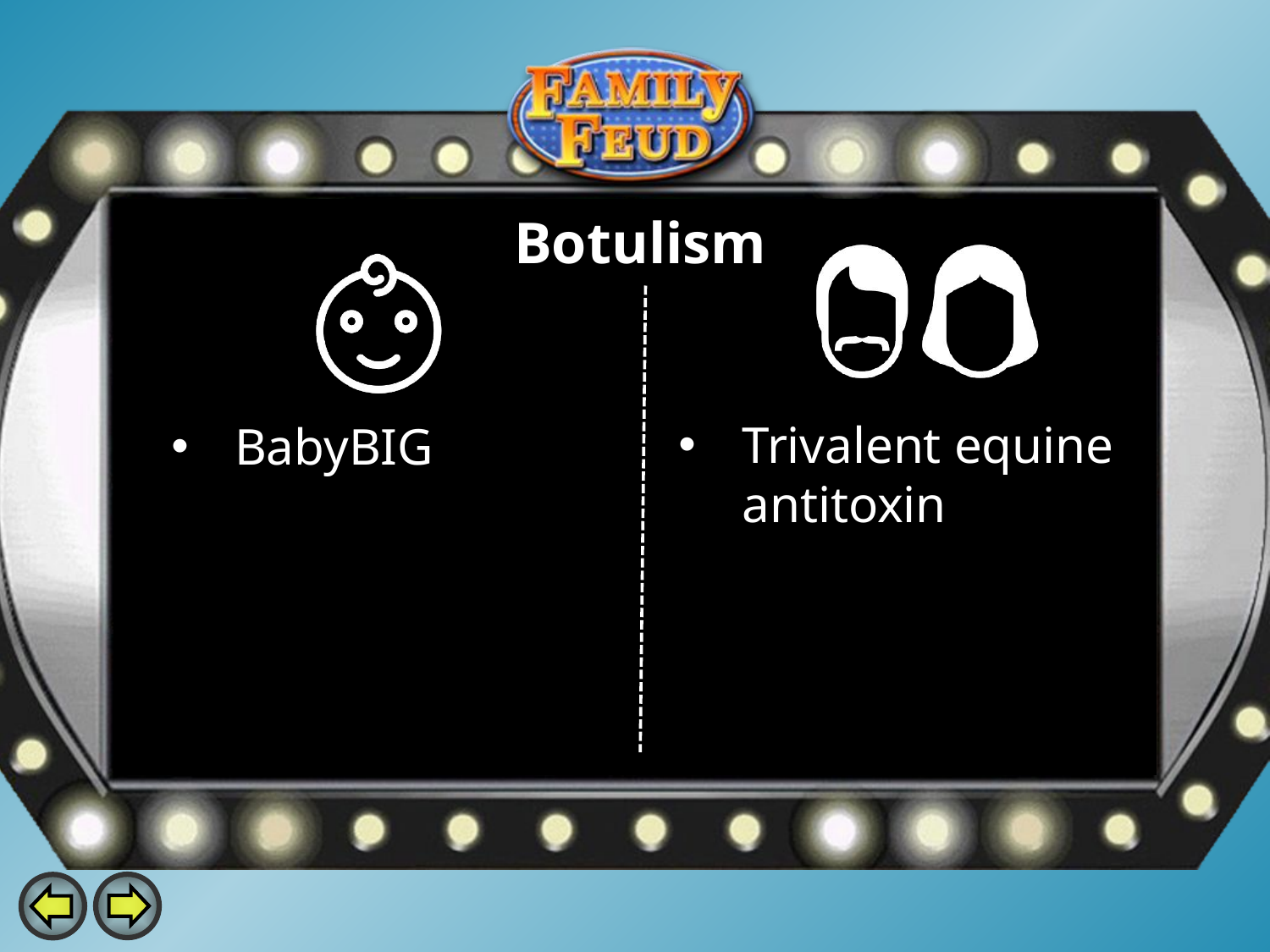

Botulism
Trivalent equine antitoxin
BabyBIG

## Slide 22
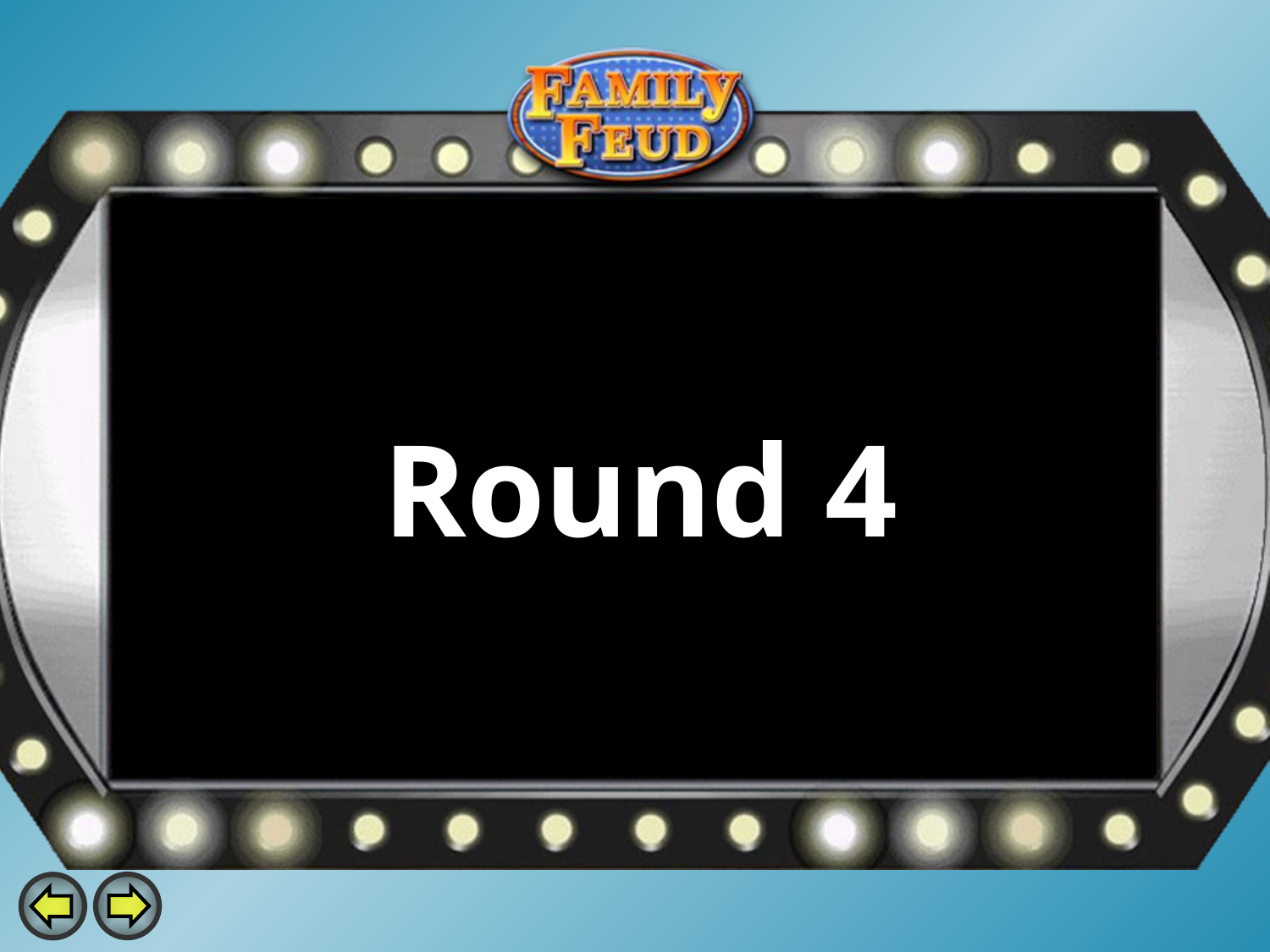

Round 4

## Slide 23
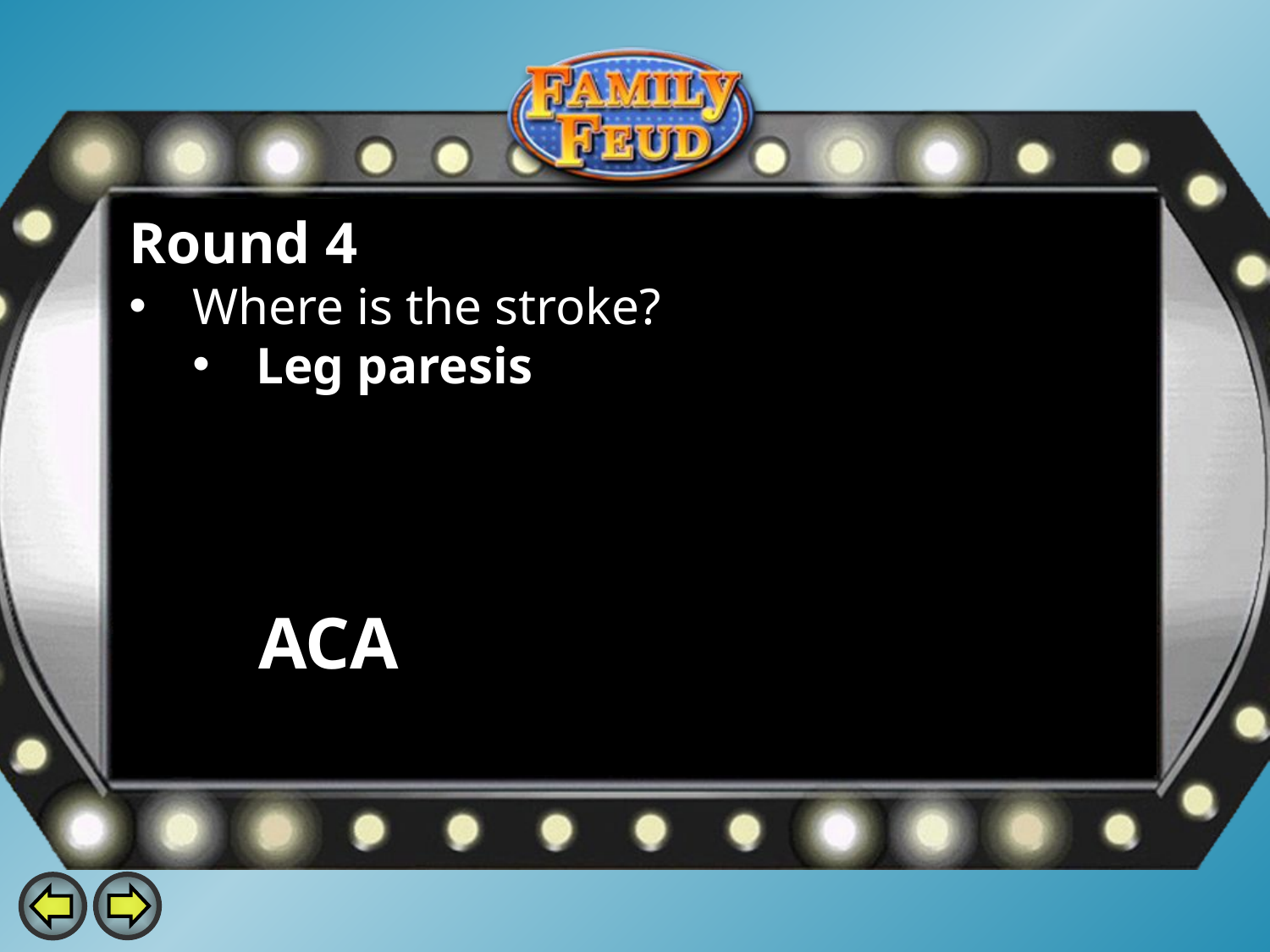

Round 4
Where is the stroke?
Leg paresis
ACA

## Slide 24
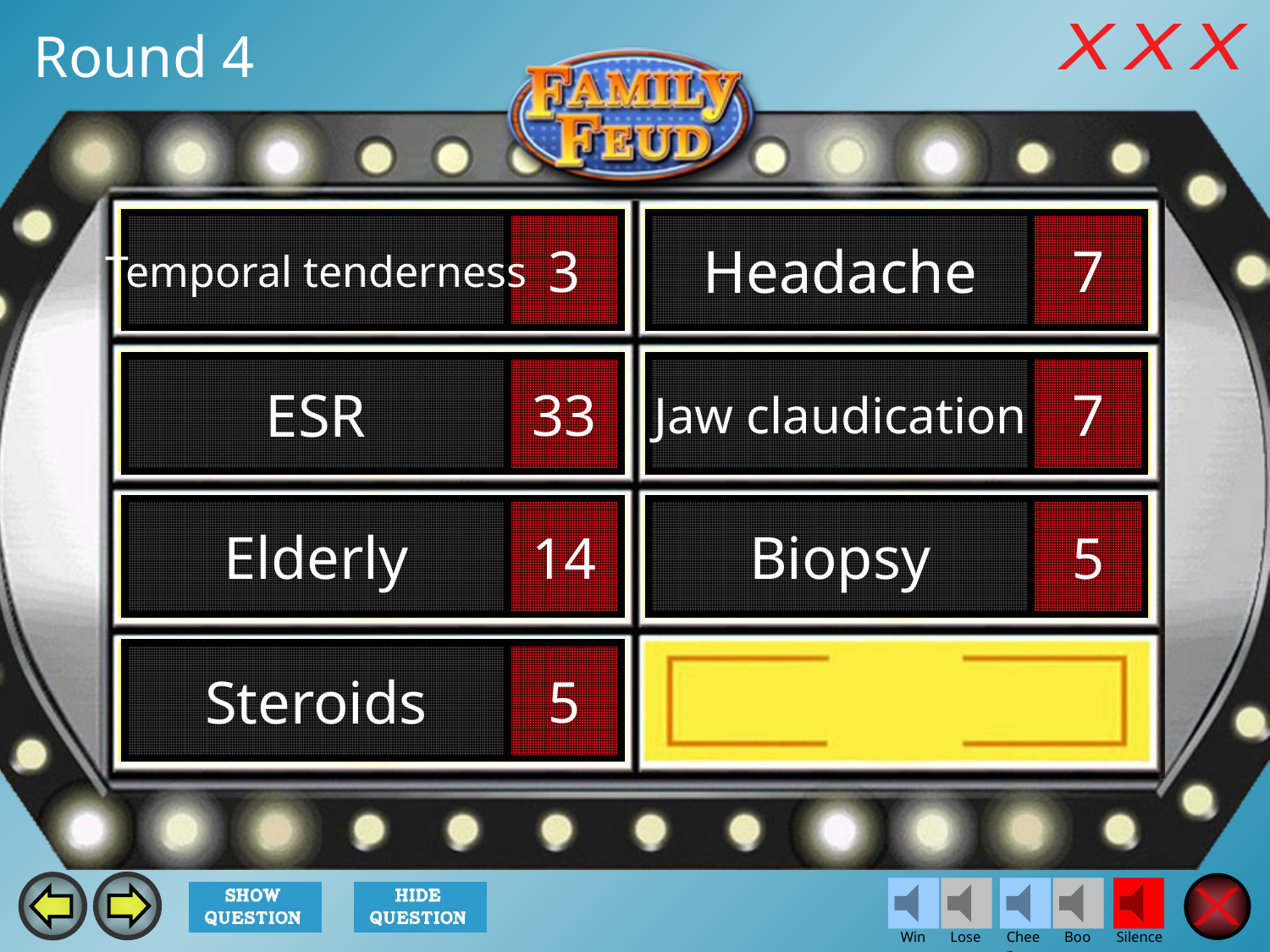

Name 5 things suggestive of giant cell arteritis
X
X
X
X
X
X
Round 4
X
X
X
Temporal tenderness
3
Headache
7
ESR
33
Jaw claudication
7
Elderly
14
Biopsy
5
Steroids
5
Win
Lose
Cheer
Boo
Silence
X
X
X

## Slide 25
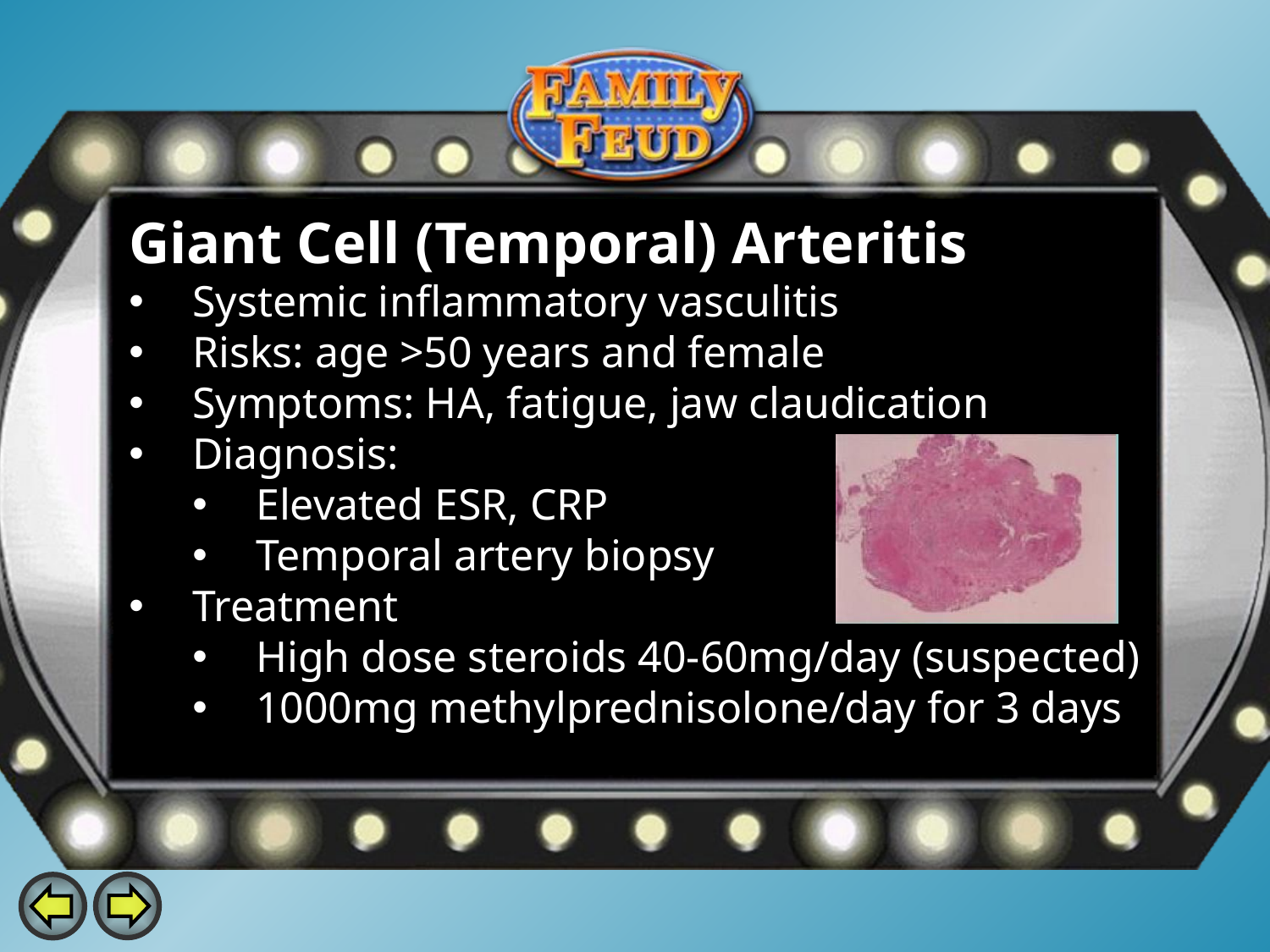

Giant Cell (Temporal) Arteritis
Systemic inflammatory vasculitis
Risks: age >50 years and female
Symptoms: HA, fatigue, jaw claudication
Diagnosis:
Elevated ESR, CRP
Temporal artery biopsy
Treatment
High dose steroids 40-60mg/day (suspected)
1000mg methylprednisolone/day for 3 days

## Slide 26
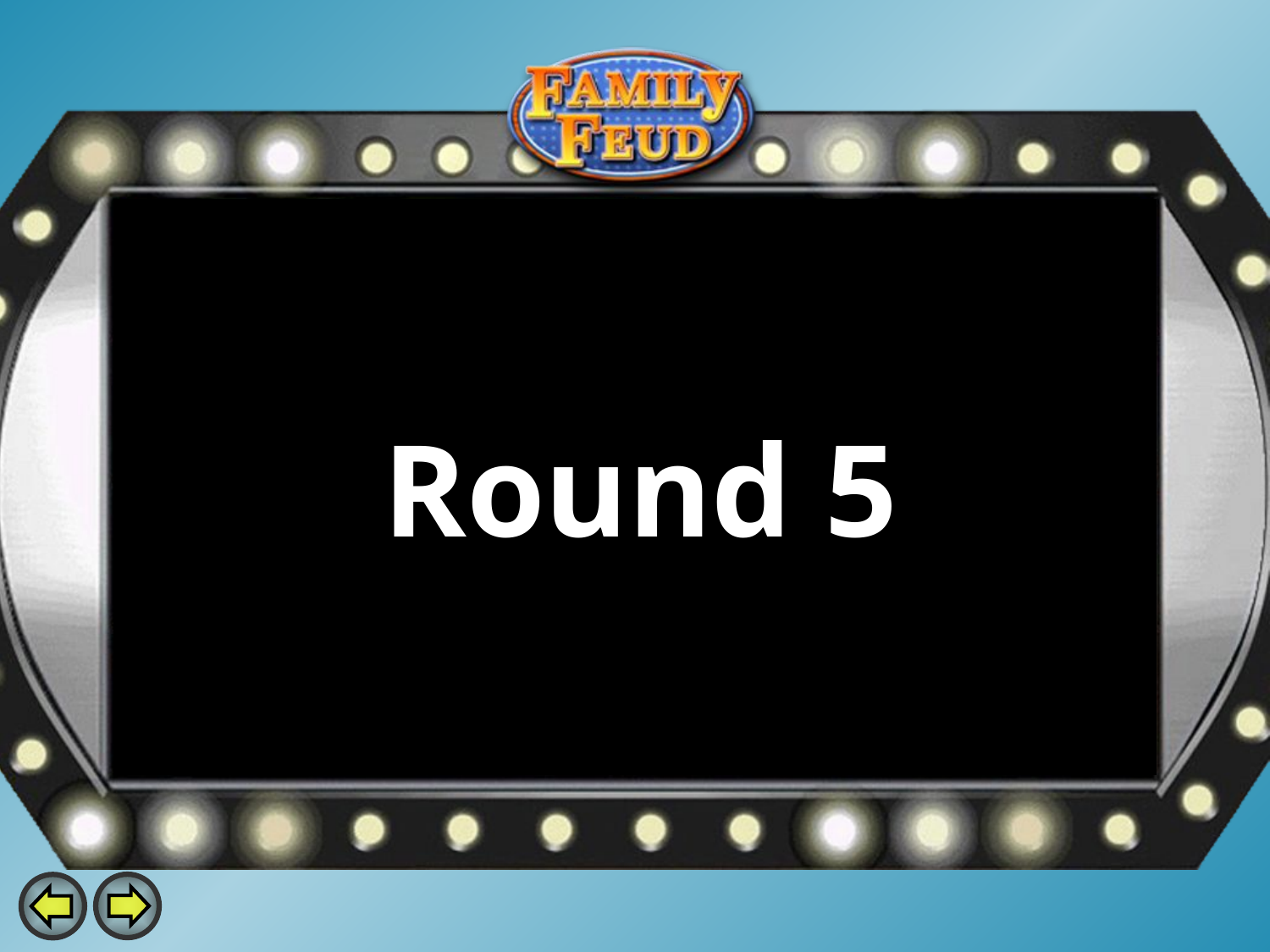

Round 5

## Slide 27
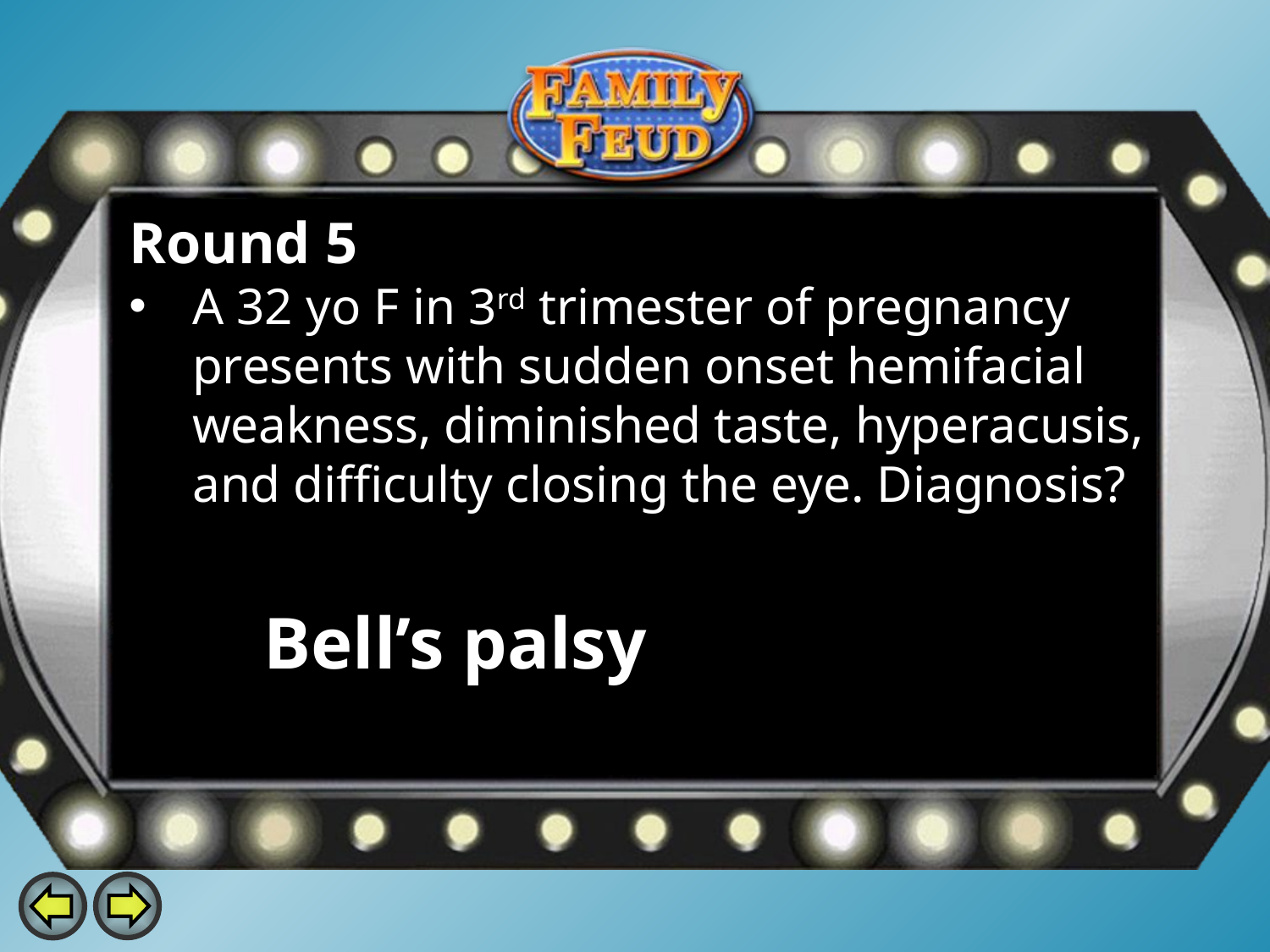

Round 5
A 32 yo F in 3rd trimester of pregnancy presents with sudden onset hemifacial weakness, diminished taste, hyperacusis, and difficulty closing the eye. Diagnosis?
Bell’s palsy

## Slide 28
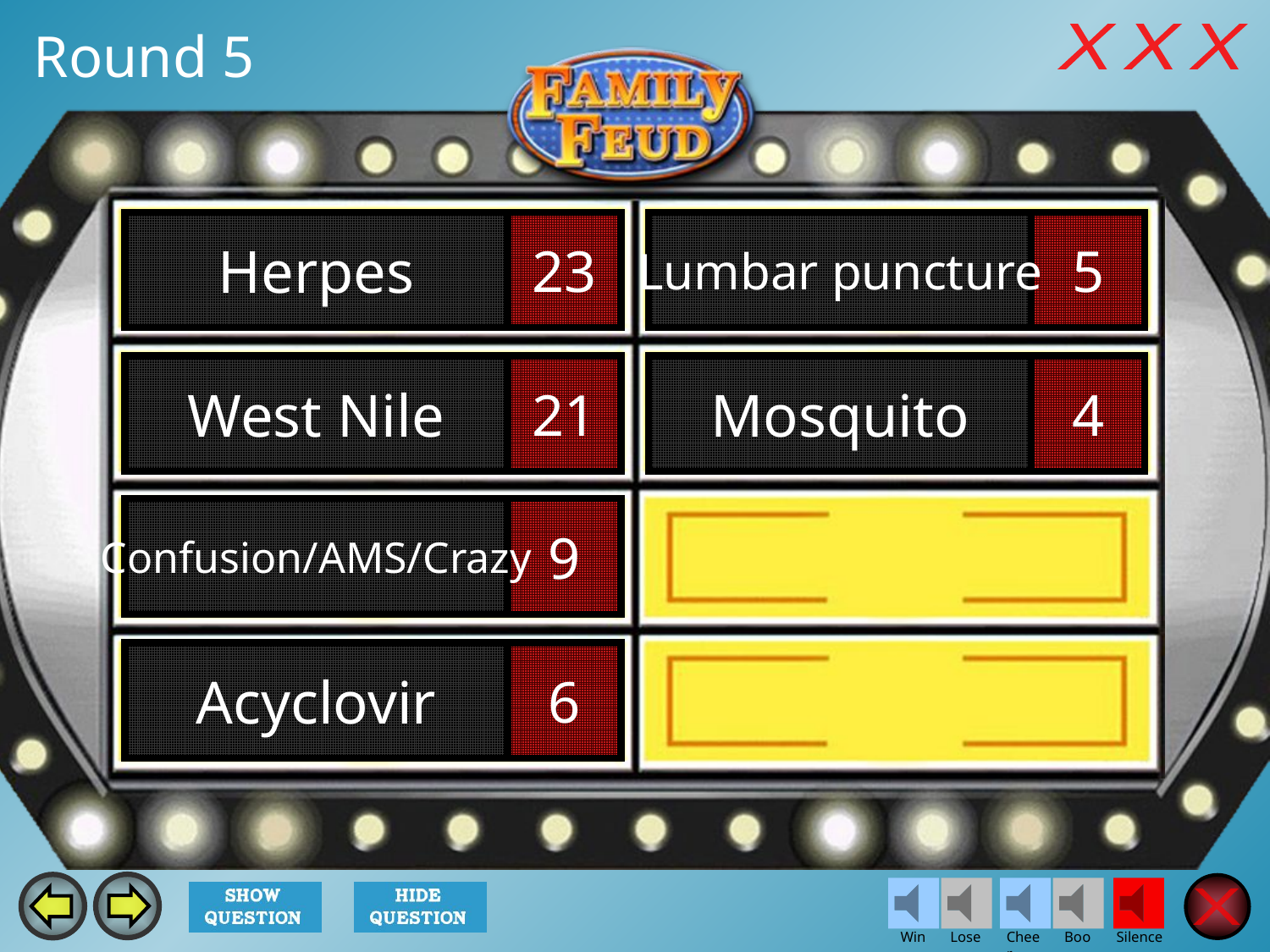

Viral encephalitis
X
X
X
X
X
X
Round 5
X
X
X
Herpes
23
Lumbar puncture
5
West Nile
21
Mosquito
4
Confusion/AMS/Crazy
9
Acyclovir
6
Win
Lose
Cheer
Boo
Silence
X
X
X

## Slide 29
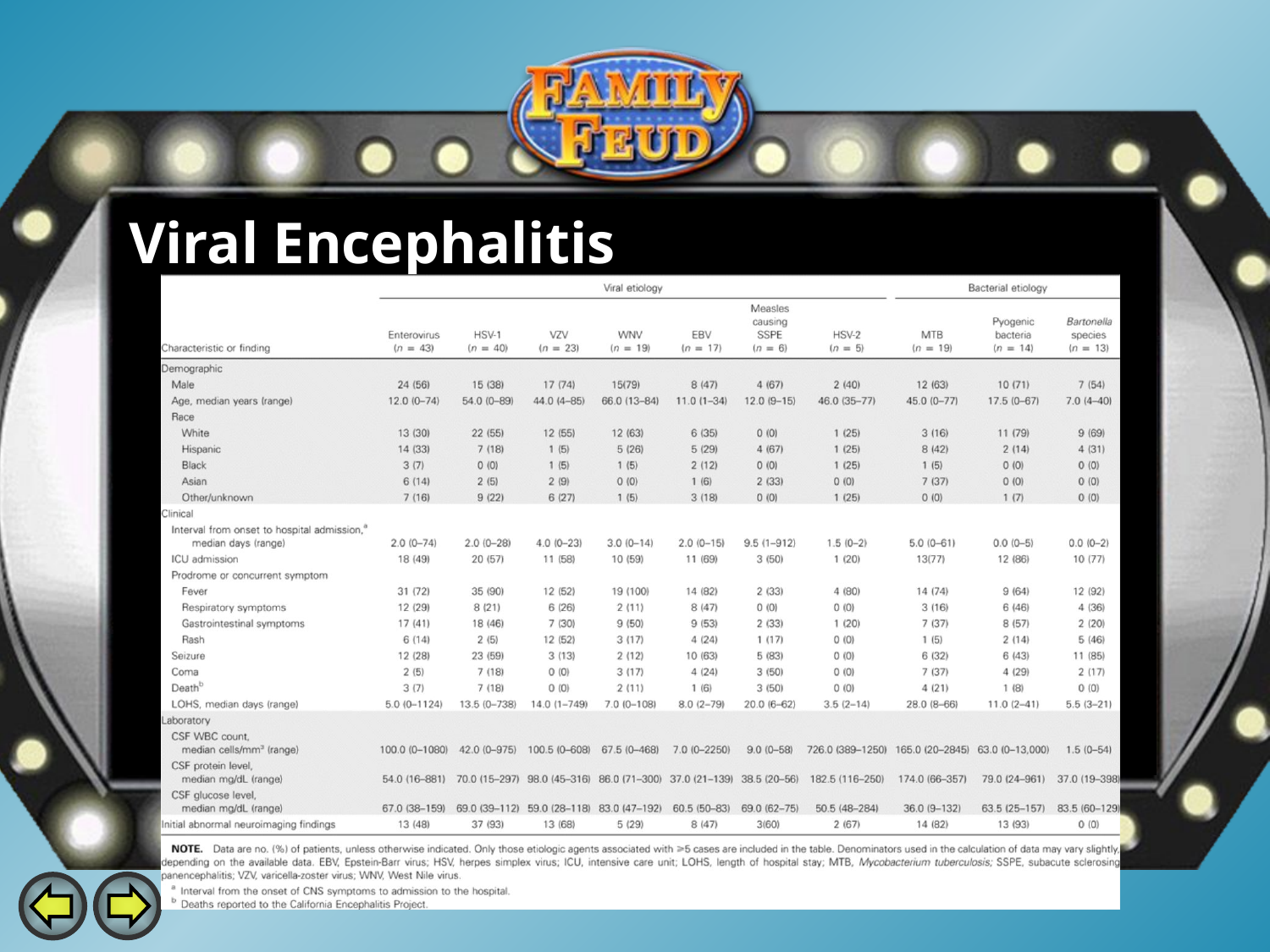

Viral Encephalitis

## Slide 30
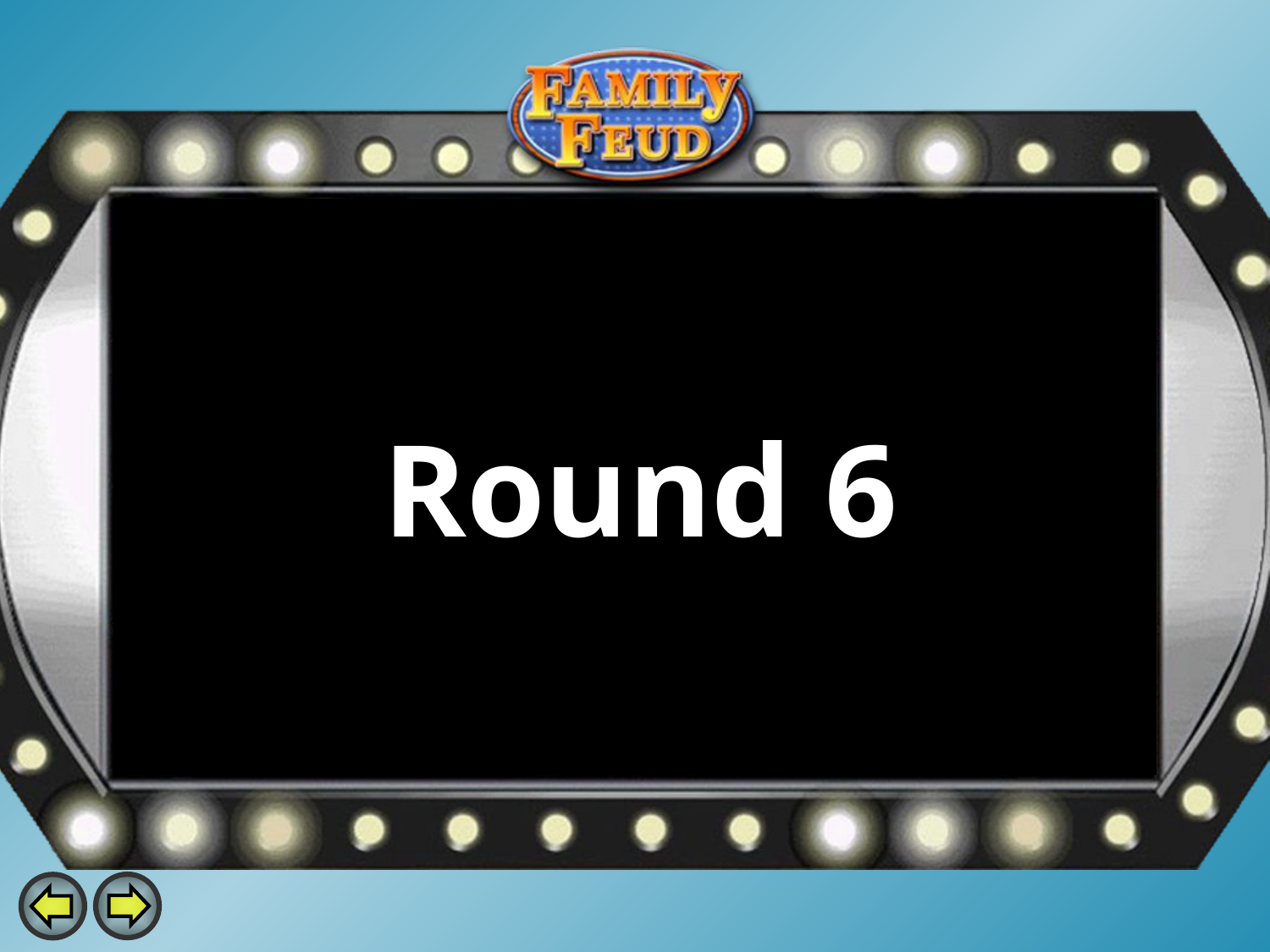

Round 6

## Slide 31
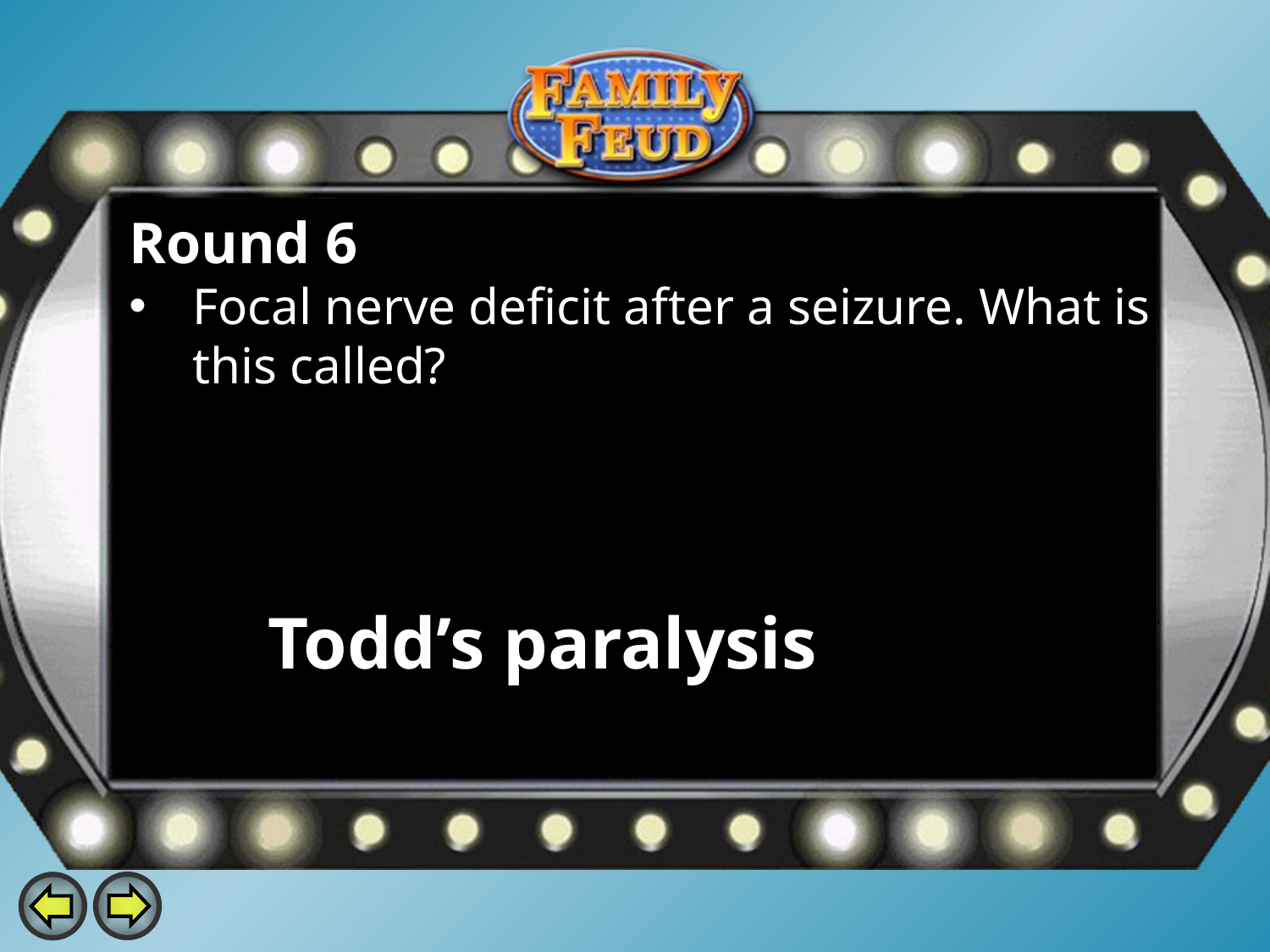

Round 6
Focal nerve deficit after a seizure. What is this called?
Todd’s paralysis

## Slide 32
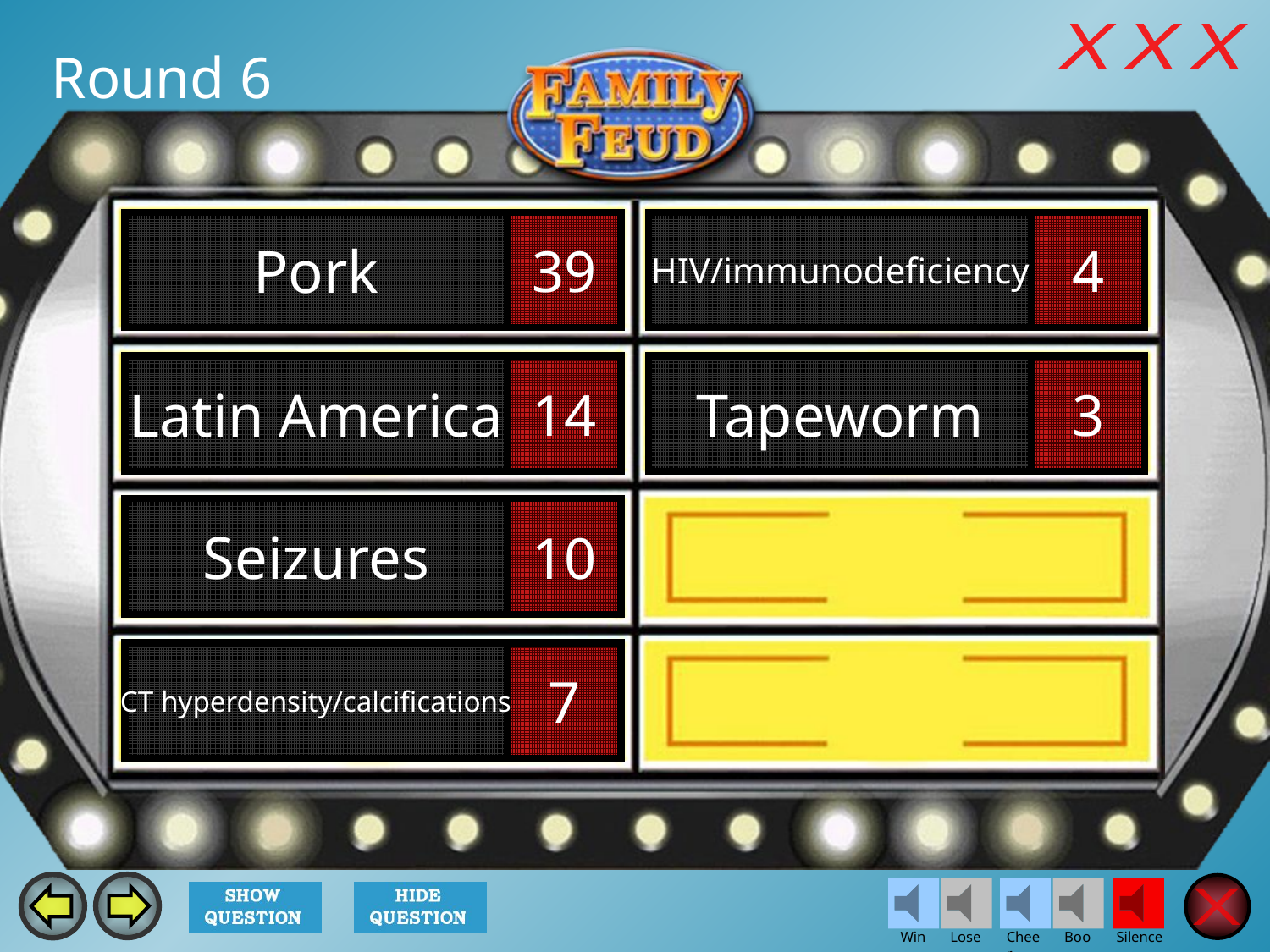

Neurocysticercosis
X
X
X
X
X
X
X
X
X
Round 6
Pork
39
HIV/immunodeficiency
4
Latin America
14
Tapeworm
3
Seizures
10
CT hyperdensity/calcifications
7
Win
Lose
Cheer
Boo
Silence
X
X
X

## Slide 33
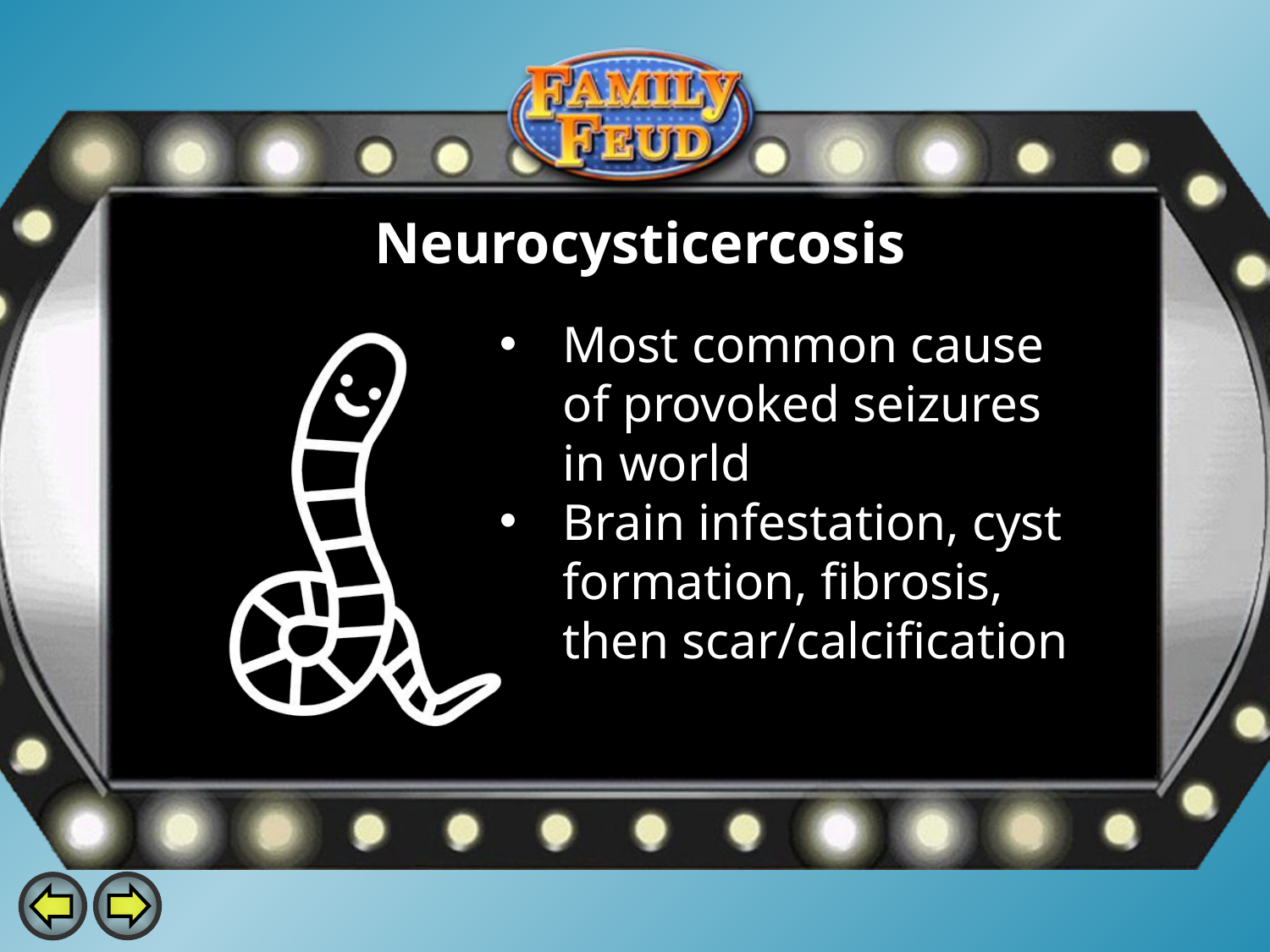

Neurocysticercosis
Most common cause of provoked seizures in world
Brain infestation, cyst formation, fibrosis, then scar/calcification

## Slide 34
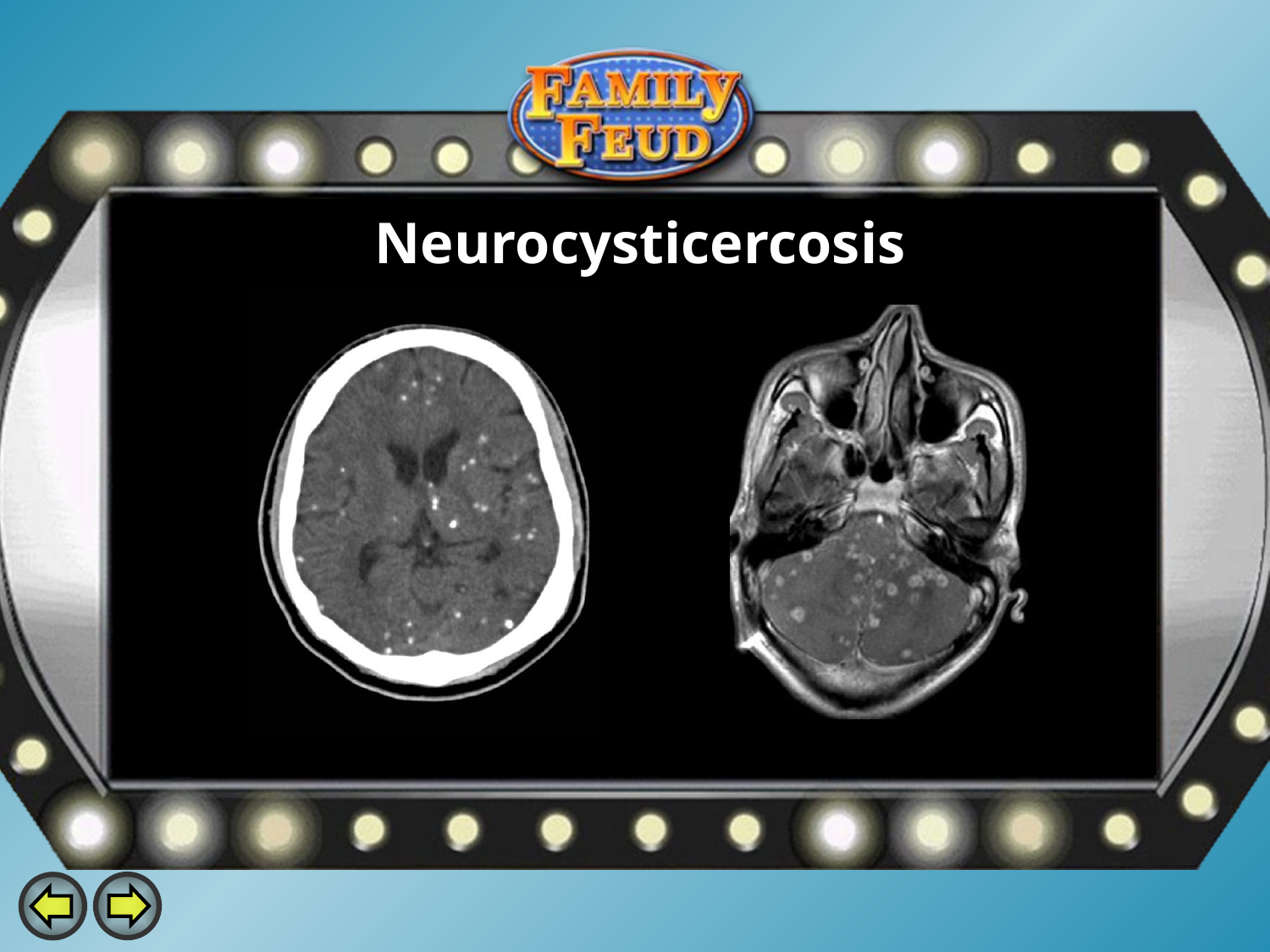

Neurocysticercosis

## Slide 35
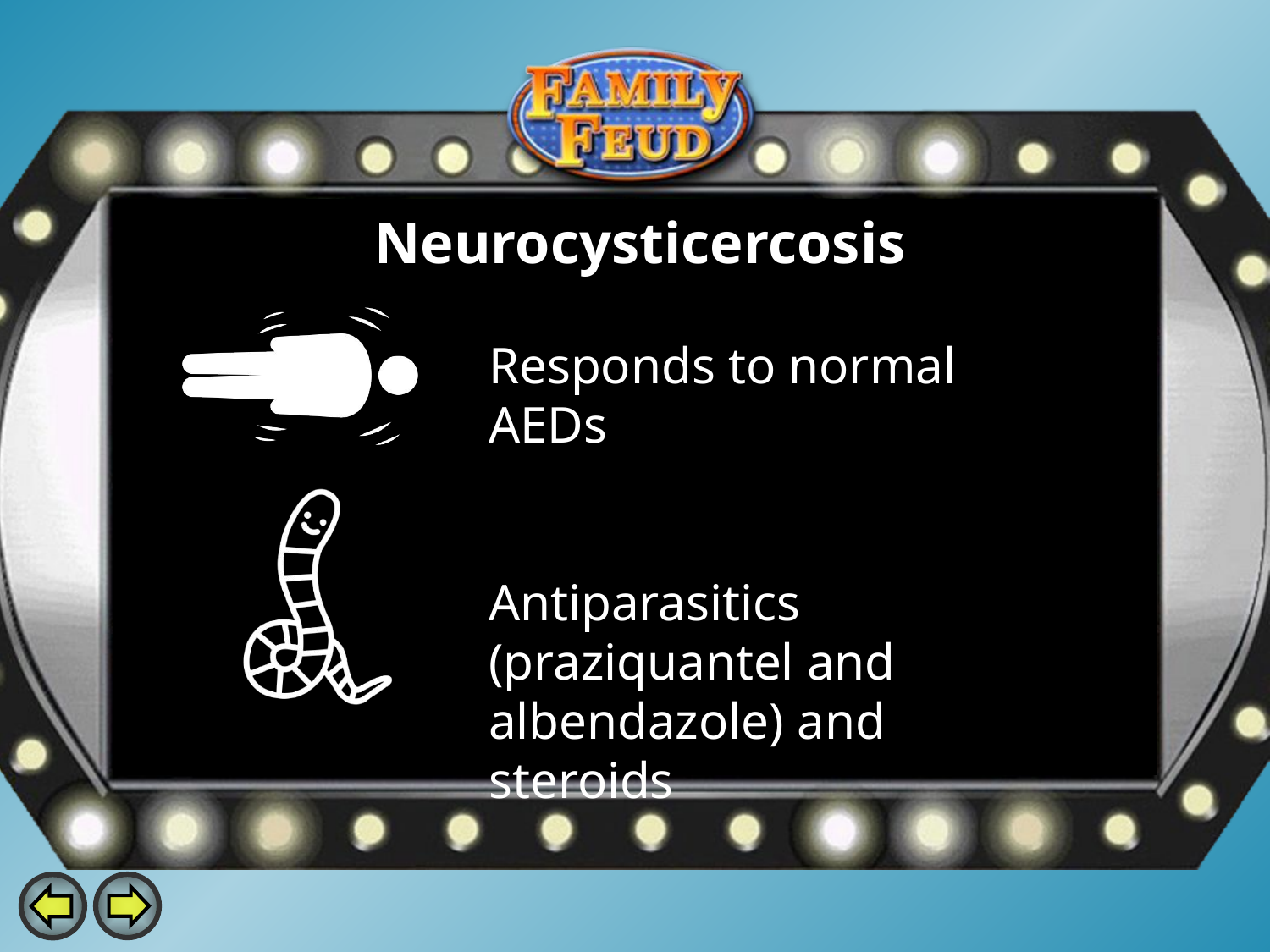

Neurocysticercosis
Responds to normal AEDs
Antiparasitics (praziquantel and albendazole) and steroids

## Slide 36
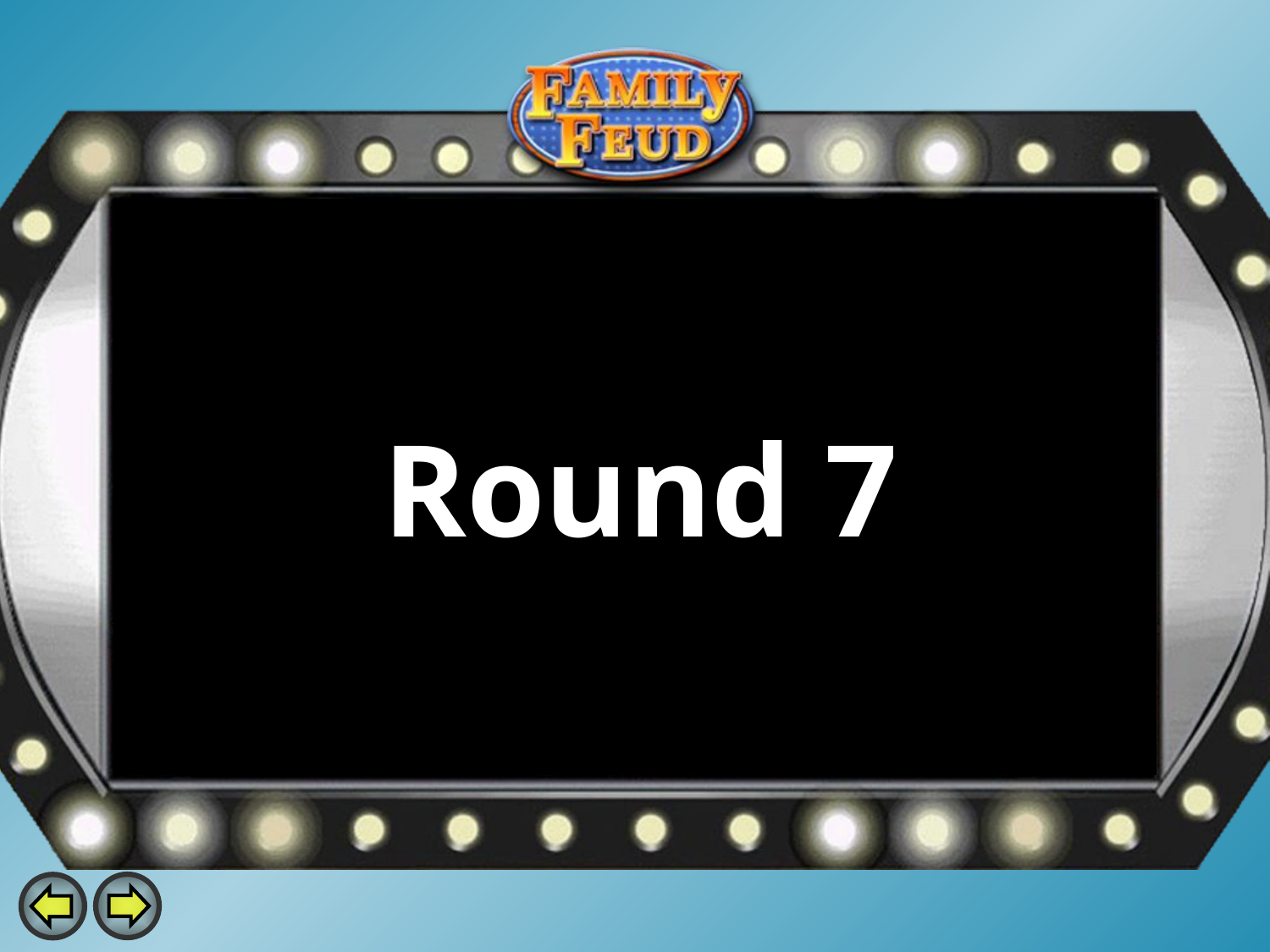

Round 7

## Slide 37
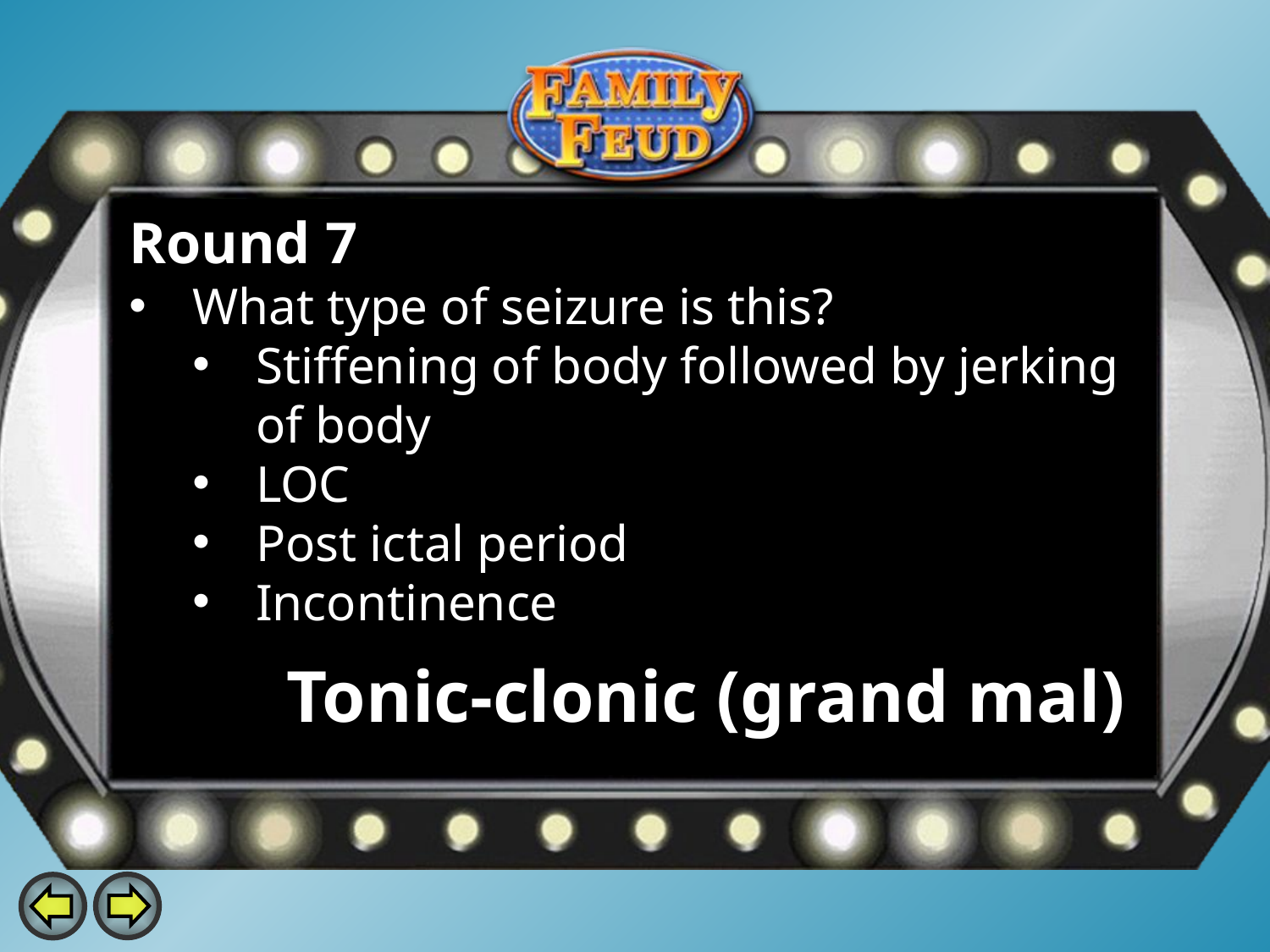

Round 7
What type of seizure is this?
Stiffening of body followed by jerking of body
LOC
Post ictal period
Incontinence
Tonic-clonic (grand mal)

## Slide 38
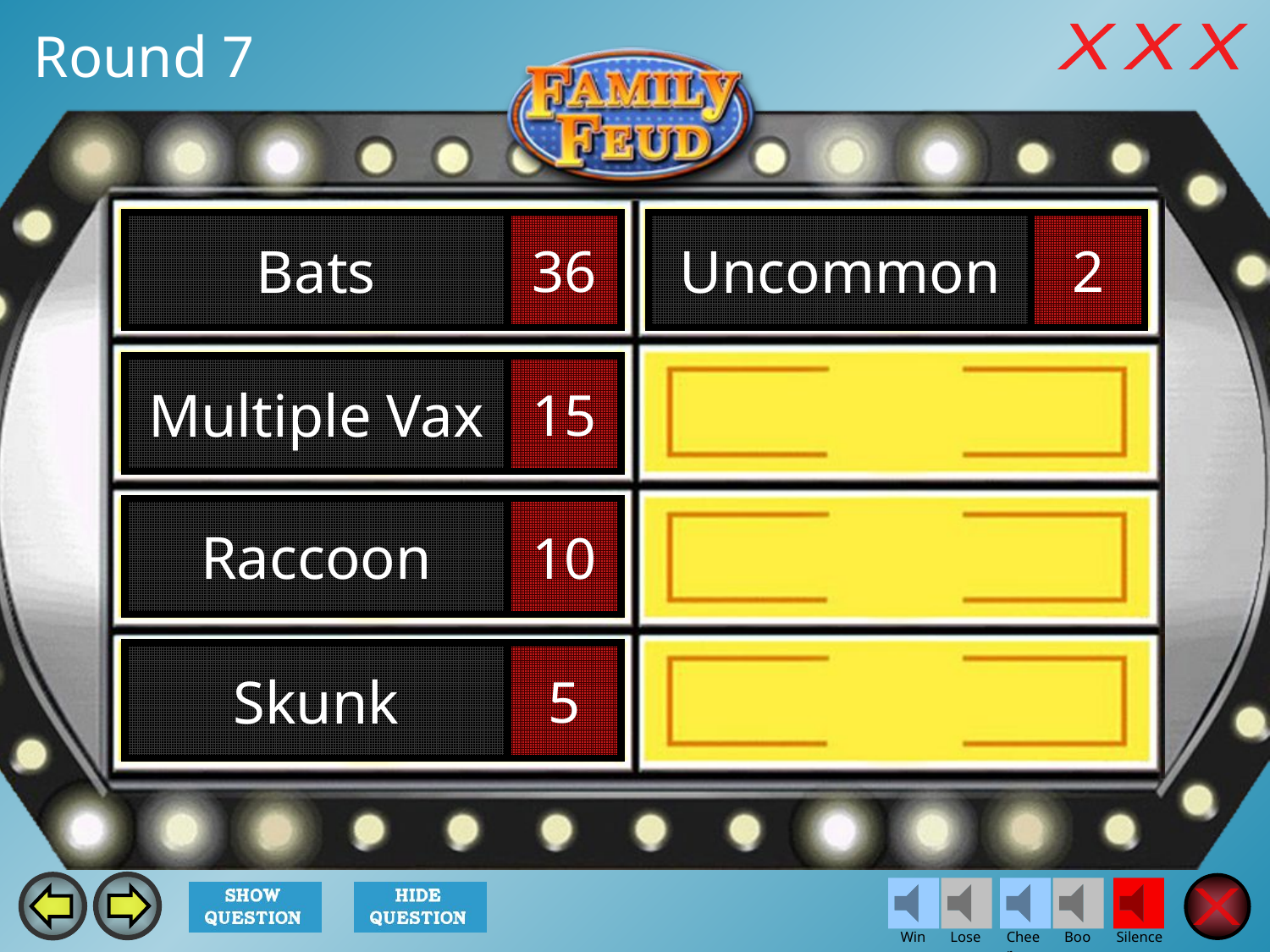

Rabies
X
X
X
X
X
X
Round 7
X
X
X
Bats
36
Uncommon
2
Multiple Vax
15
Raccoon
10
Skunk
5
Win
Lose
Cheer
Boo
Silence
X
X
X

## Slide 39
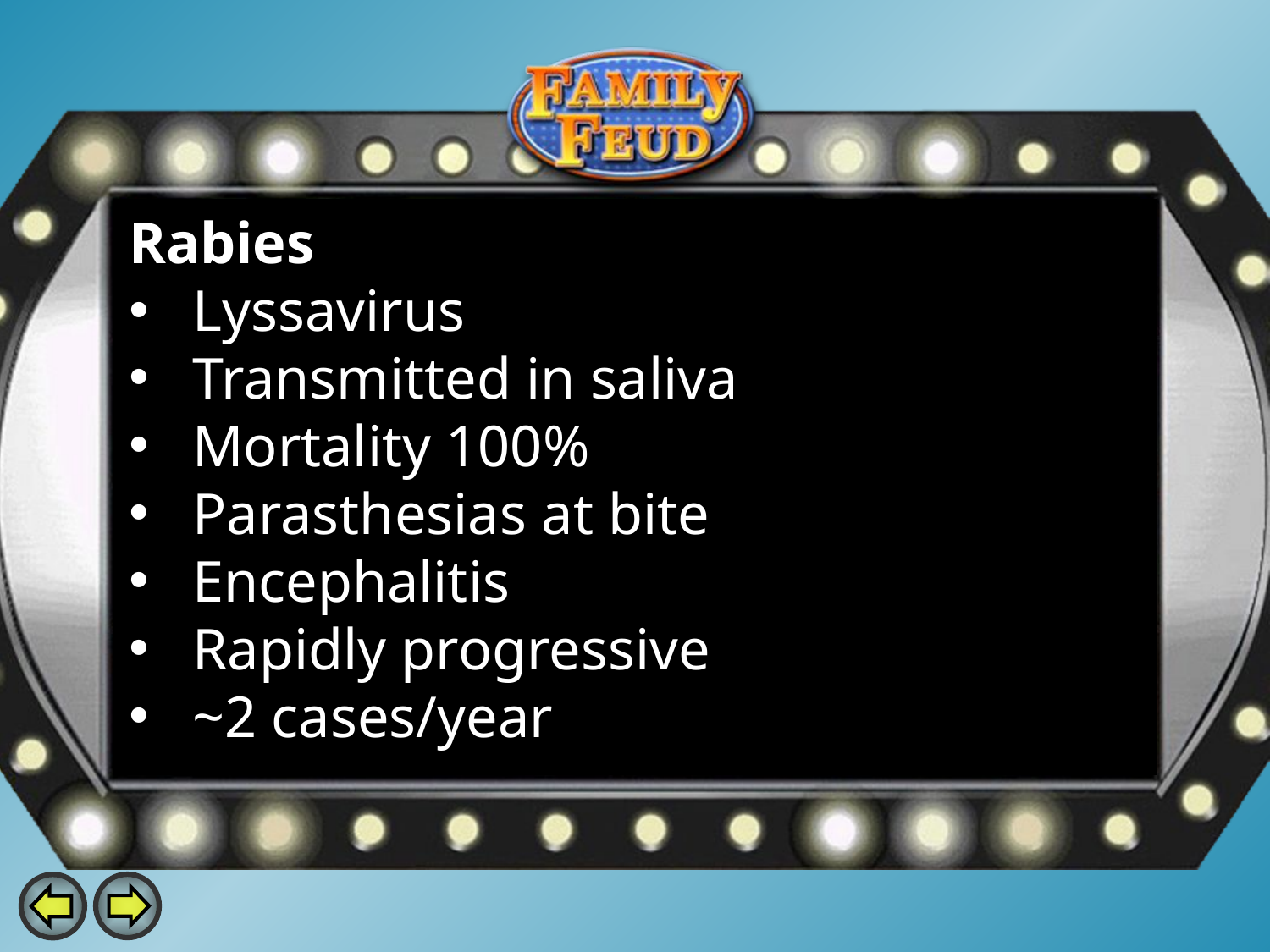

Rabies
Lyssavirus
Transmitted in saliva
Mortality 100%
Parasthesias at bite
Encephalitis
Rapidly progressive
~2 cases/year

## Slide 40
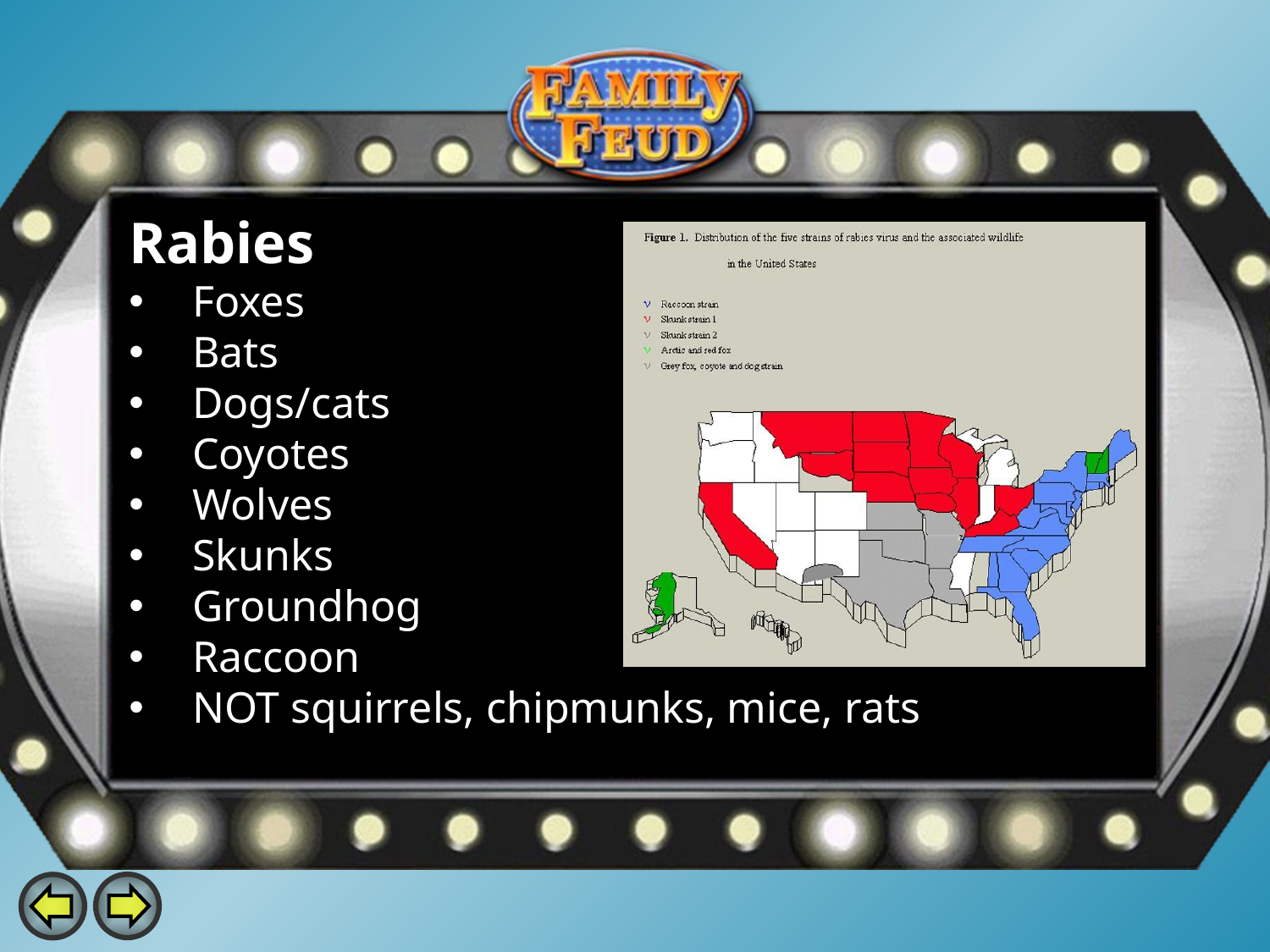

Rabies
Foxes
Bats
Dogs/cats
Coyotes
Wolves
Skunks
Groundhog
Raccoon
NOT squirrels, chipmunks, mice, rats

## Slide 41
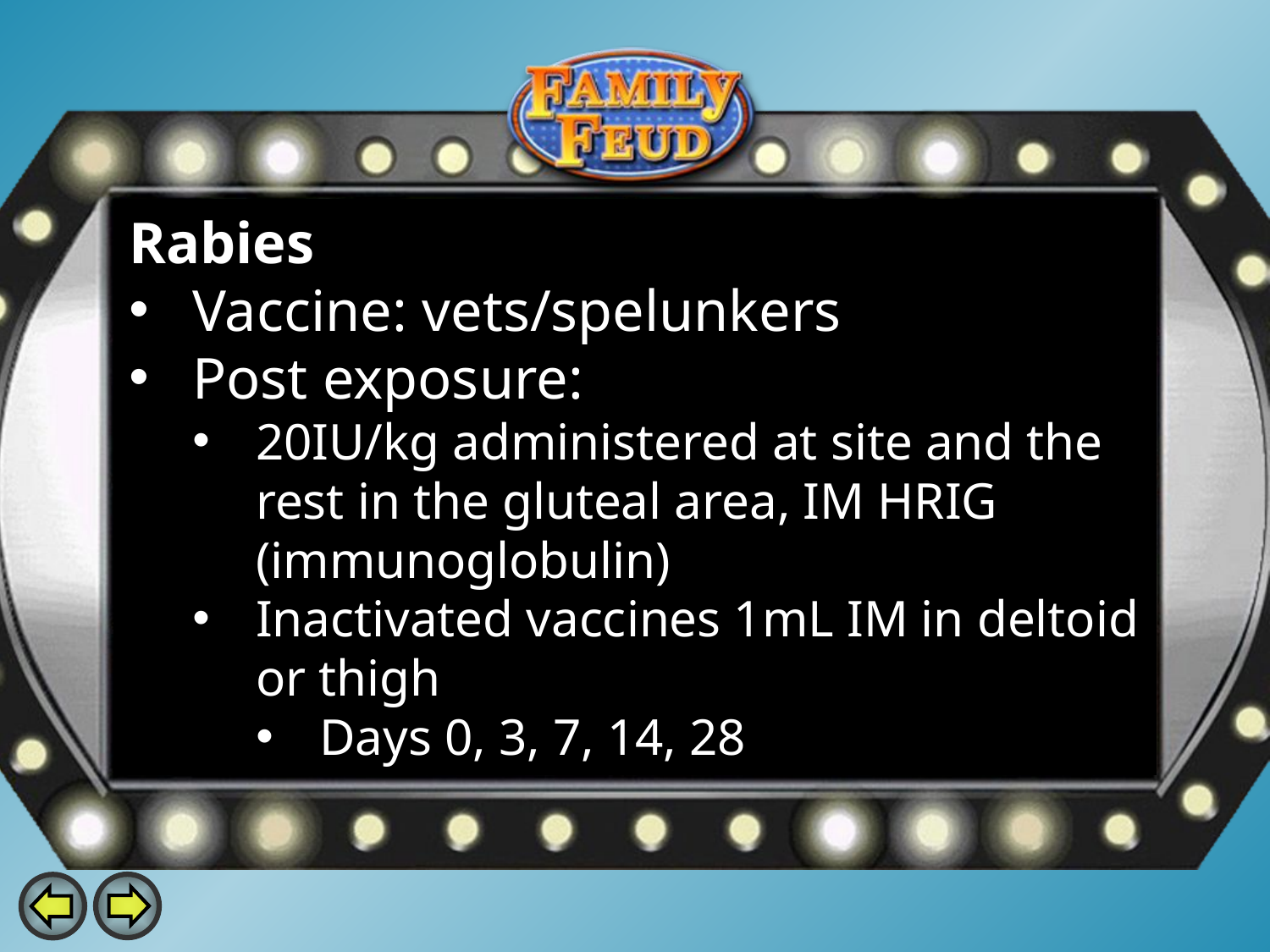

Rabies
Vaccine: vets/spelunkers
Post exposure:
20IU/kg administered at site and the rest in the gluteal area, IM HRIG (immunoglobulin)
Inactivated vaccines 1mL IM in deltoid or thigh
Days 0, 3, 7, 14, 28

## Slide 42
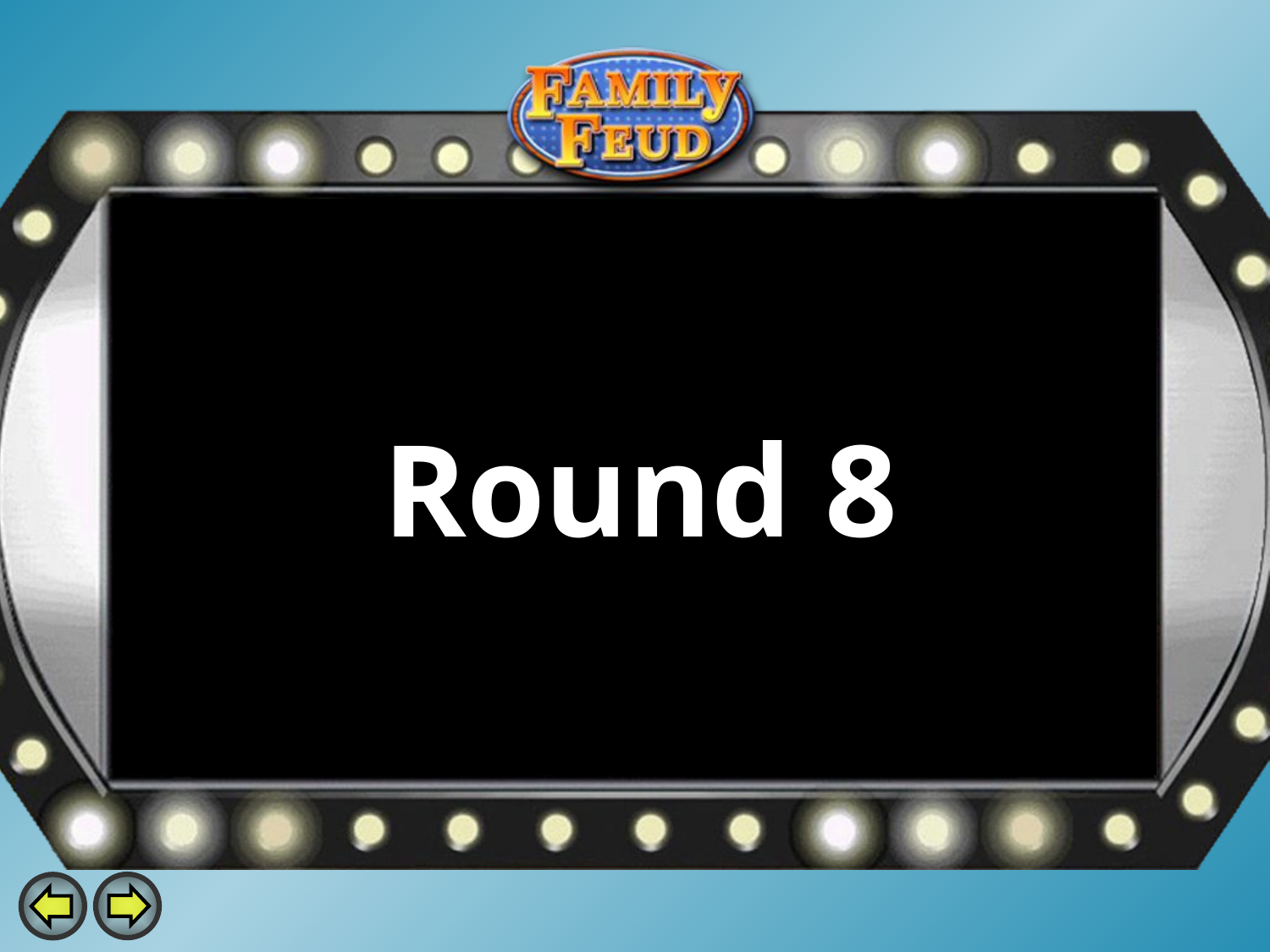

Round 8

## Slide 43
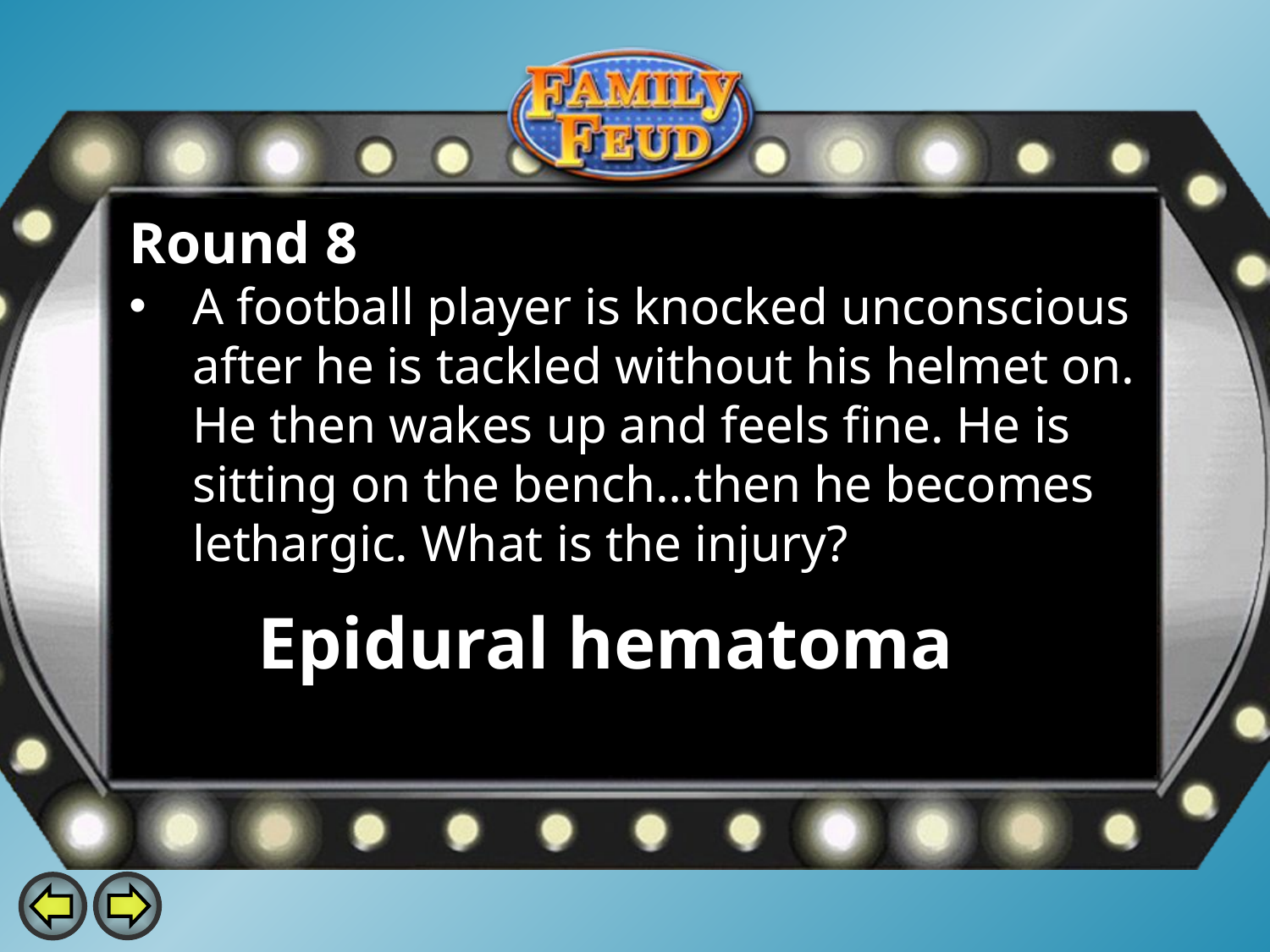

Round 8
A football player is knocked unconscious after he is tackled without his helmet on. He then wakes up and feels fine. He is sitting on the bench…then he becomes lethargic. What is the injury?
Epidural hematoma

## Slide 44
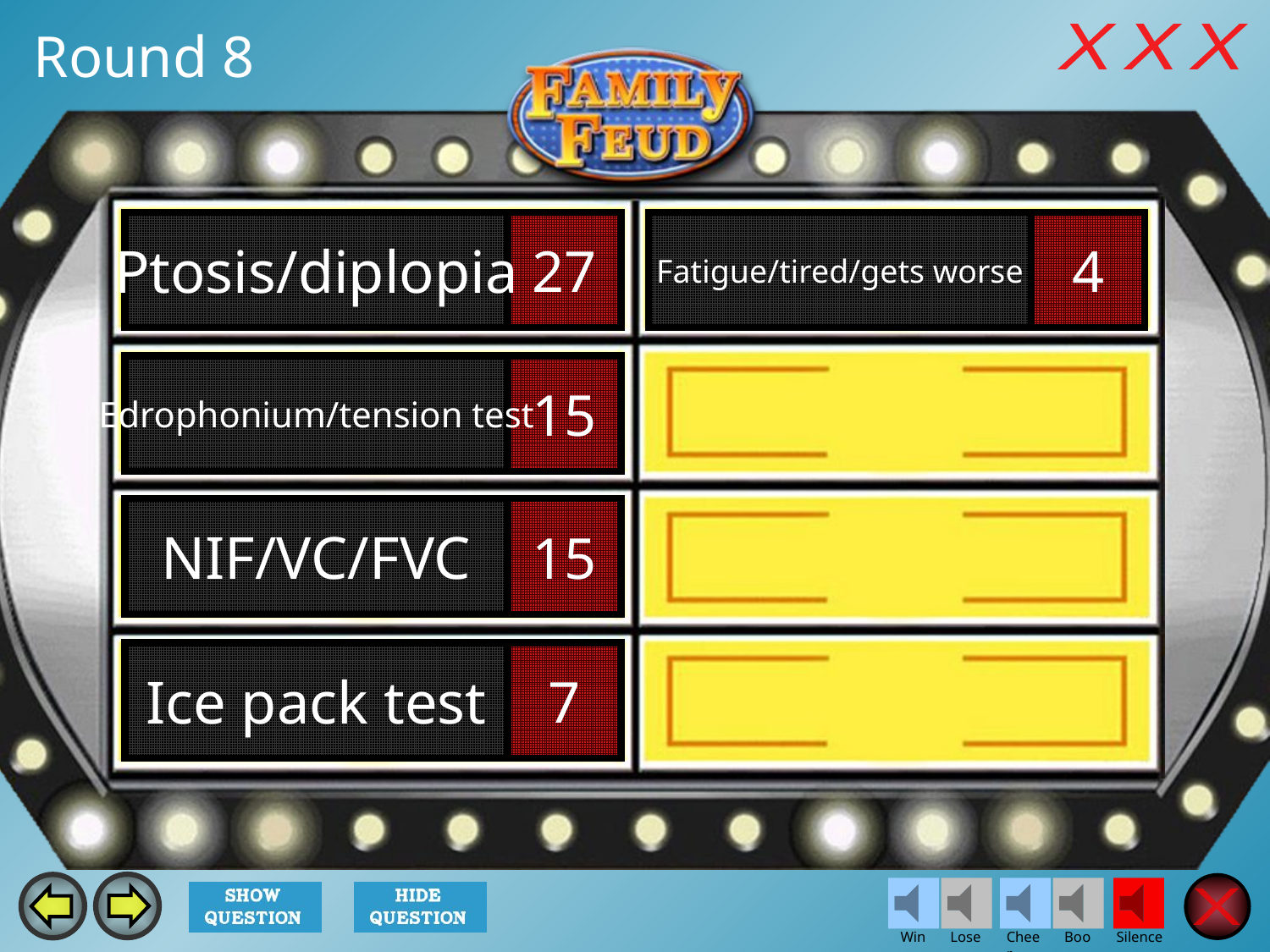

Myasthenia gravis
X
X
X
X
X
X
Round 8
X
X
X
Ptosis/diplopia
27
Fatigue/tired/gets worse
4
Edrophonium/tension test
15
NIF/VC/FVC
15
Ice pack test
7
Win
Lose
Cheer
Boo
Silence
X
X
X

## Slide 45
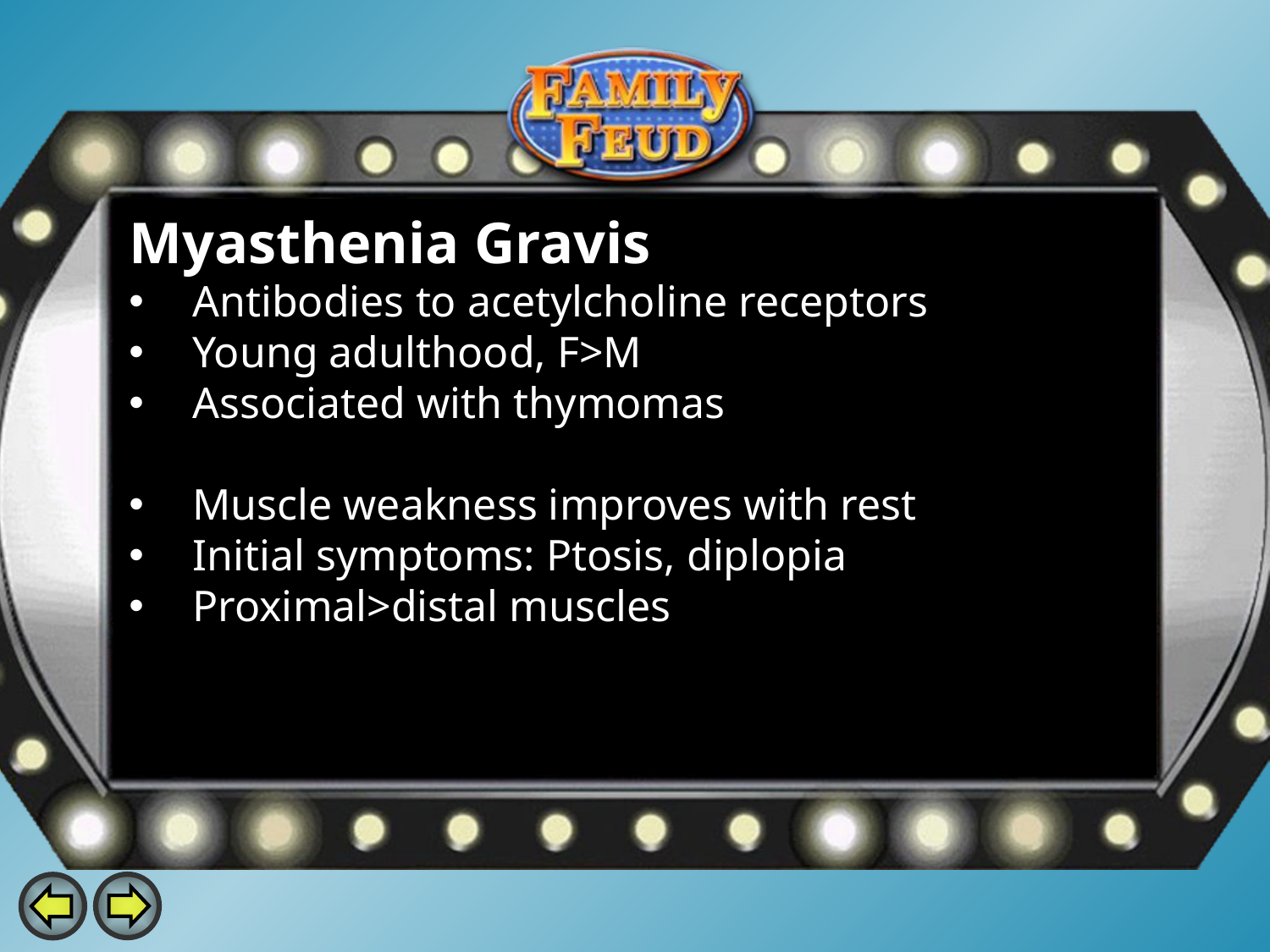

Myasthenia Gravis
Antibodies to acetylcholine receptors
Young adulthood, F>M
Associated with thymomas
Muscle weakness improves with rest
Initial symptoms: Ptosis, diplopia
Proximal>distal muscles

## Slide 46
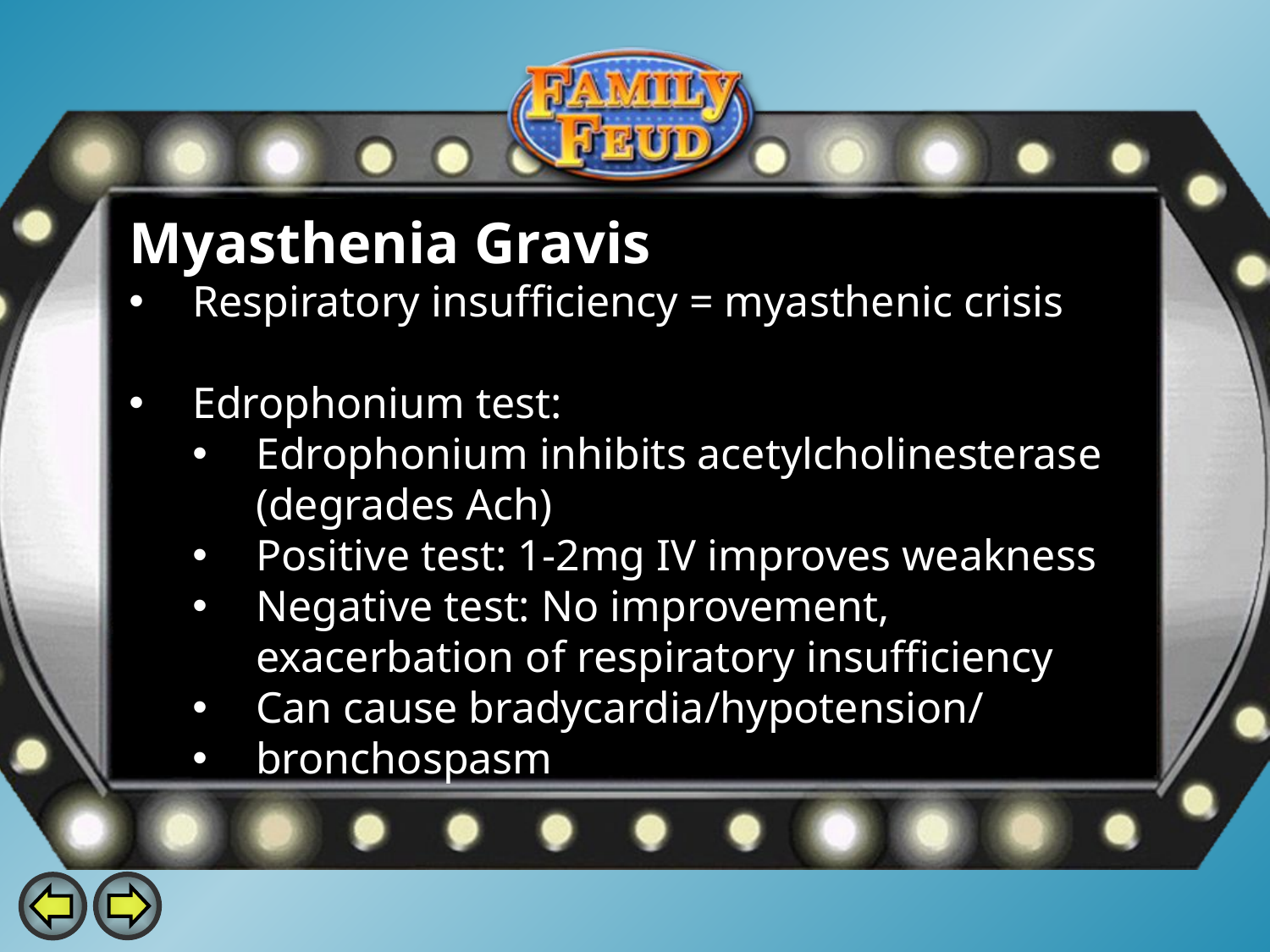

Myasthenia Gravis
Respiratory insufficiency = myasthenic crisis
Edrophonium test:
Edrophonium inhibits acetylcholinesterase (degrades Ach)
Positive test: 1-2mg IV improves weakness
Negative test: No improvement, exacerbation of respiratory insufficiency
Can cause bradycardia/hypotension/
bronchospasm

## Slide 47
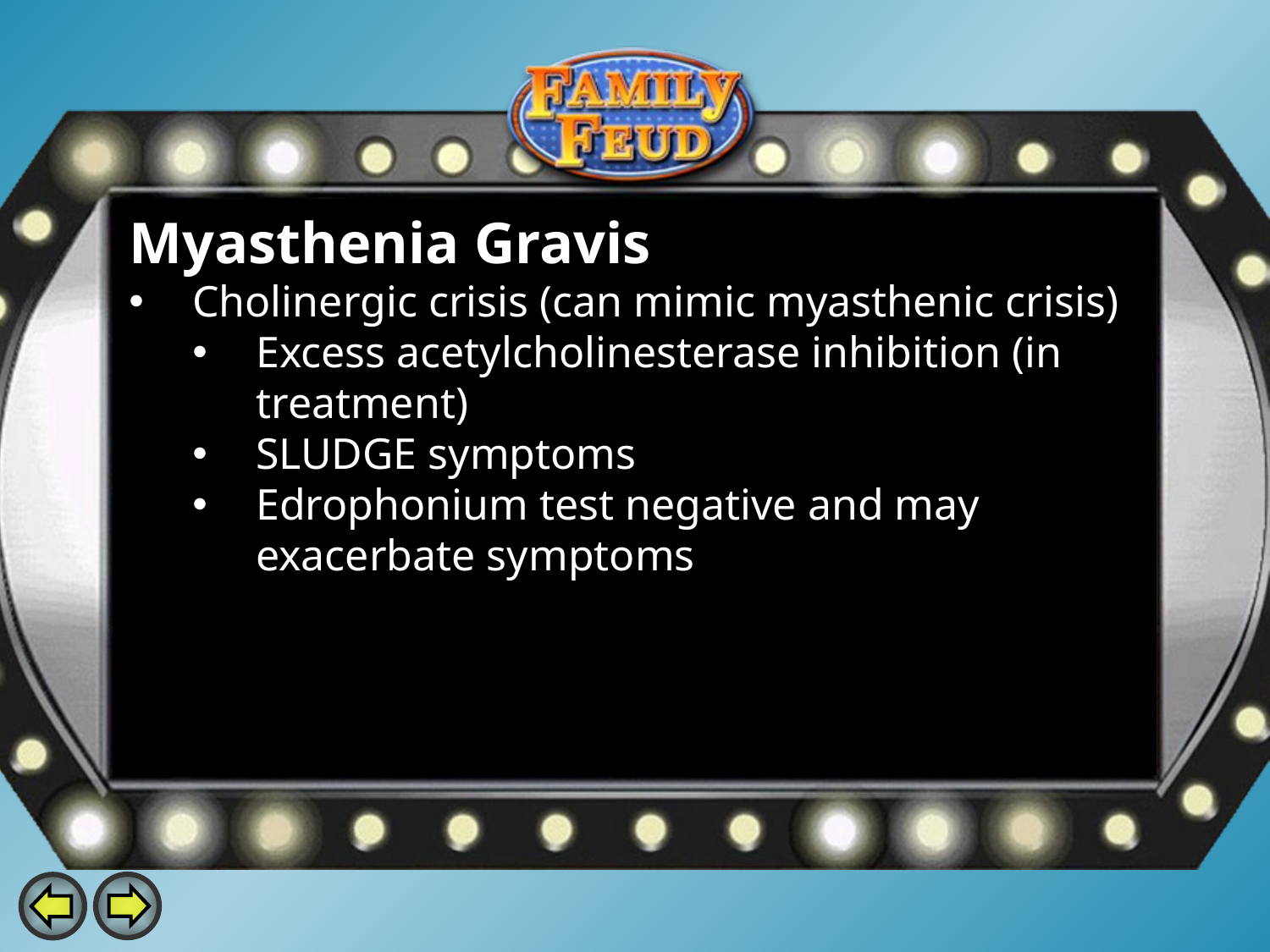

Myasthenia Gravis
Cholinergic crisis (can mimic myasthenic crisis)
Excess acetylcholinesterase inhibition (in treatment)
SLUDGE symptoms
Edrophonium test negative and may exacerbate symptoms

## Slide 48
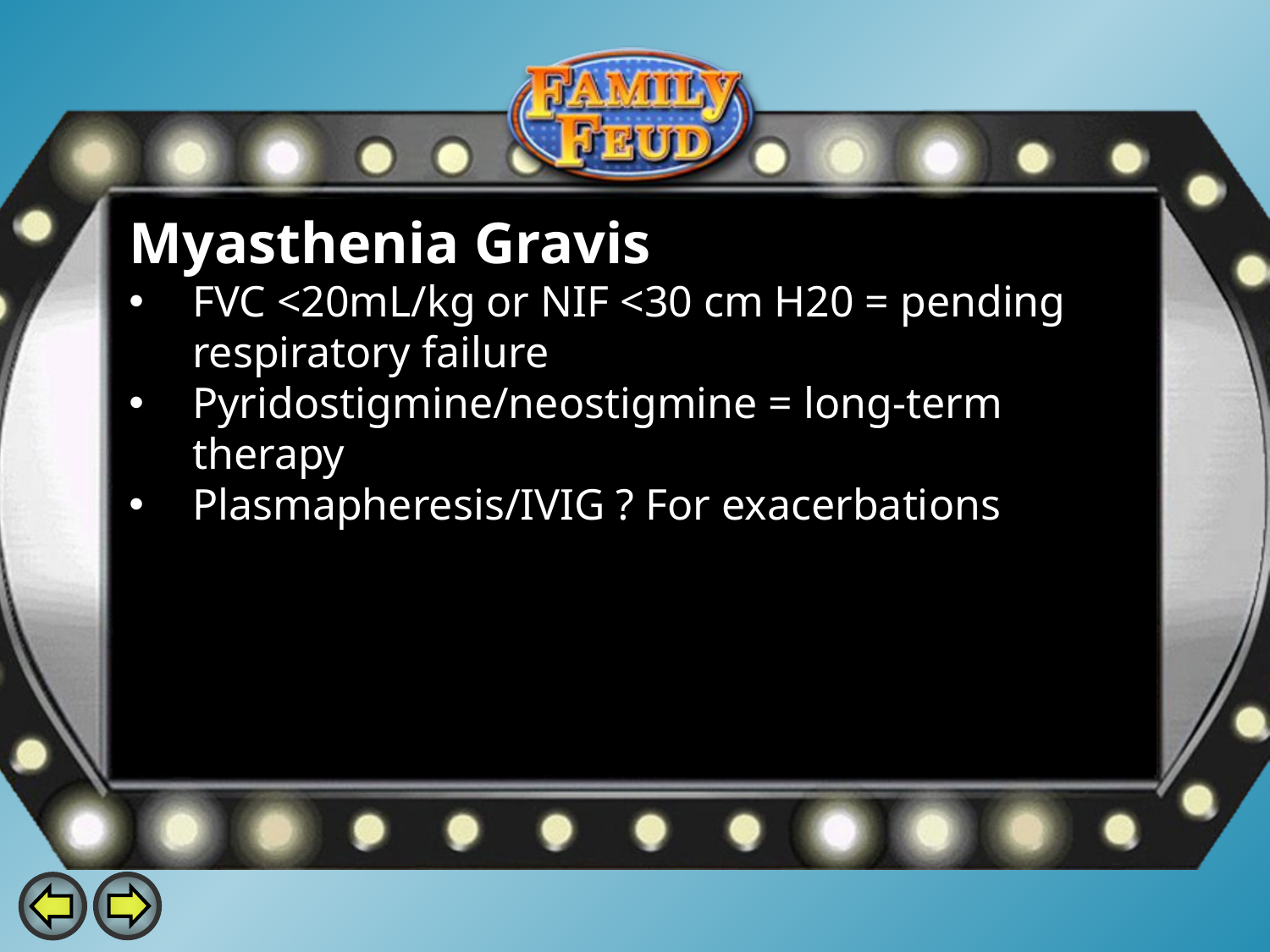

Myasthenia Gravis
FVC <20mL/kg or NIF <30 cm H20 = pending respiratory failure
Pyridostigmine/neostigmine = long-term therapy
Plasmapheresis/IVIG ? For exacerbations

## Slide 49
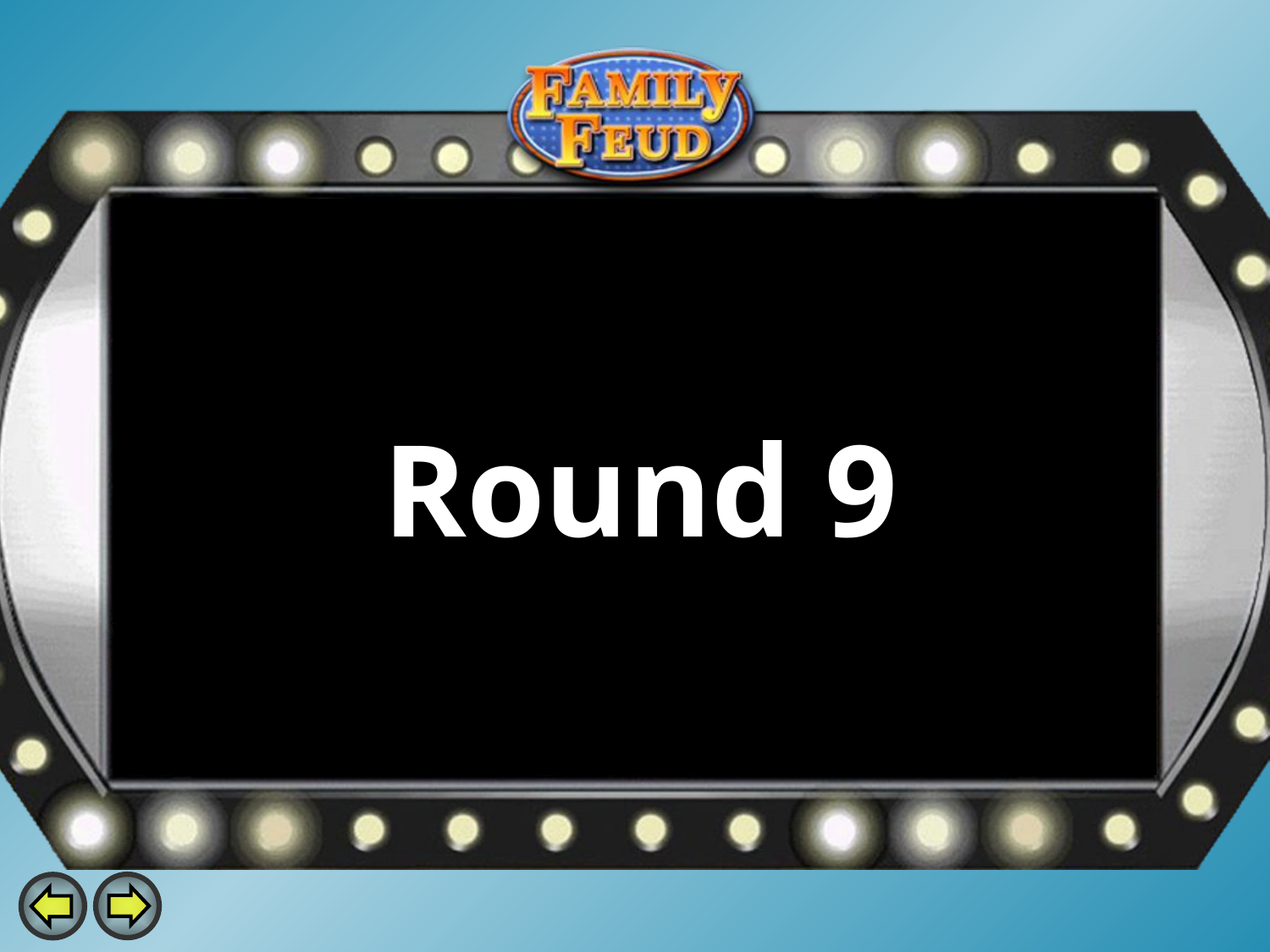

Round 9

## Slide 50
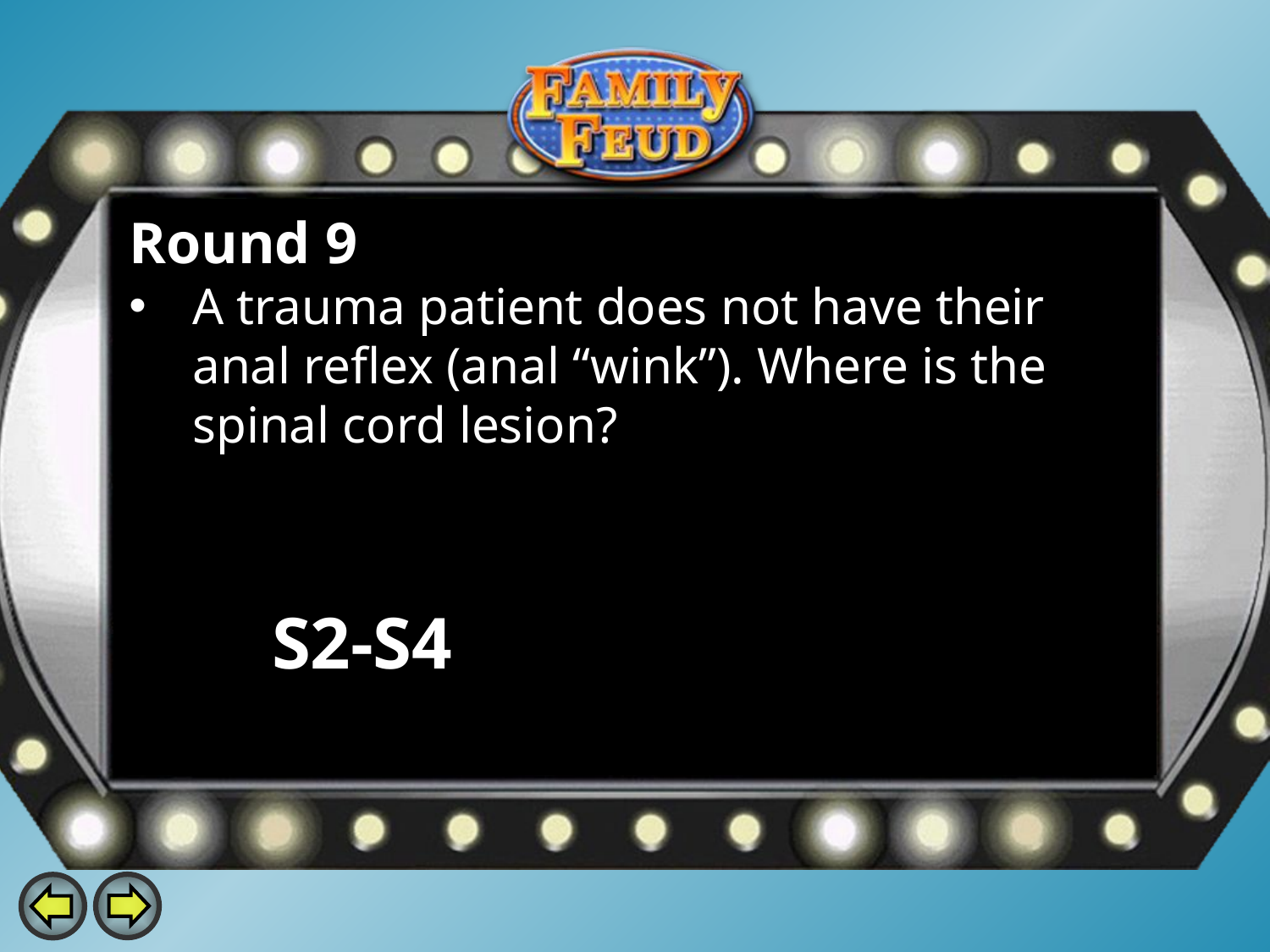

Round 9
A trauma patient does not have their anal reflex (anal “wink”). Where is the spinal cord lesion?
S2-S4

## Slide 51
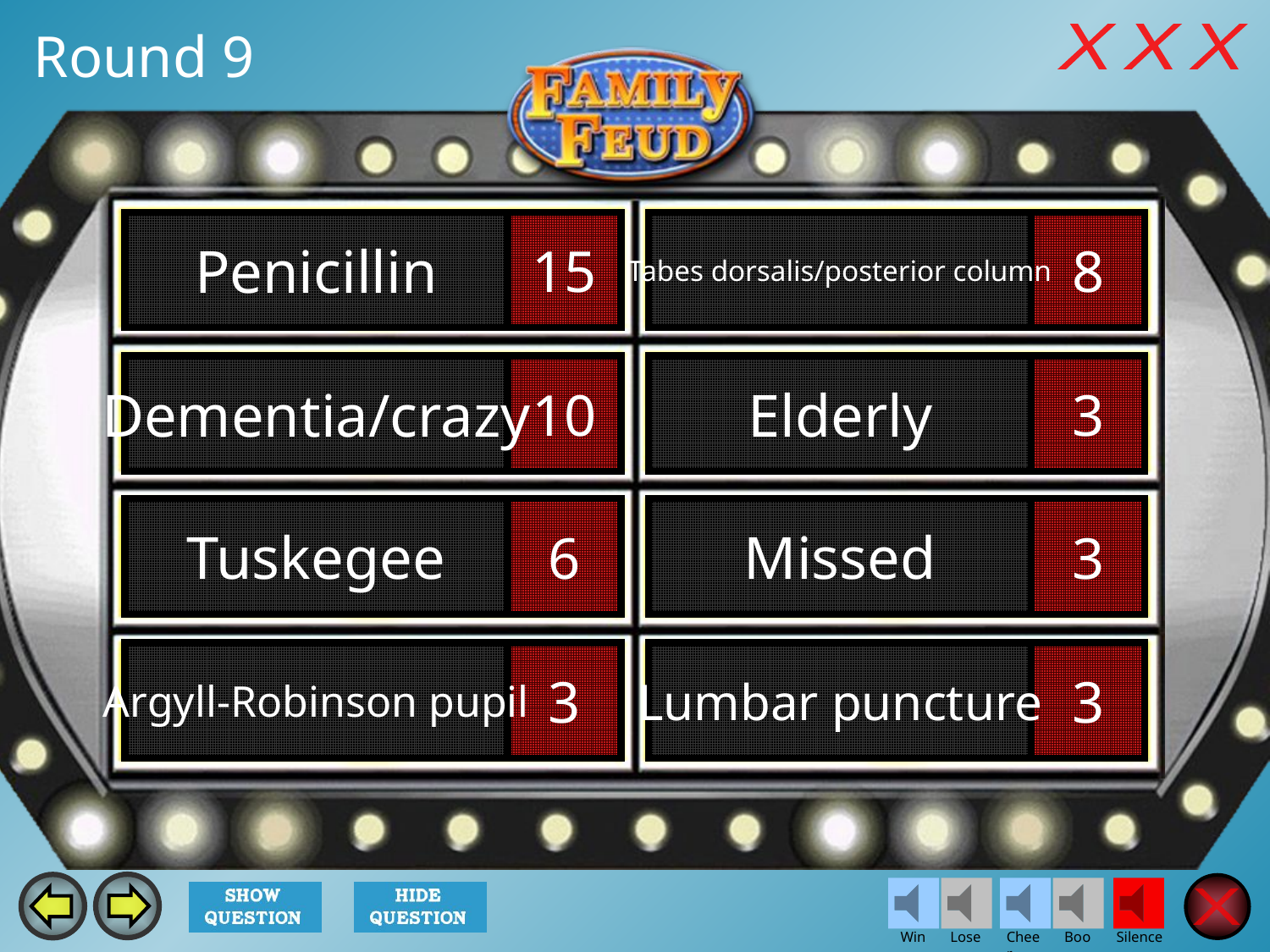

Neurosyphilis
X
X
X
X
X
X
Round 9
X
X
X
Penicillin
15
Tabes dorsalis/posterior column
8
Dementia/crazy
10
Elderly
3
Tuskegee
6
Missed
3
Argyll-Robinson pupil
3
Lumbar puncture
3
Win
Lose
Cheer
Boo
Silence
X
X
X

## Slide 52
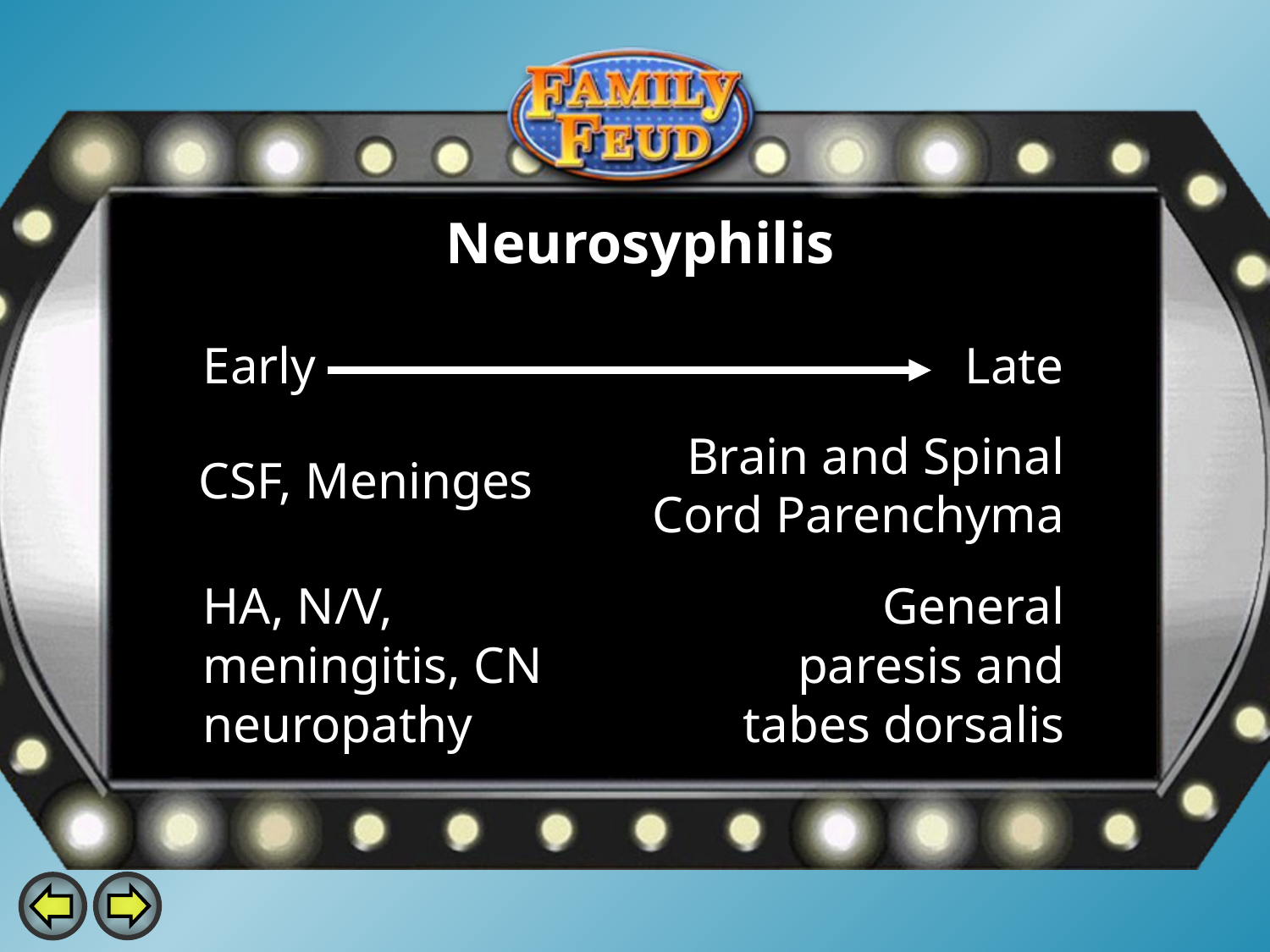

Neurosyphilis
Early		 				Late
Brain and Spinal Cord Parenchyma
CSF, Meninges
General paresis and tabes dorsalis
HA, N/V, meningitis, CN neuropathy

## Slide 53
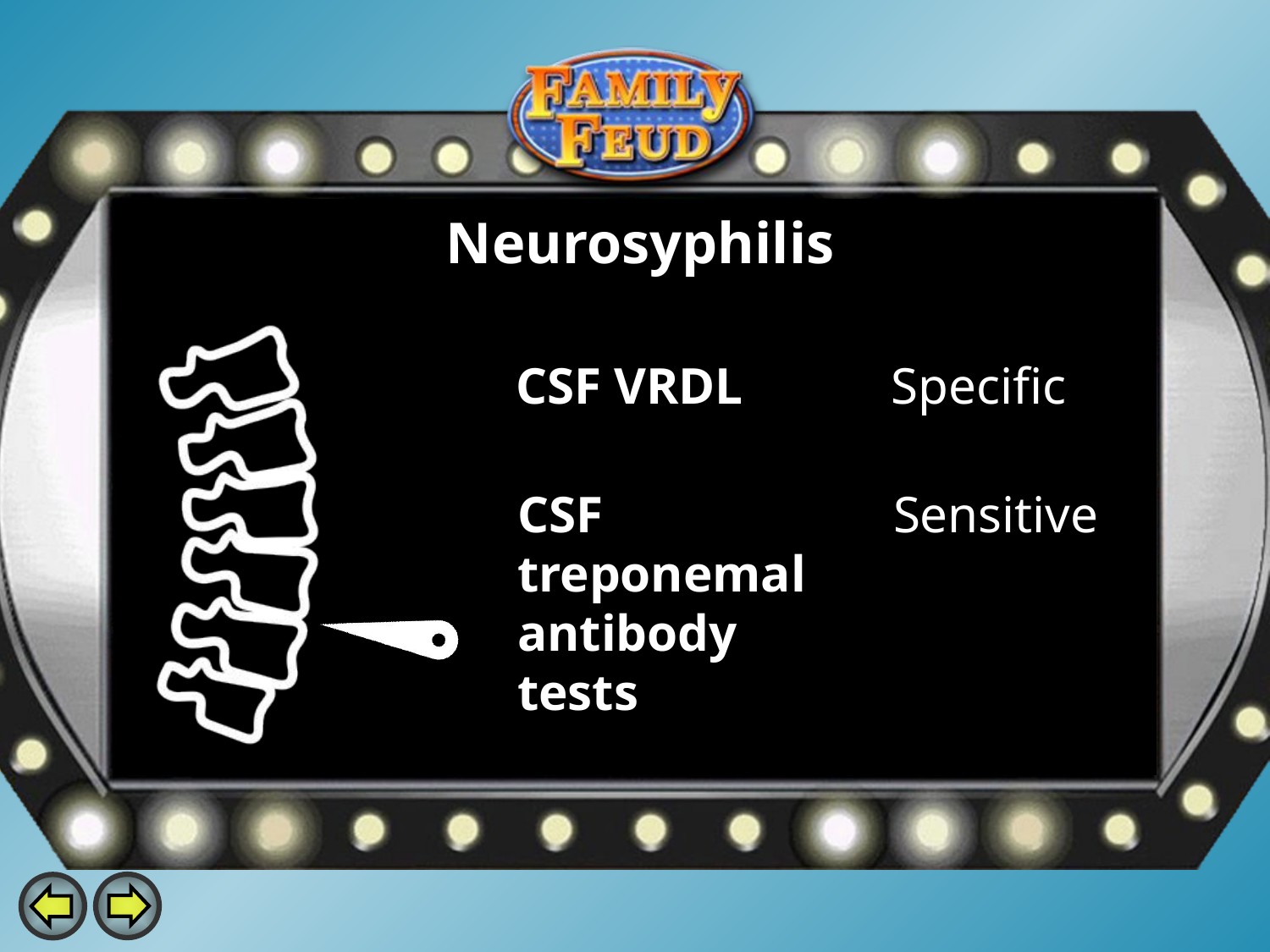

Neurosyphilis
CSF VRDL
Specific
CSF treponemal antibody tests
Sensitive

## Slide 54
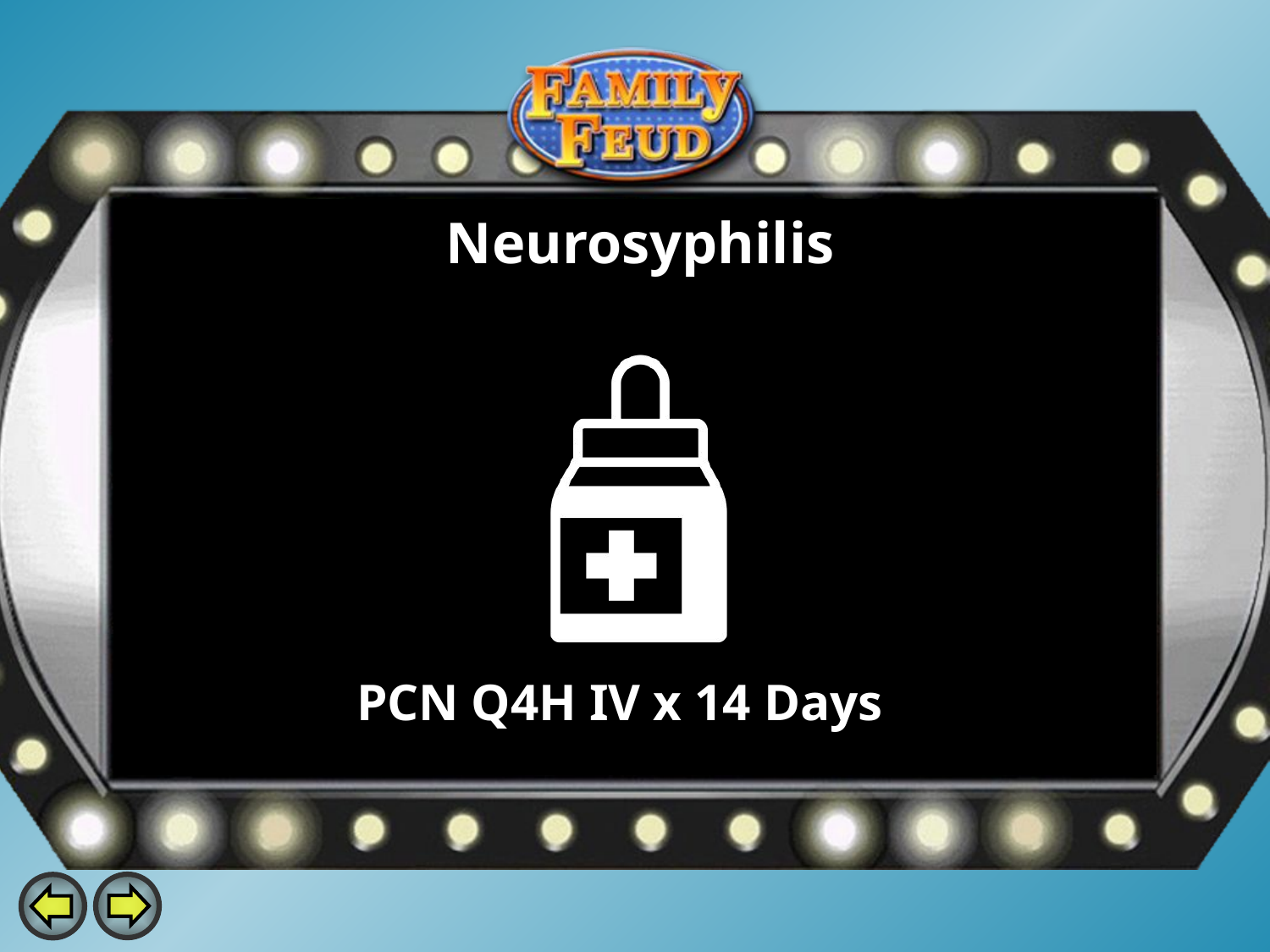

Neurosyphilis
PCN Q4H IV x 14 Days

## Slide 55
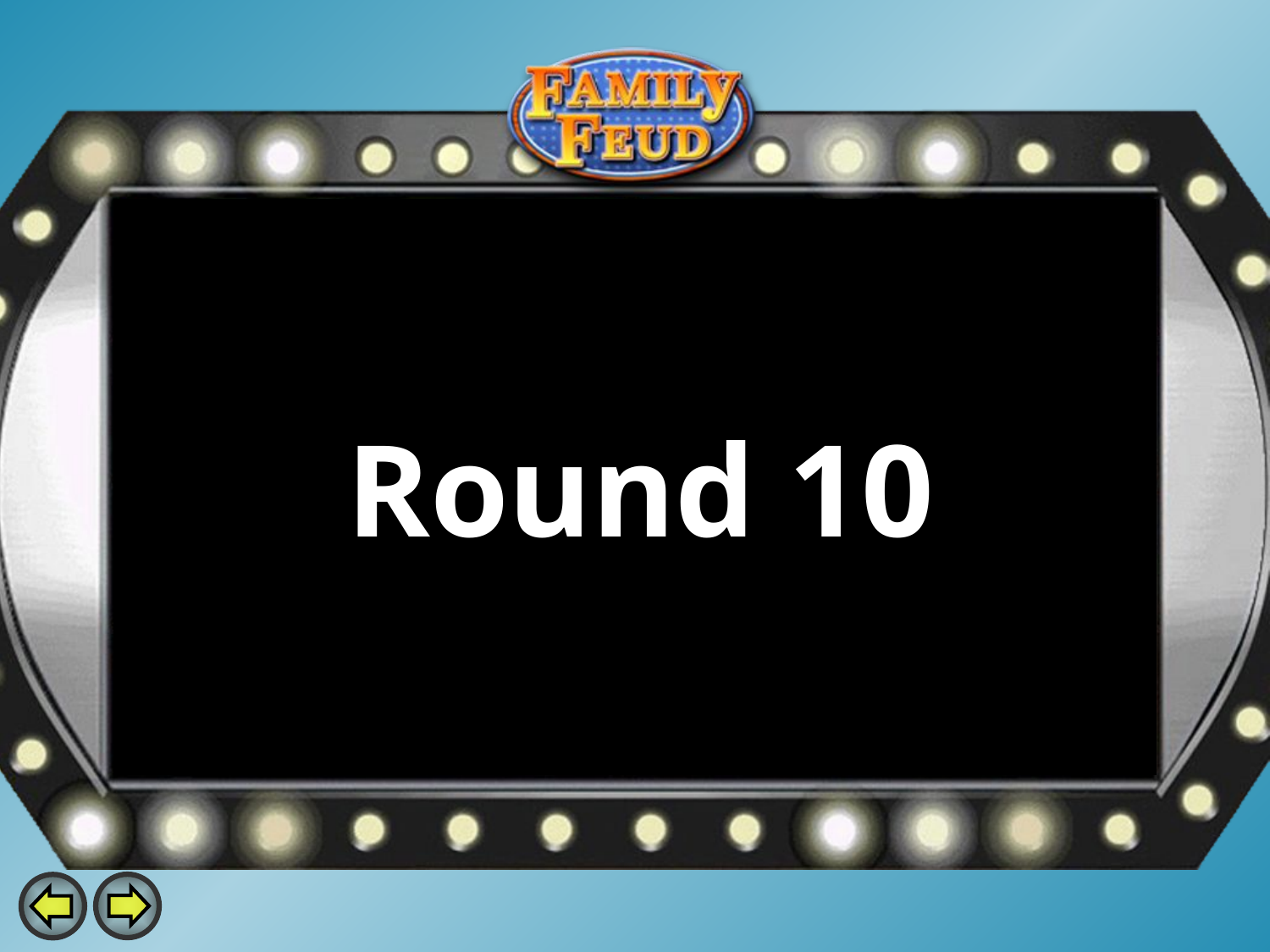

Round 10

## Slide 56
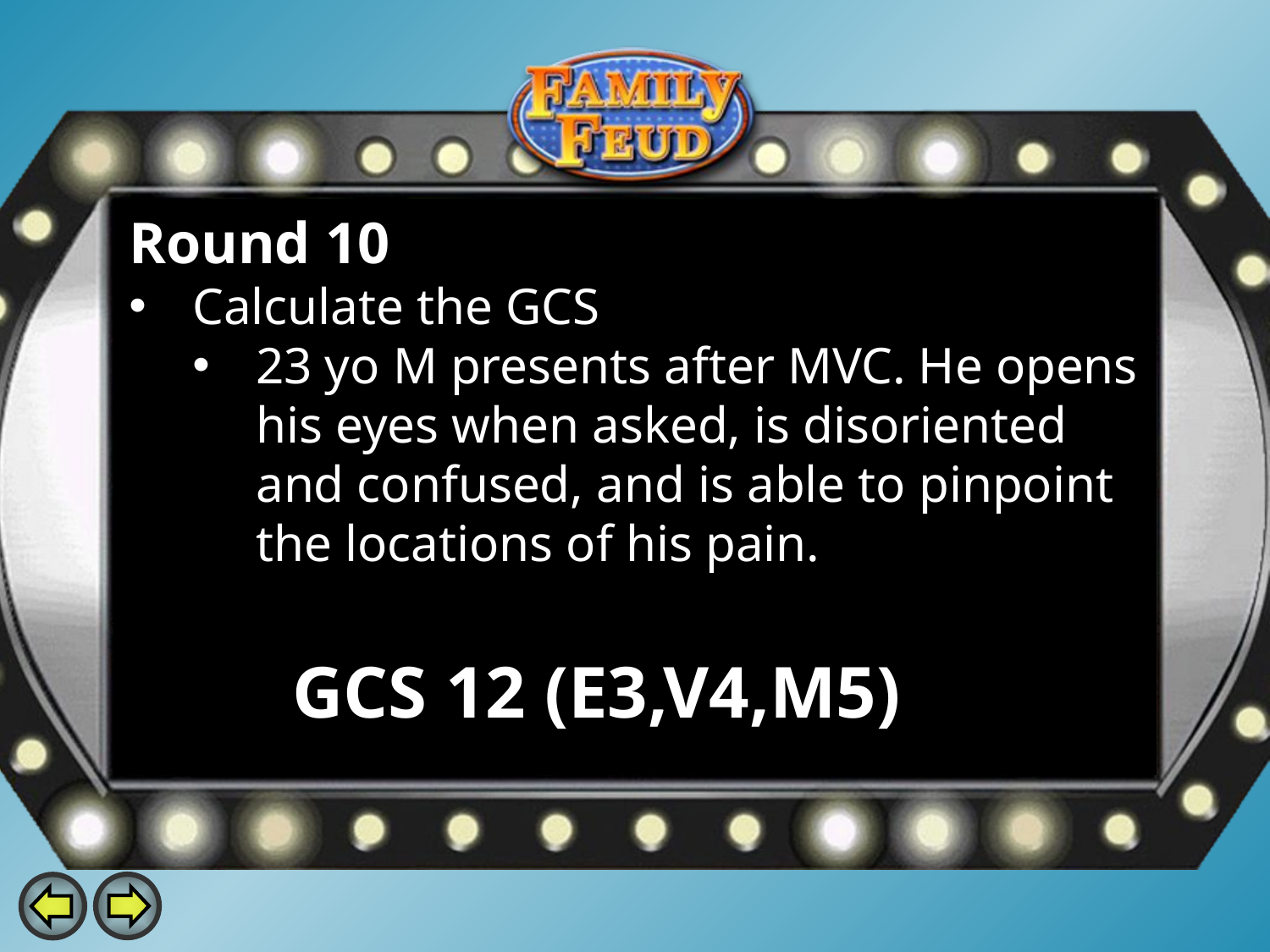

Round 10
Calculate the GCS
23 yo M presents after MVC. He opens his eyes when asked, is disoriented and confused, and is able to pinpoint the locations of his pain.
GCS 12 (E3,V4,M5)

## Slide 57
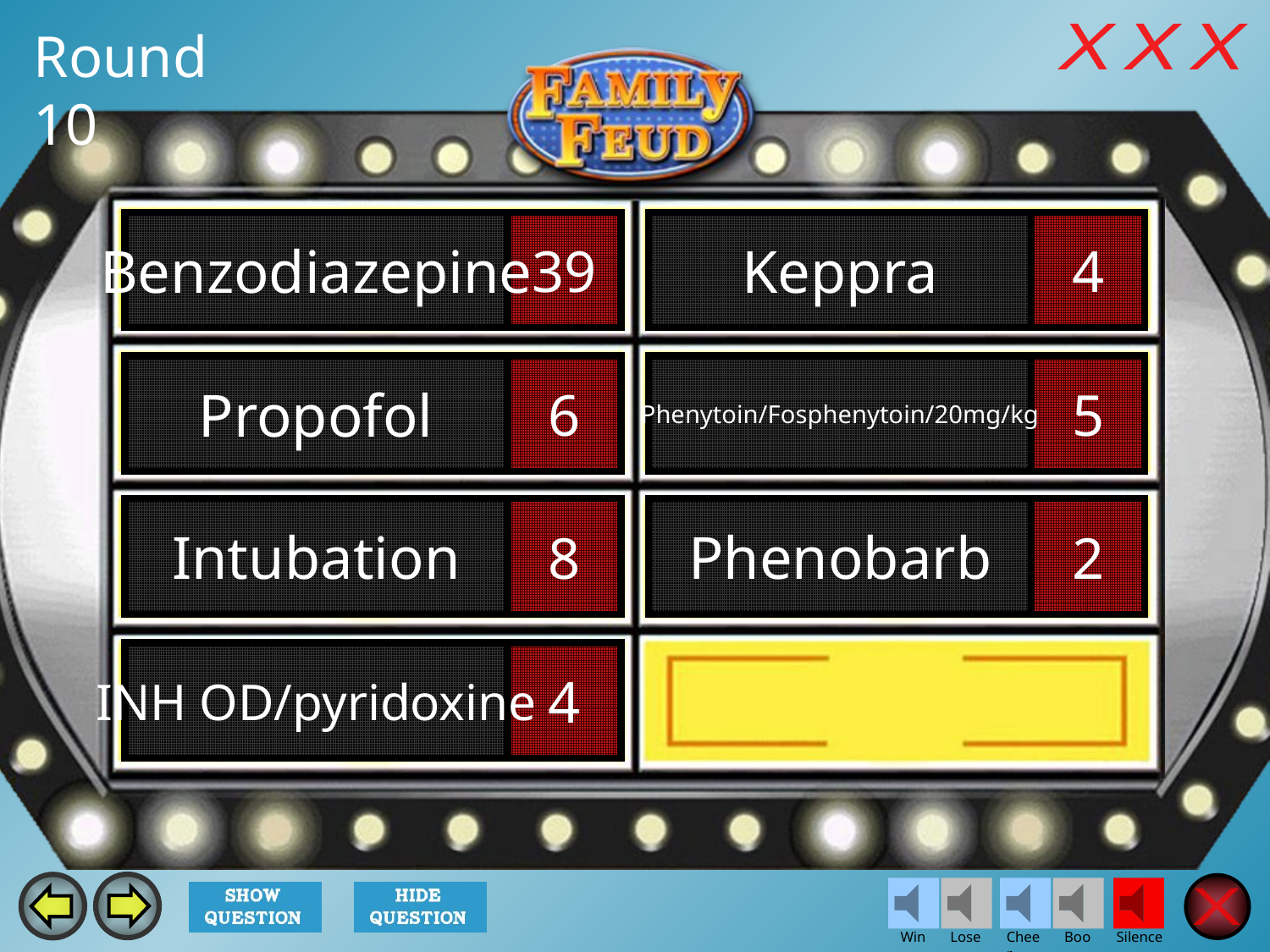

Status Epilepticus
X
X
X
X
X
X
Round 10
X
X
X
Benzodiazepine
39
Keppra
4
Propofol
6
Phenytoin/Fosphenytoin/20mg/kg
5
Intubation
8
Phenobarb
2
INH OD/pyridoxine
4
Win
Lose
Cheer
Boo
Silence
X
X
X

## Slide 58
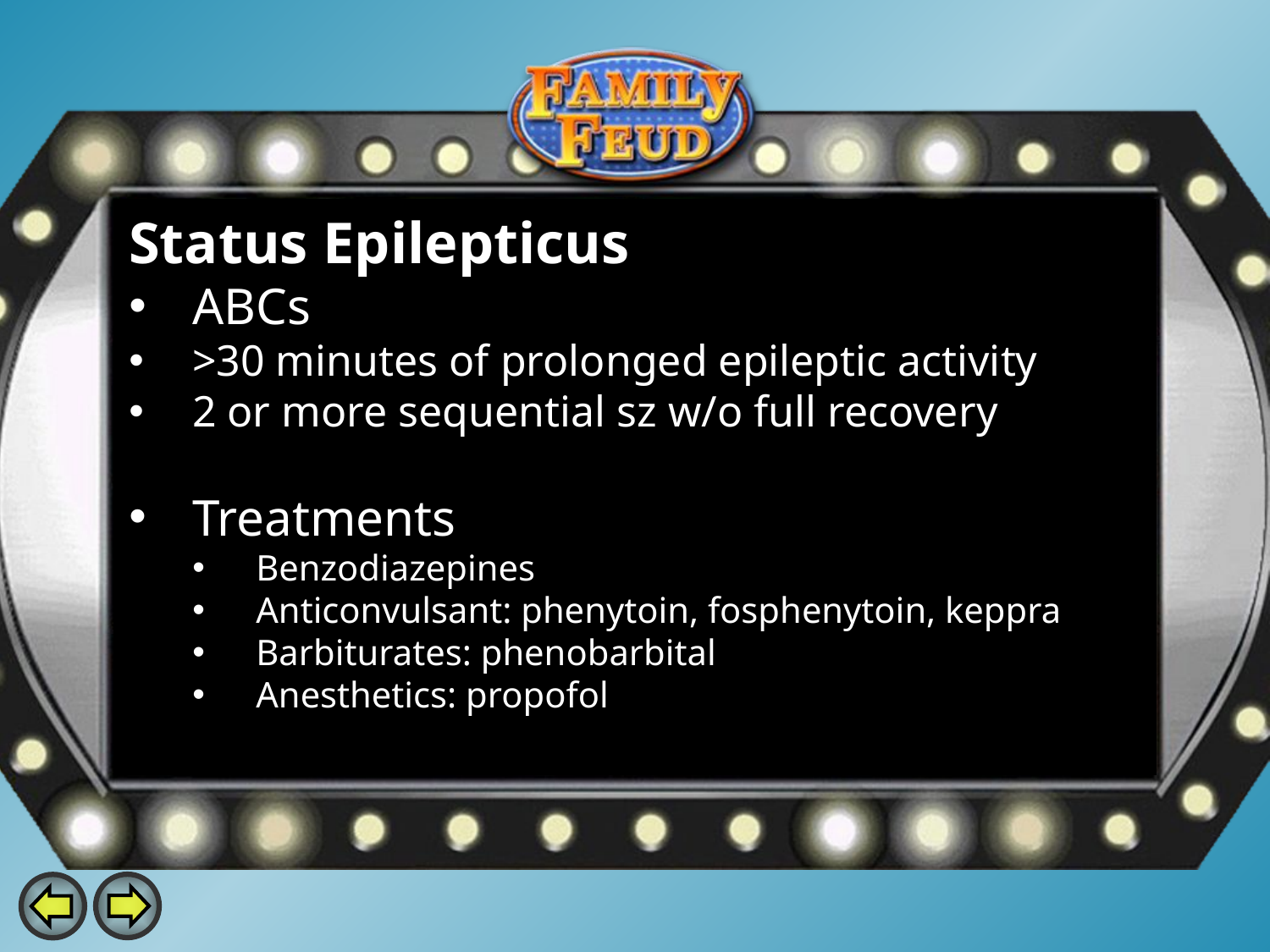

Status Epilepticus
ABCs
>30 minutes of prolonged epileptic activity
2 or more sequential sz w/o full recovery
Treatments
Benzodiazepines
Anticonvulsant: phenytoin, fosphenytoin, keppra
Barbiturates: phenobarbital
Anesthetics: propofol

## Slide 59
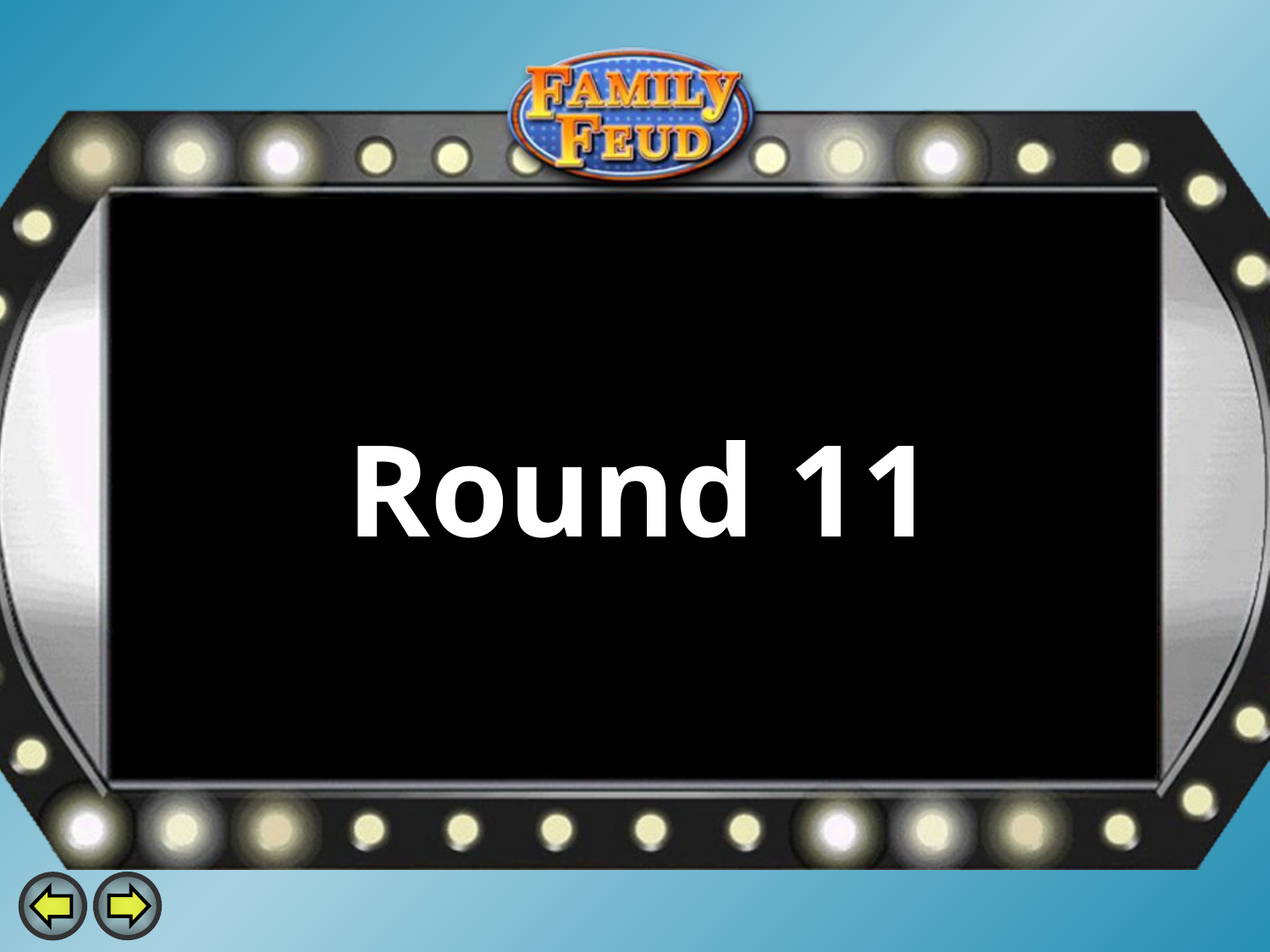

Round 11

## Slide 60
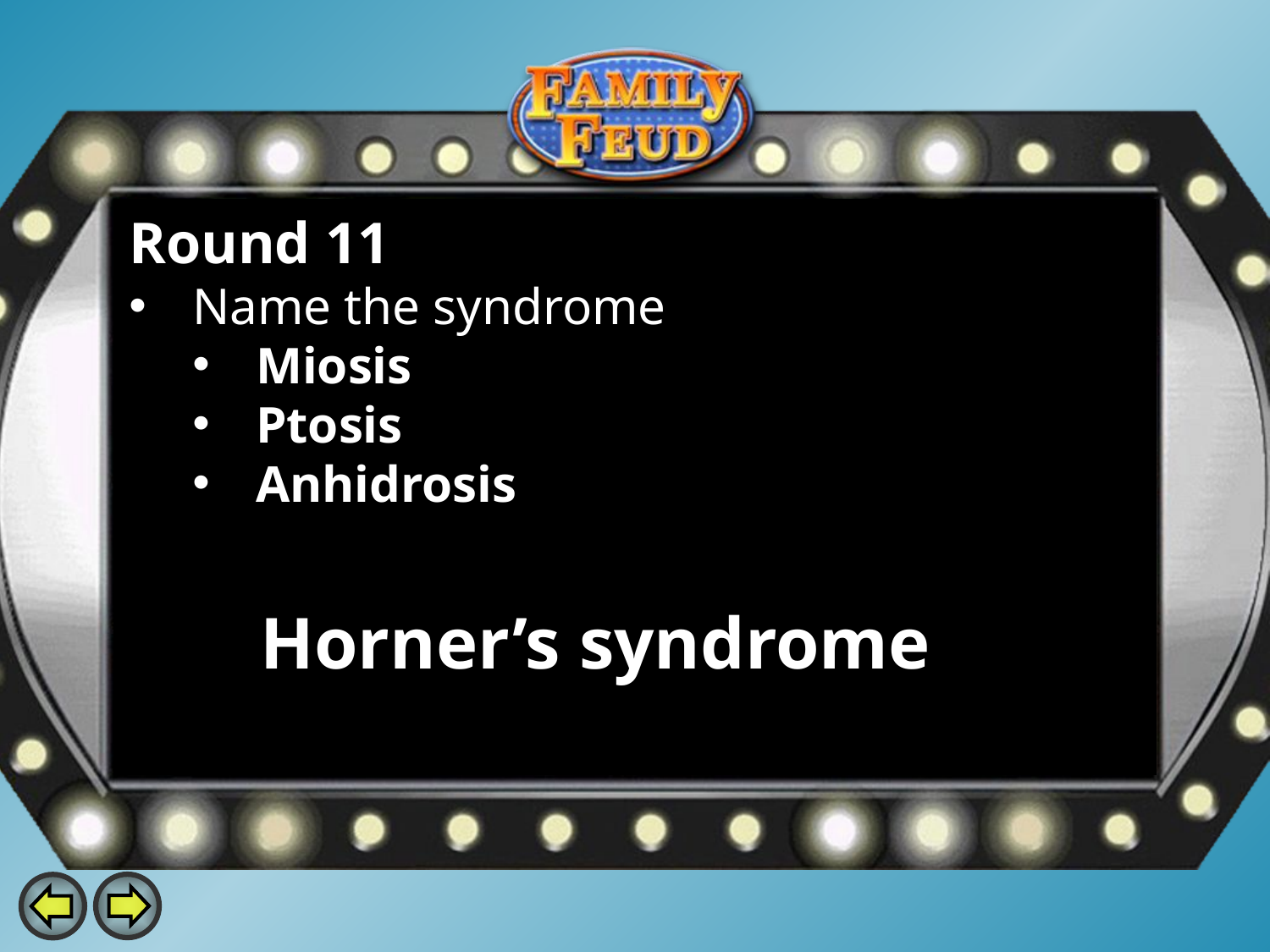

Round 11
Name the syndrome
Miosis
Ptosis
Anhidrosis
Horner’s syndrome

## Slide 61
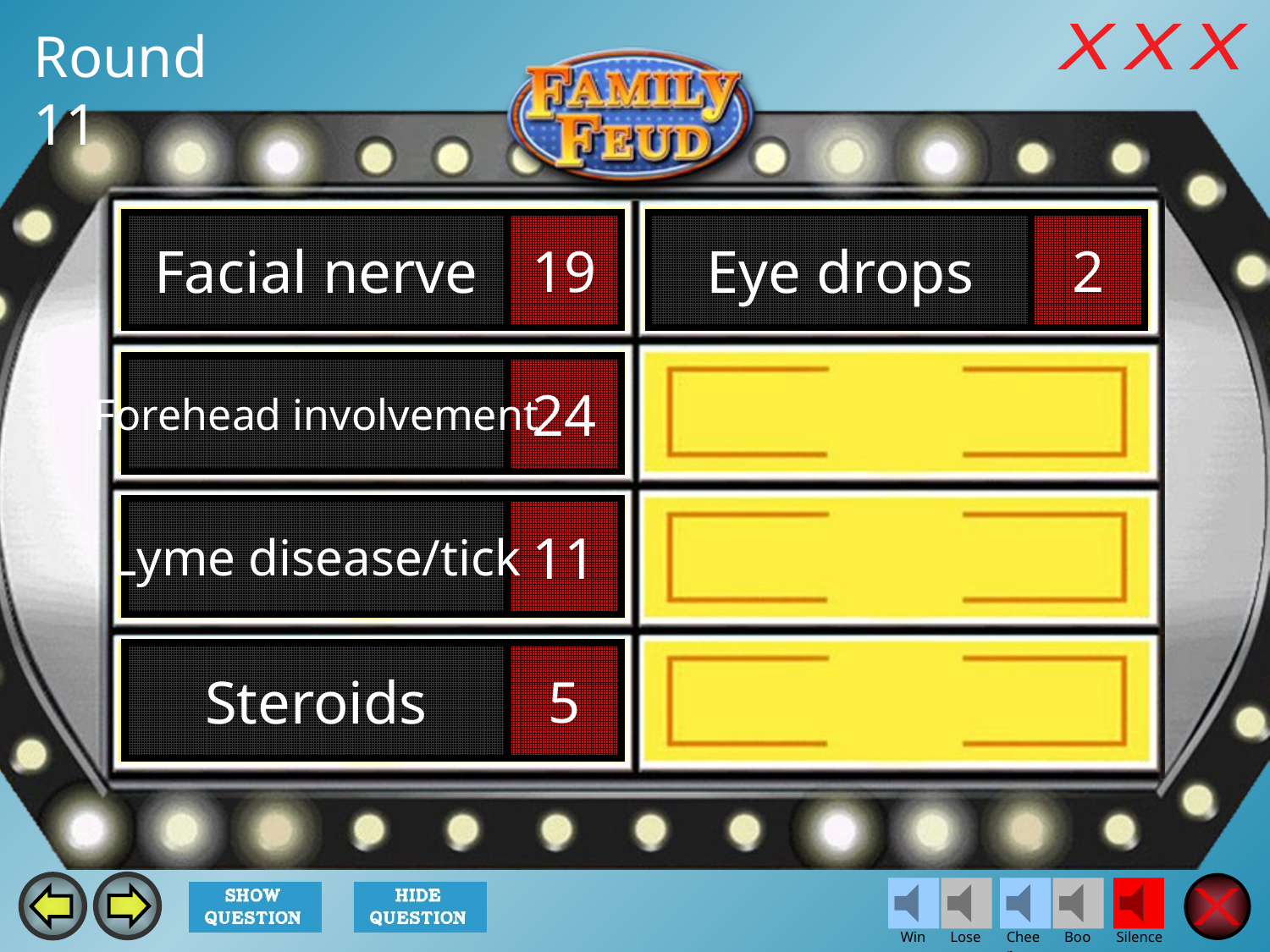

Bell’s Palsy
X
X
X
X
X
X
Round 11
X
X
X
Facial nerve
19
Eye drops
2
Forehead involvement
24
Lyme disease/tick
11
Steroids
5
Win
Lose
Cheer
Boo
Silence
X
X
X

## Slide 62
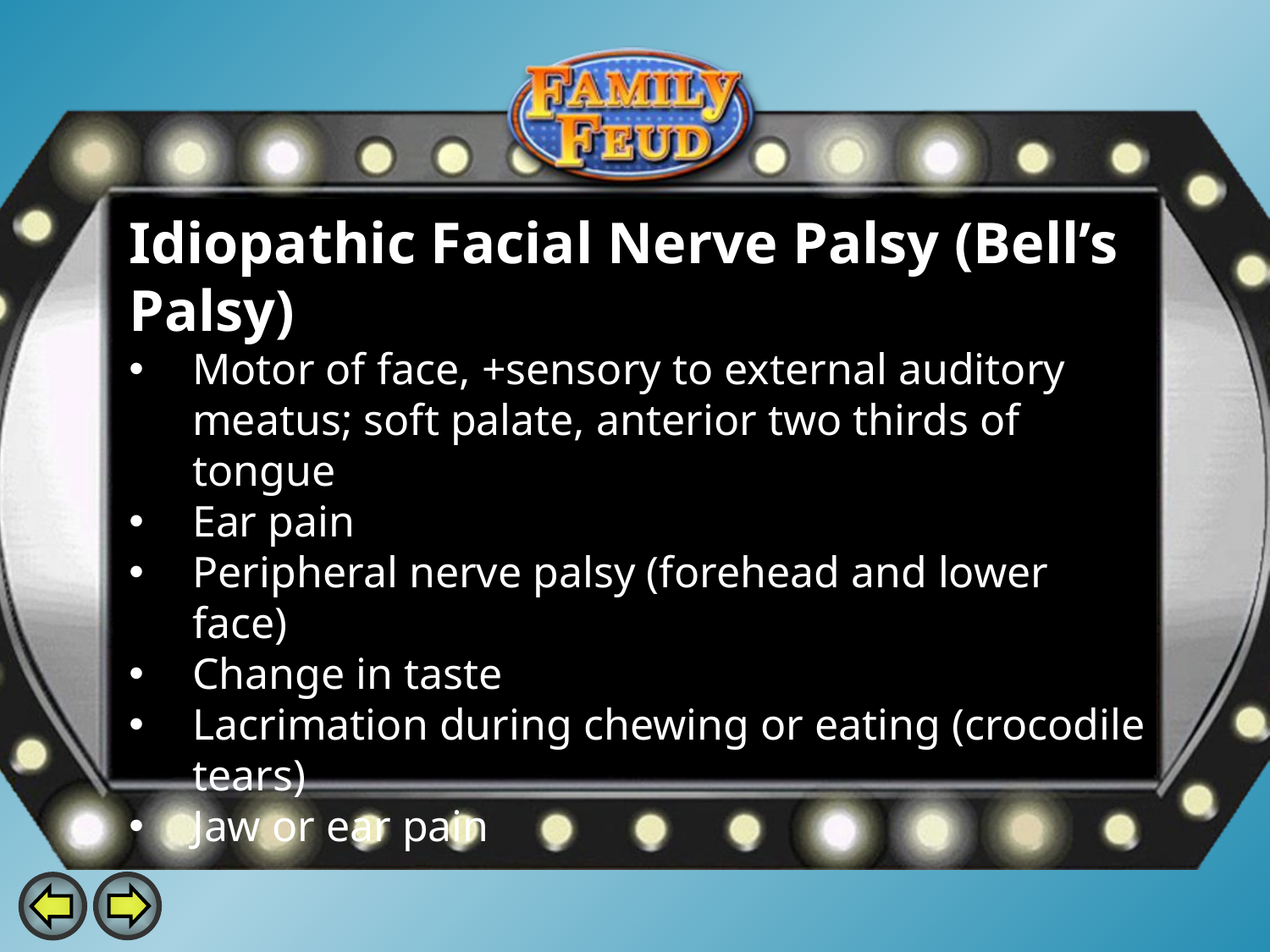

Idiopathic Facial Nerve Palsy (Bell’s Palsy)
Motor of face, +sensory to external auditory meatus; soft palate, anterior two thirds of tongue
Ear pain
Peripheral nerve palsy (forehead and lower face)
Change in taste
Lacrimation during chewing or eating (crocodile tears)
Jaw or ear pain

## Slide 63
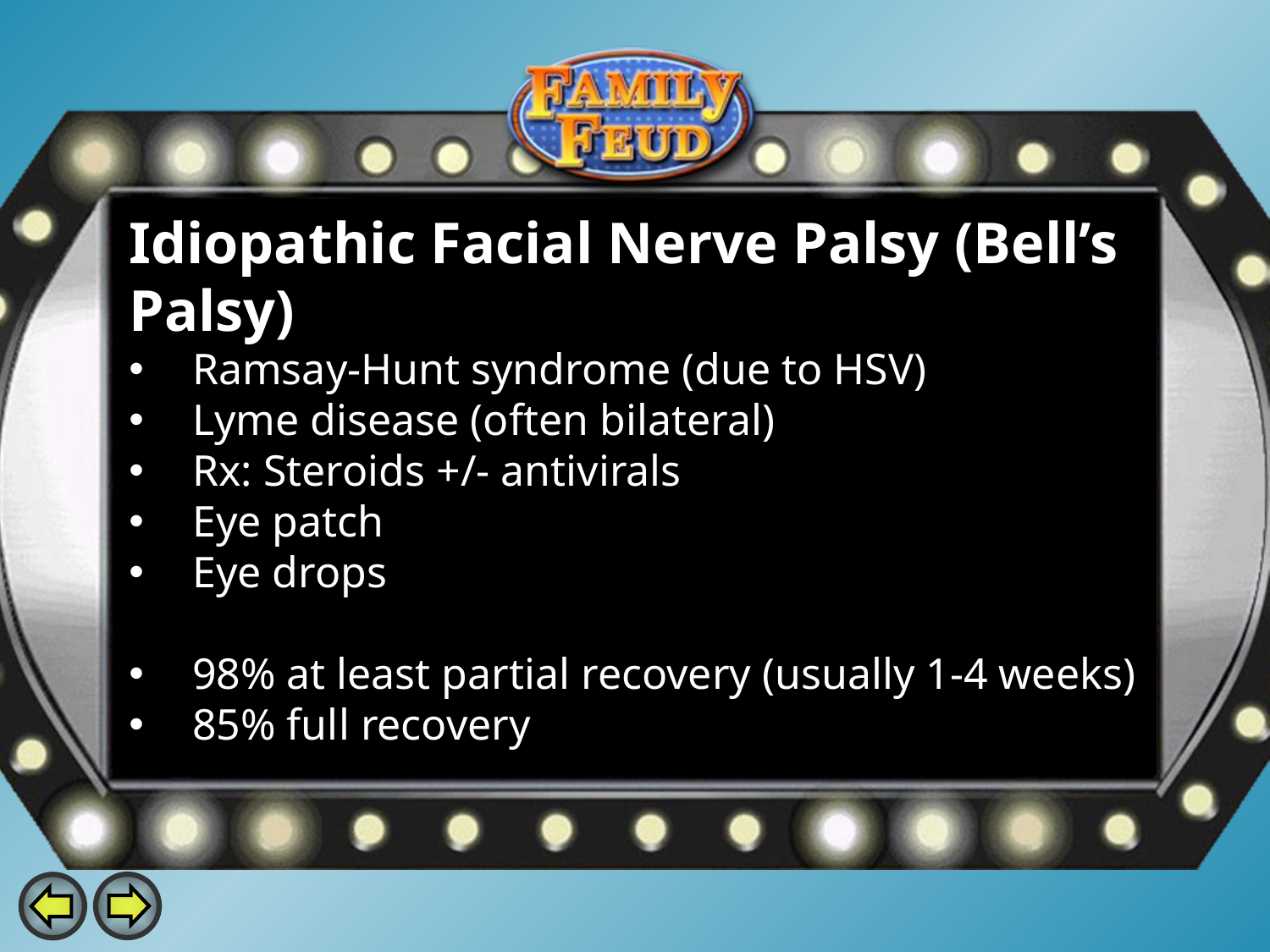

Idiopathic Facial Nerve Palsy (Bell’s Palsy)
Ramsay-Hunt syndrome (due to HSV)
Lyme disease (often bilateral)
Rx: Steroids +/- antivirals
Eye patch
Eye drops
98% at least partial recovery (usually 1-4 weeks)
85% full recovery

## Slide 64
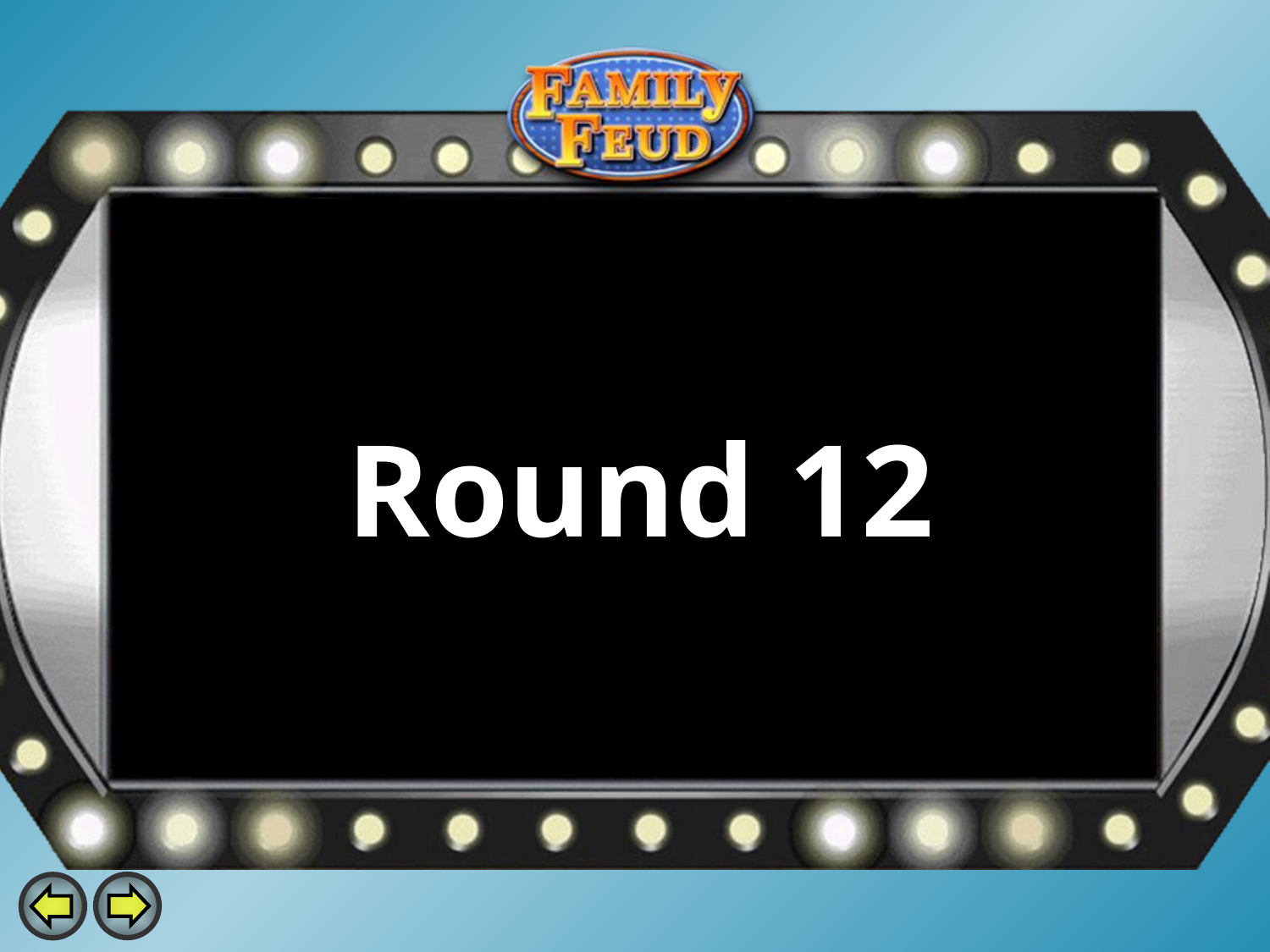

Round 12

## Slide 65
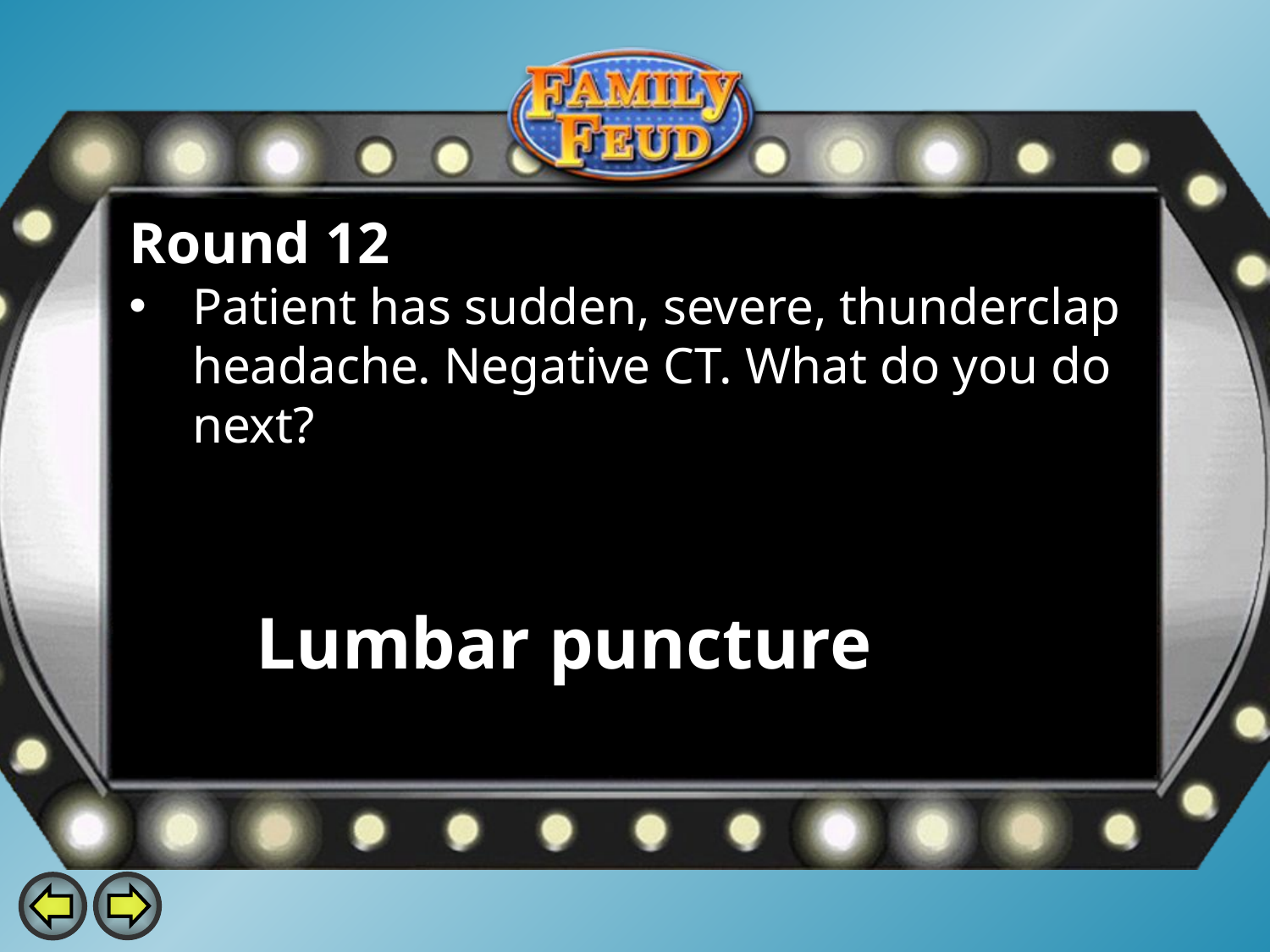

Round 12
Patient has sudden, severe, thunderclap headache. Negative CT. What do you do next?
Lumbar puncture

## Slide 66
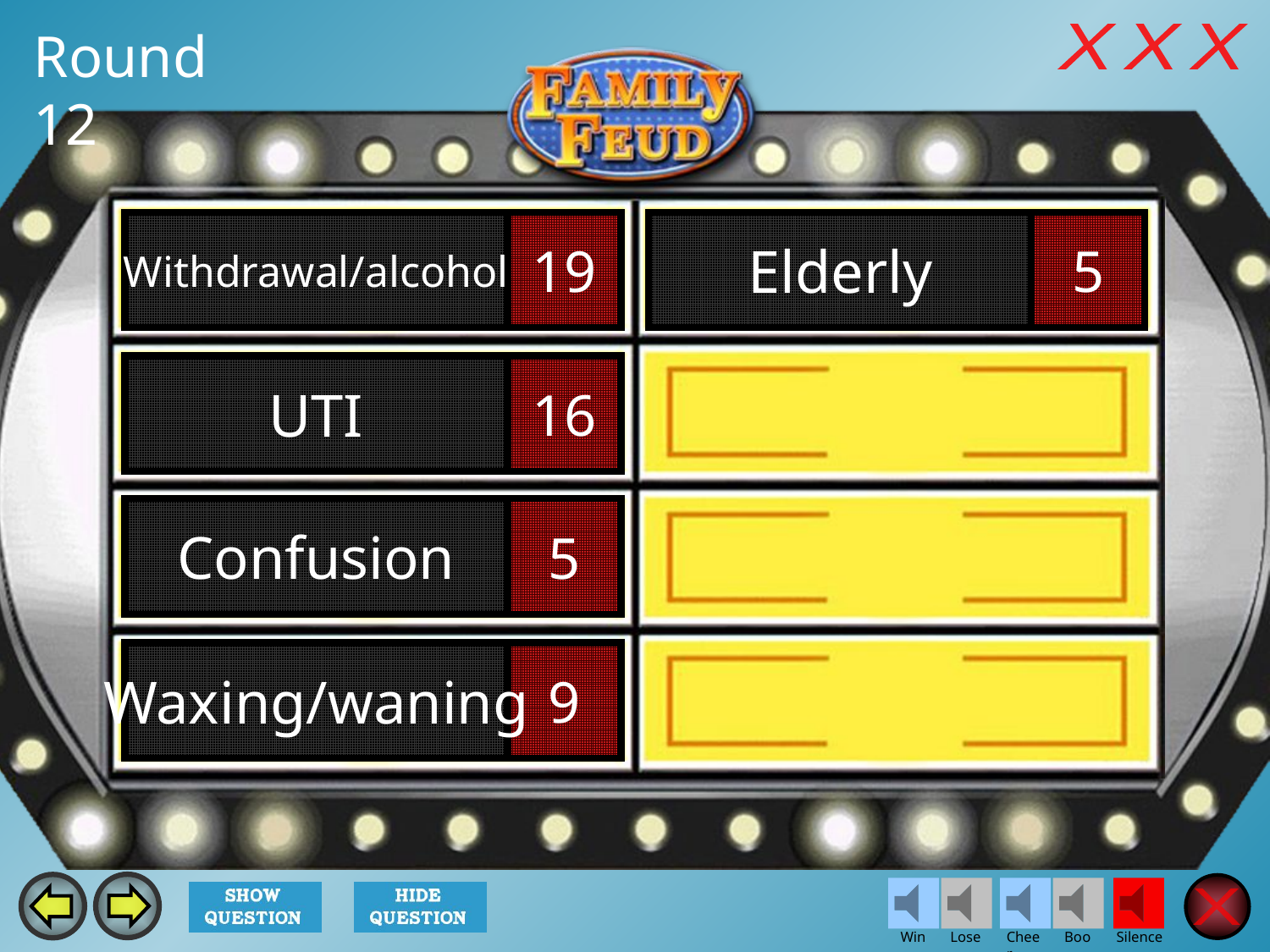

Delirium
X
X
X
X
X
X
Round 12
X
X
X
Withdrawal/alcohol
19
Elderly
5
UTI
16
Confusion
5
Waxing/waning
9
Win
Lose
Cheer
Boo
Silence
X
X
X

## Slide 67
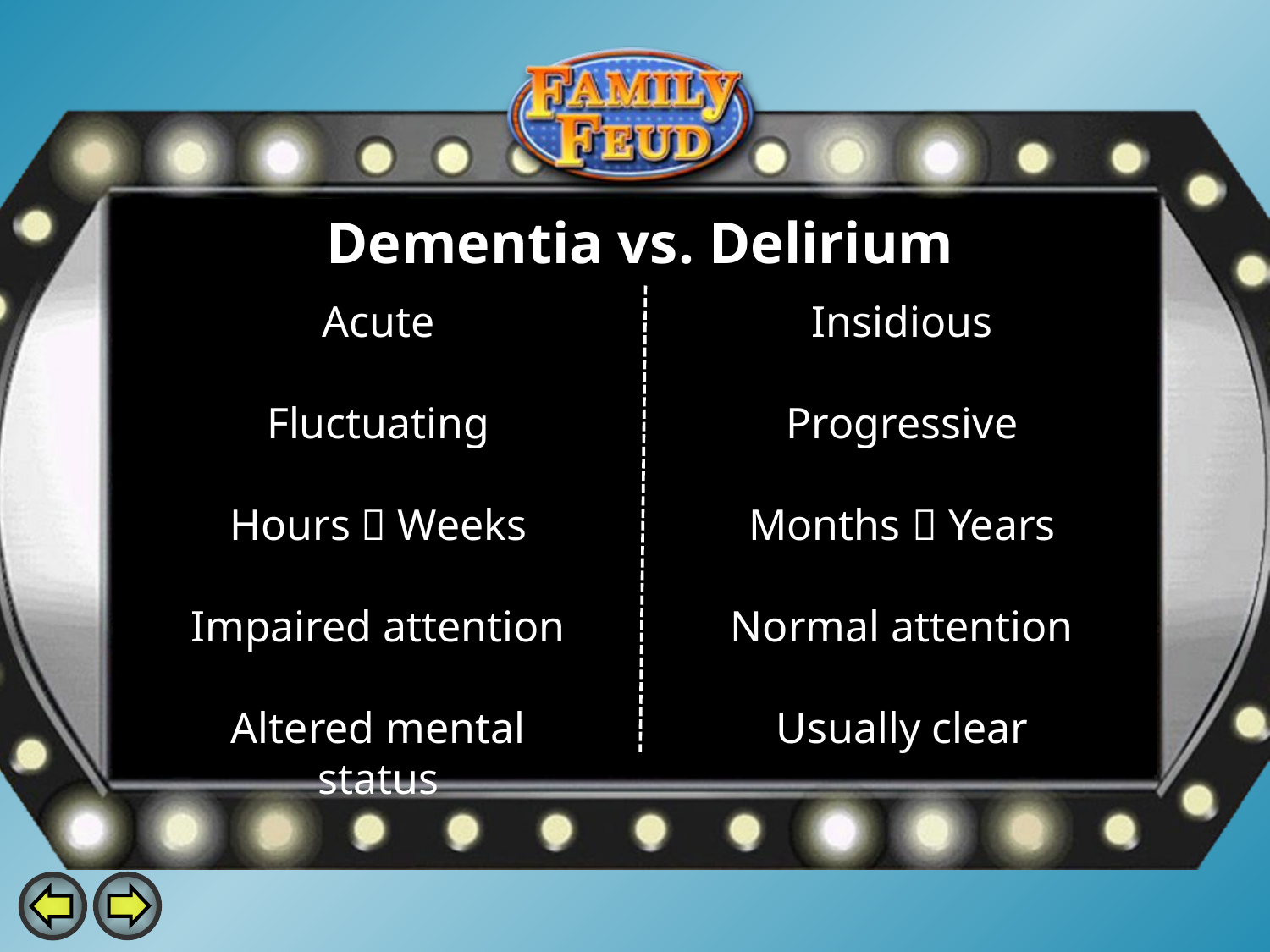

Dementia vs. Delirium
Acute
Fluctuating
Hours  Weeks
Impaired attention
Altered mental status
Insidious
Progressive
Months  Years
Normal attention
Usually clear

## Slide 68
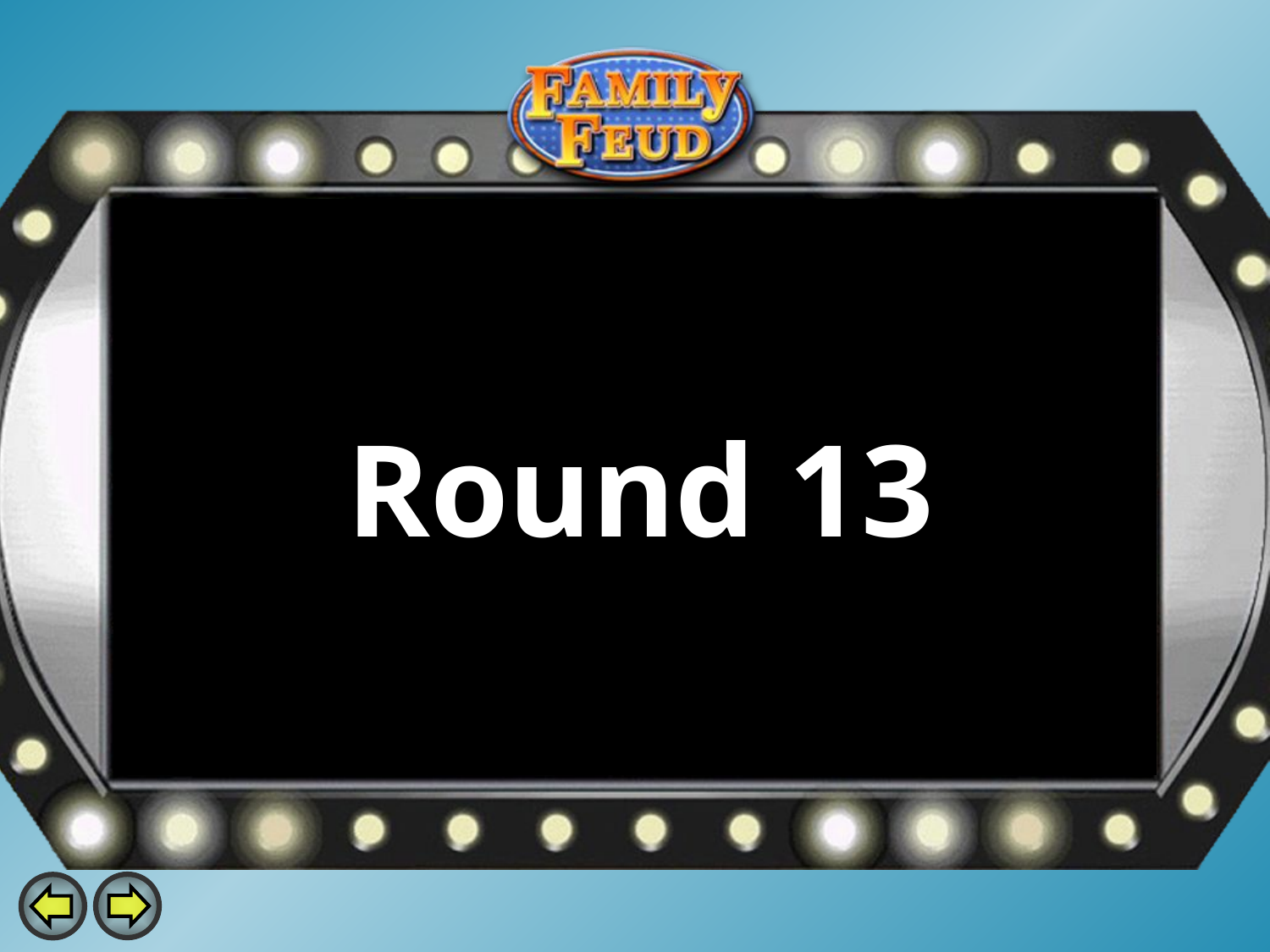

Round 13

## Slide 69
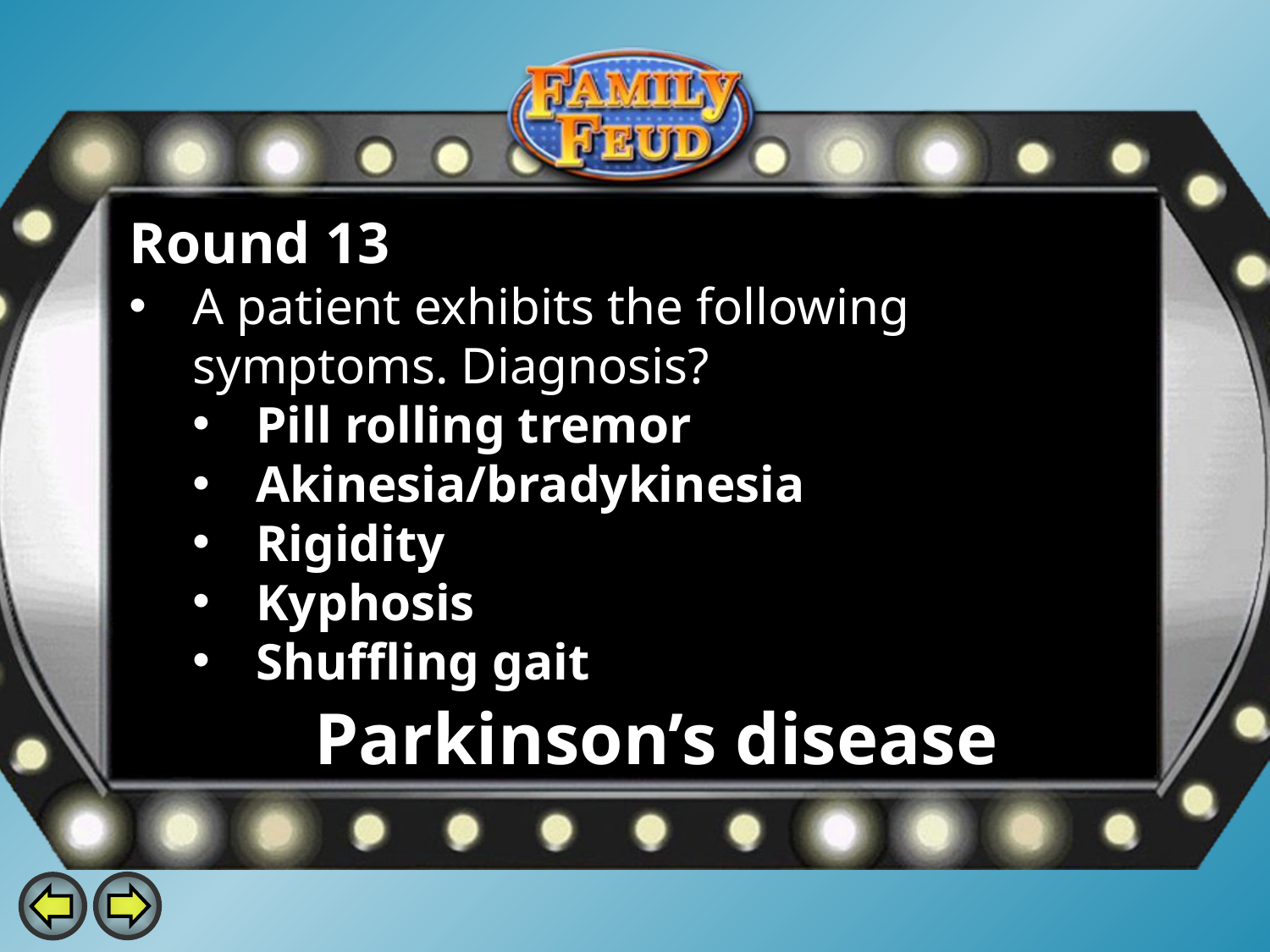

Round 13
A patient exhibits the following symptoms. Diagnosis?
Pill rolling tremor
Akinesia/bradykinesia
Rigidity
Kyphosis
Shuffling gait
Parkinson’s disease

## Slide 70
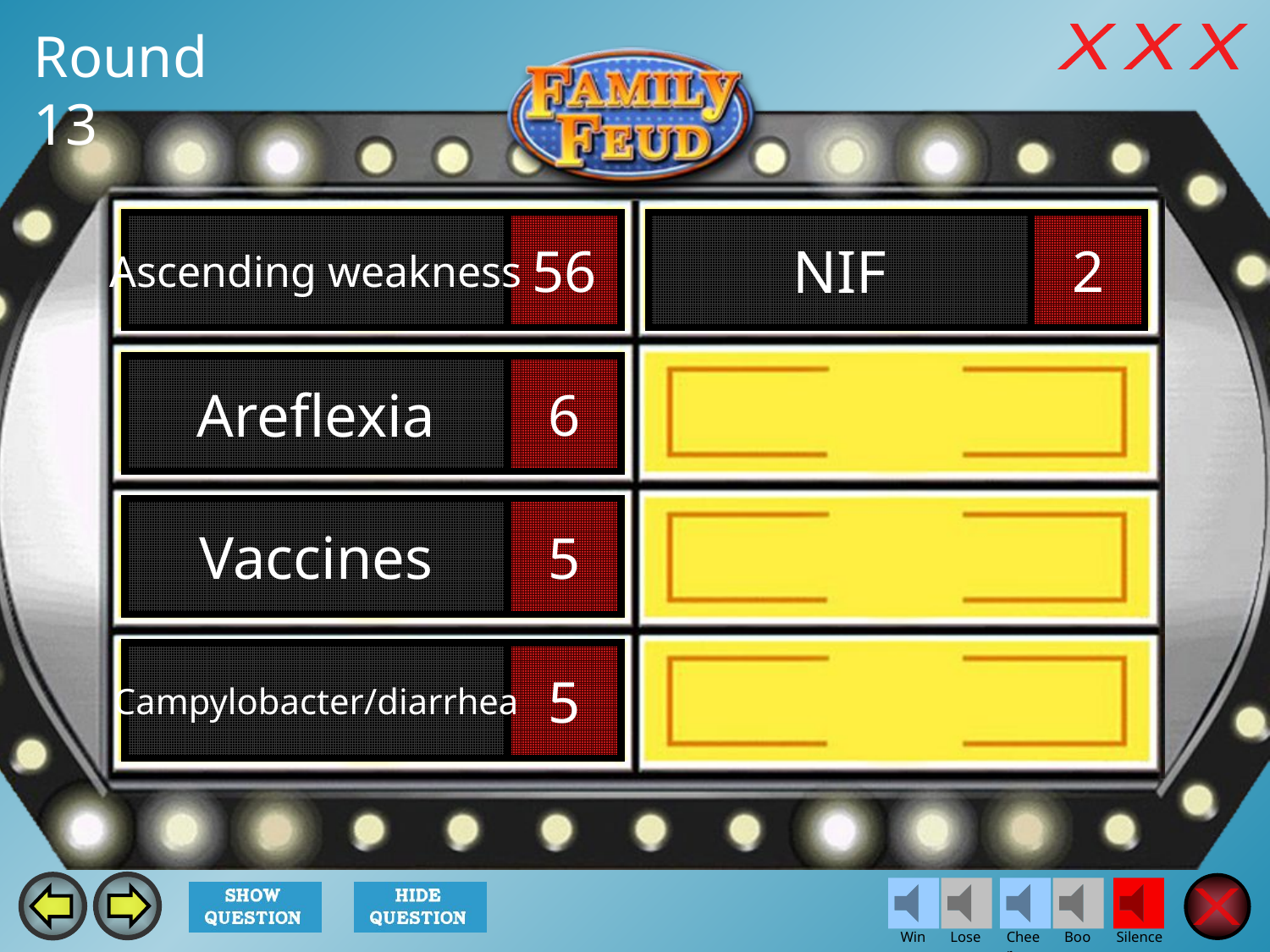

Guillain-Barré
X
X
X
X
X
X
Round 13
X
X
X
Ascending weakness
56
NIF
2
Areflexia
6
Vaccines
5
Campylobacter/diarrhea
5
Win
Lose
Cheer
Boo
Silence
X
X
X

## Slide 71
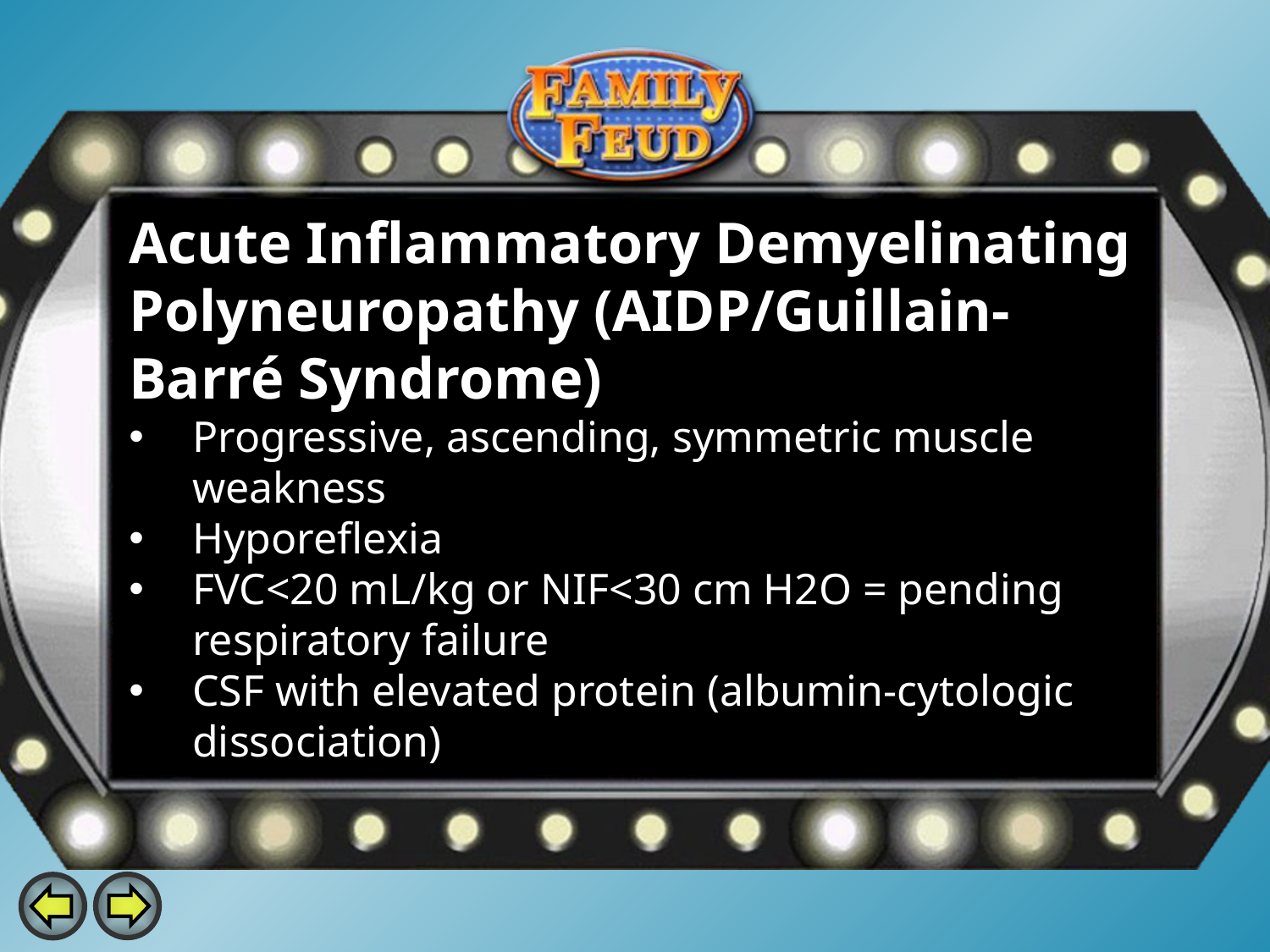

Acute Inflammatory Demyelinating Polyneuropathy (AIDP/Guillain-Barré Syndrome)
Progressive, ascending, symmetric muscle weakness
Hyporeflexia
FVC<20 mL/kg or NIF<30 cm H2O = pending respiratory failure
CSF with elevated protein (albumin-cytologic dissociation)

## Slide 72
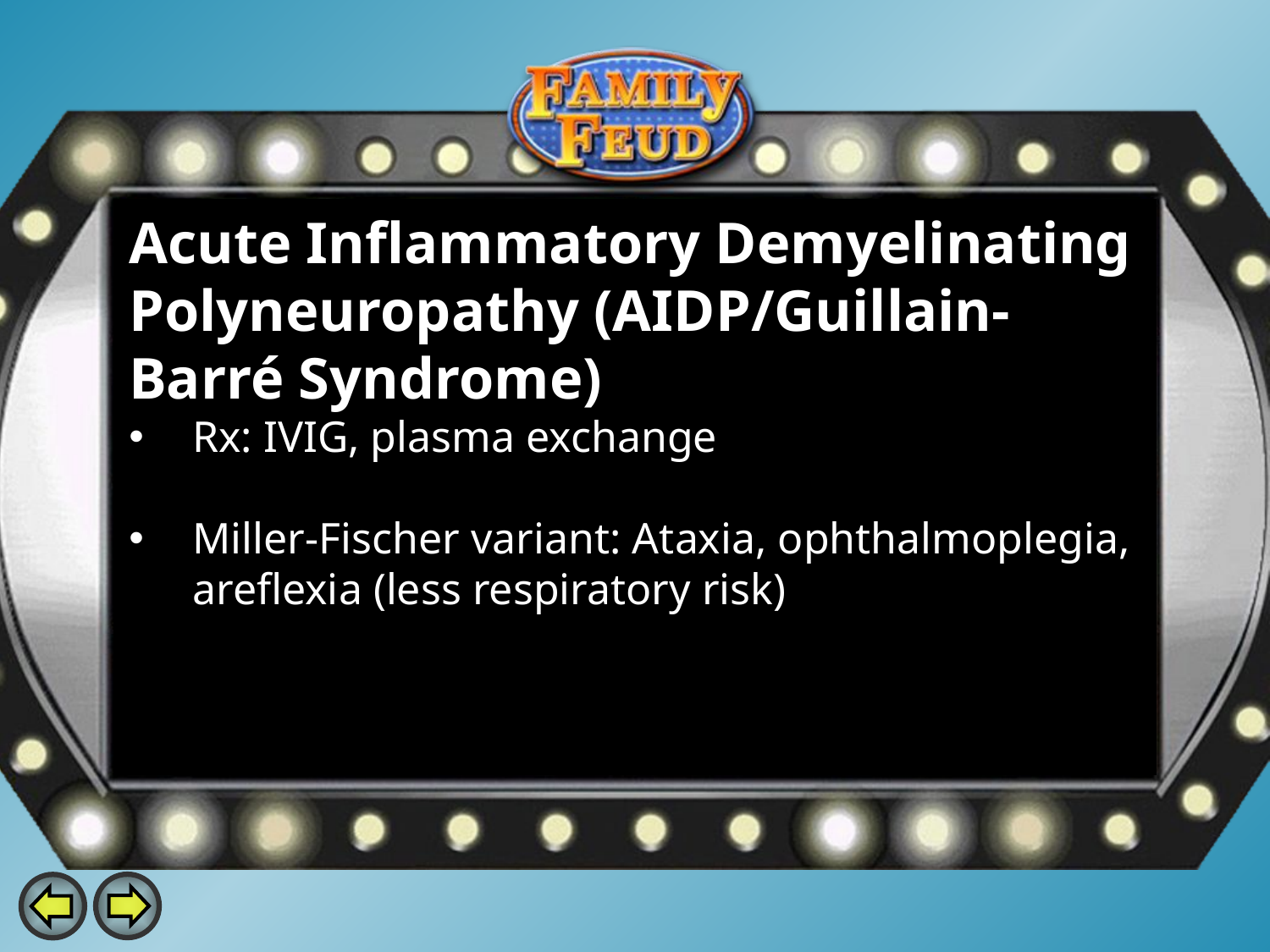

Acute Inflammatory Demyelinating Polyneuropathy (AIDP/Guillain-Barré Syndrome)
Rx: IVIG, plasma exchange
Miller-Fischer variant: Ataxia, ophthalmoplegia, areflexia (less respiratory risk)

## Slide 73
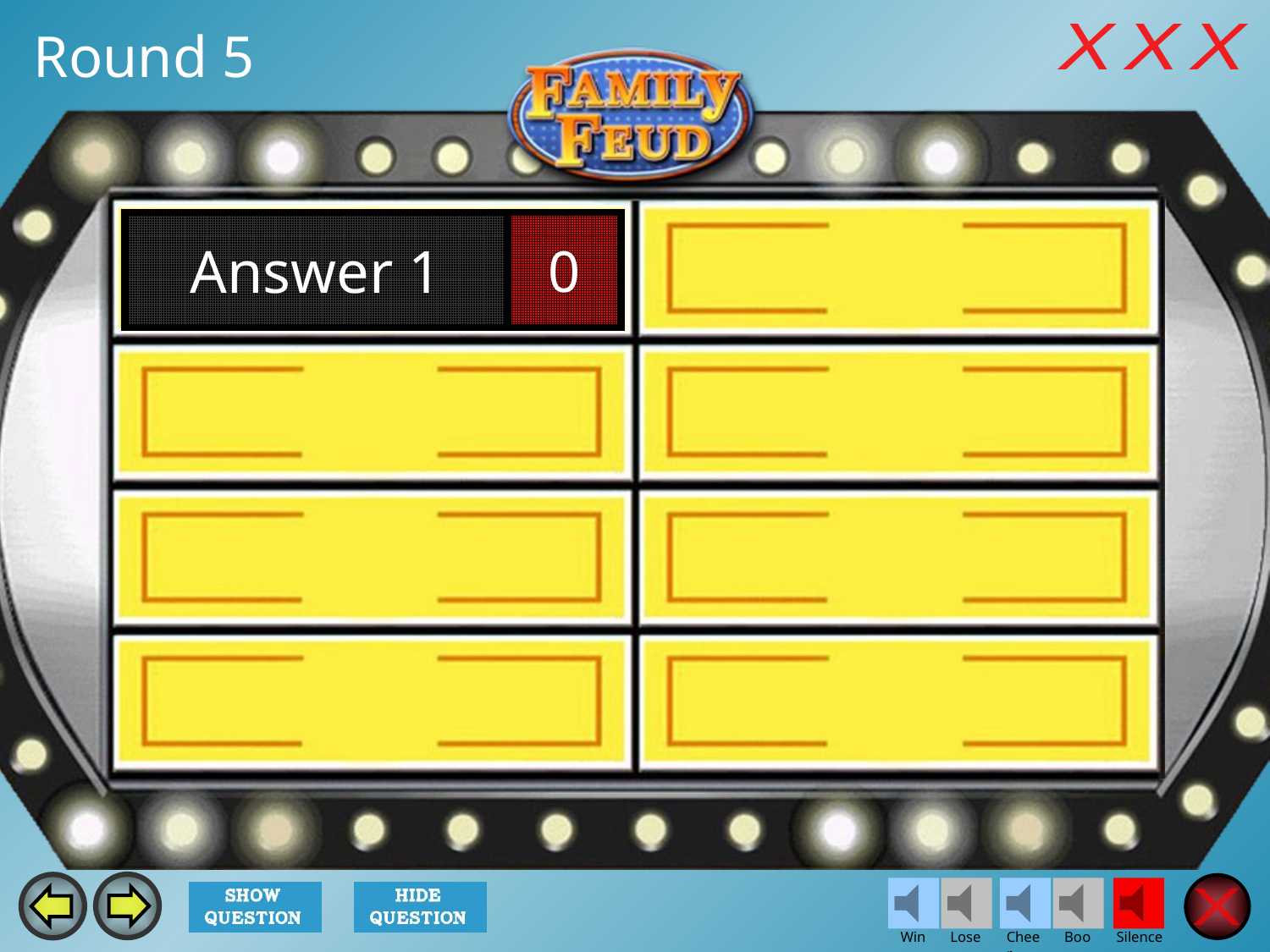

Viral encephalitis
X
X
X
X
X
X
Round 5
X
X
X
Answer 1
0
Win
Lose
Cheer
Boo
Silence
X
X
X

## Slide 74
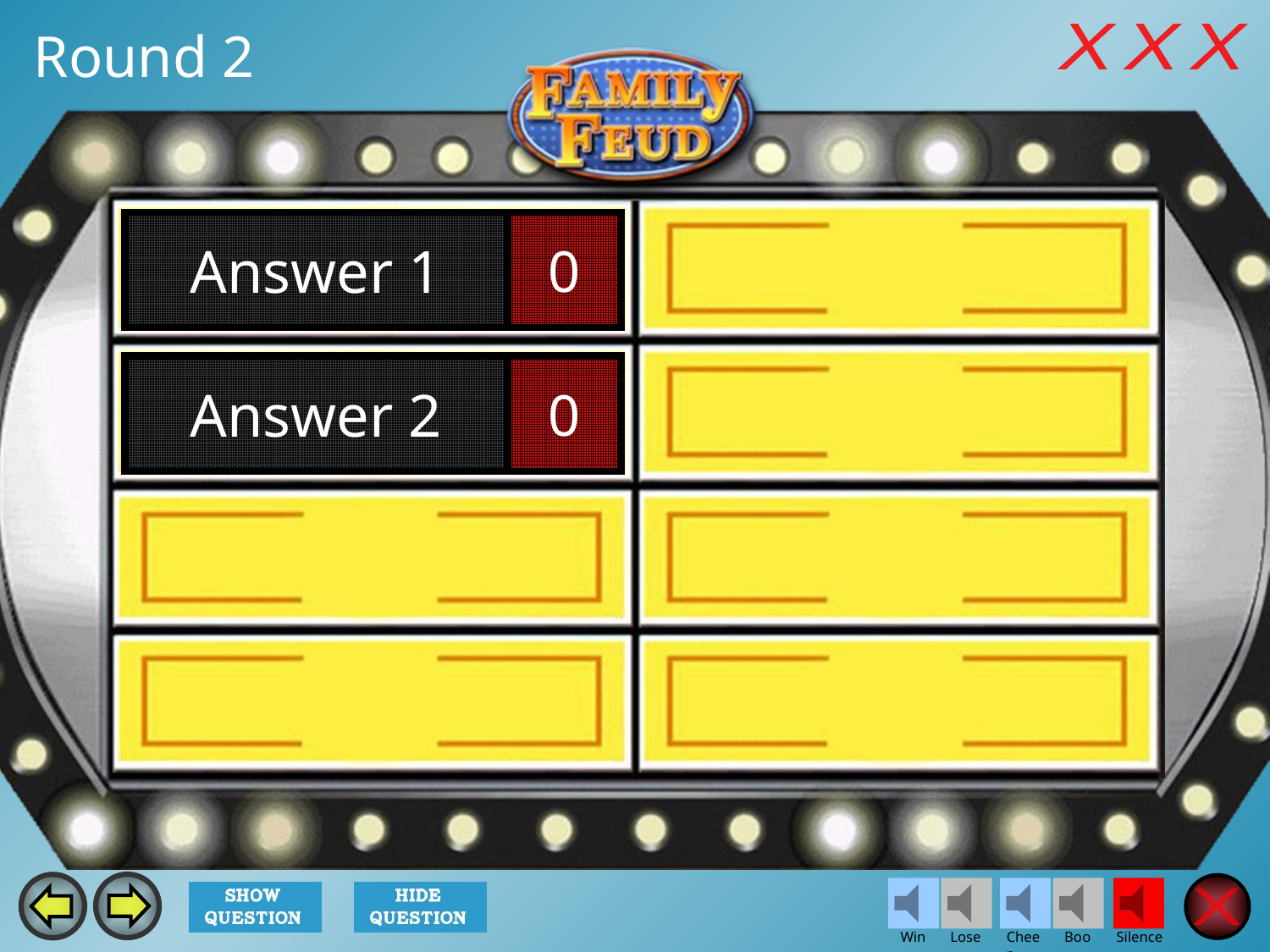

Question
X
X
X
X
X
X
Round 2
X
X
X
Answer 1
0
Answer 2
0
Win
Lose
Cheer
Boo
Silence
X
X
X

## Slide 75
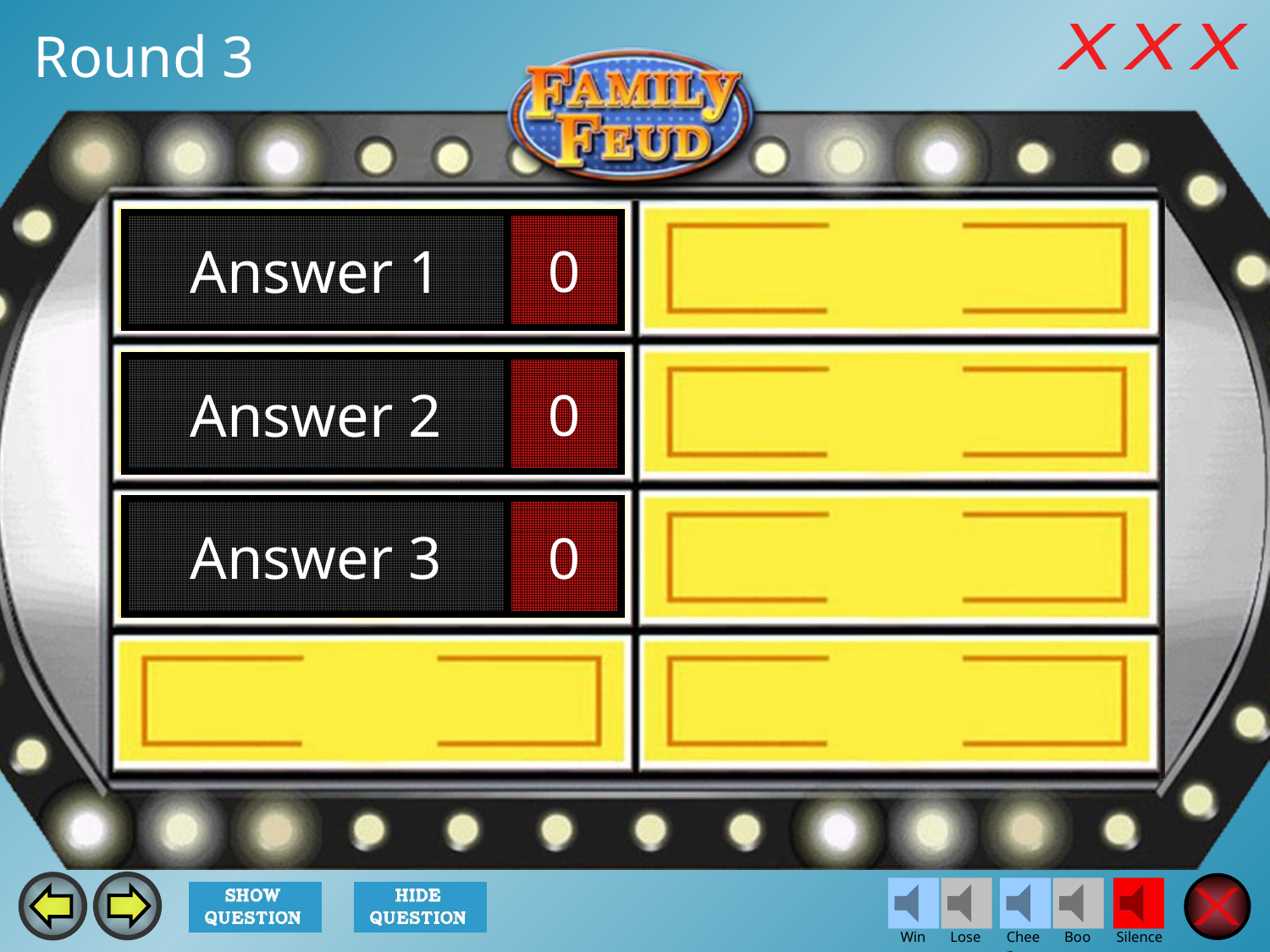

Question
X
X
X
X
X
X
Round 3
X
X
X
Answer 1
0
Answer 2
0
Answer 3
0
Win
Lose
Cheer
Boo
Silence
X
X
X

## Slide 76
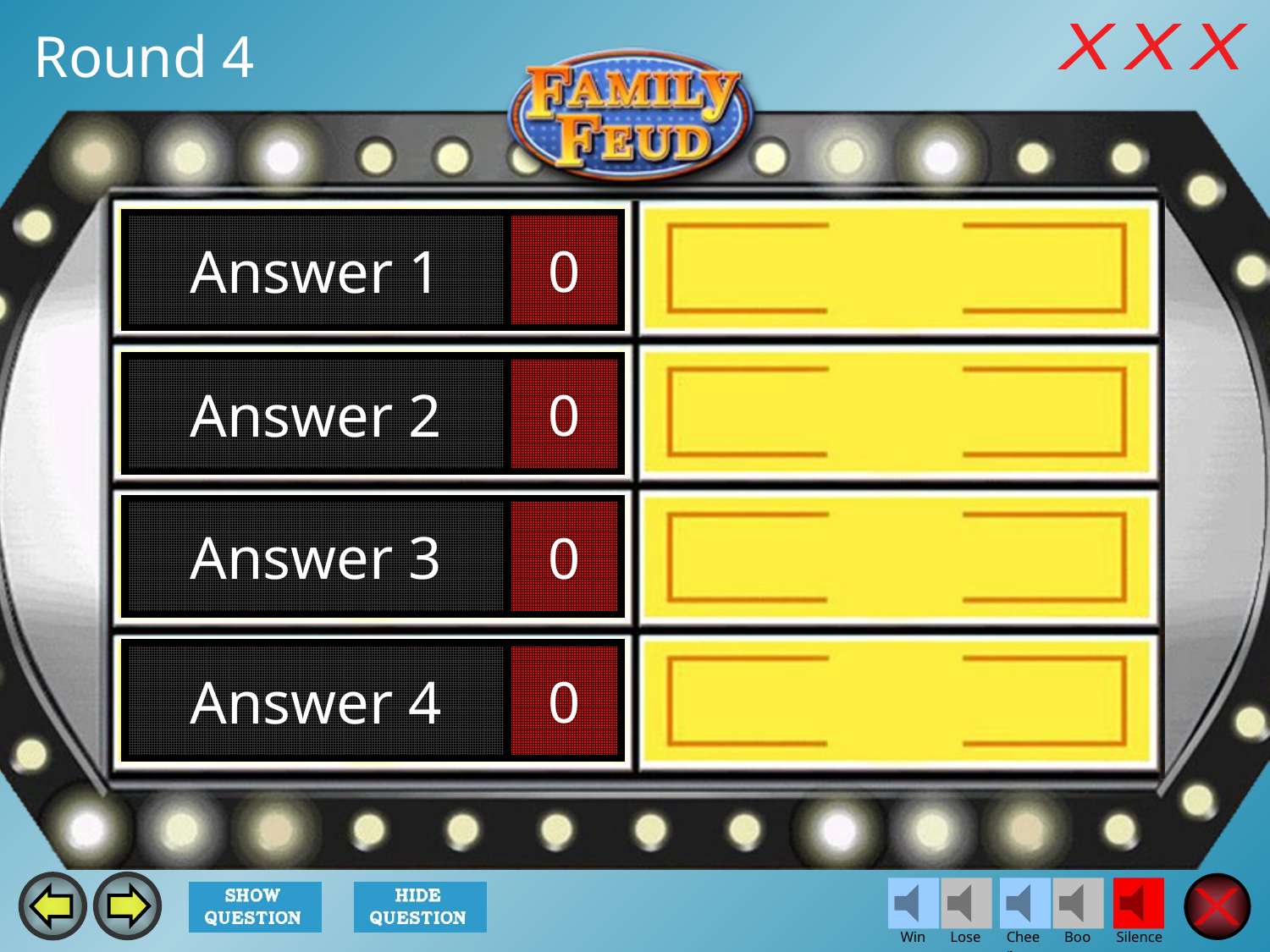

Question
X
X
X
X
X
X
Round 4
X
X
X
Answer 1
0
Answer 2
0
Answer 3
0
Answer 4
0
Win
Lose
Cheer
Boo
Silence
X
X
X

## Slide 77
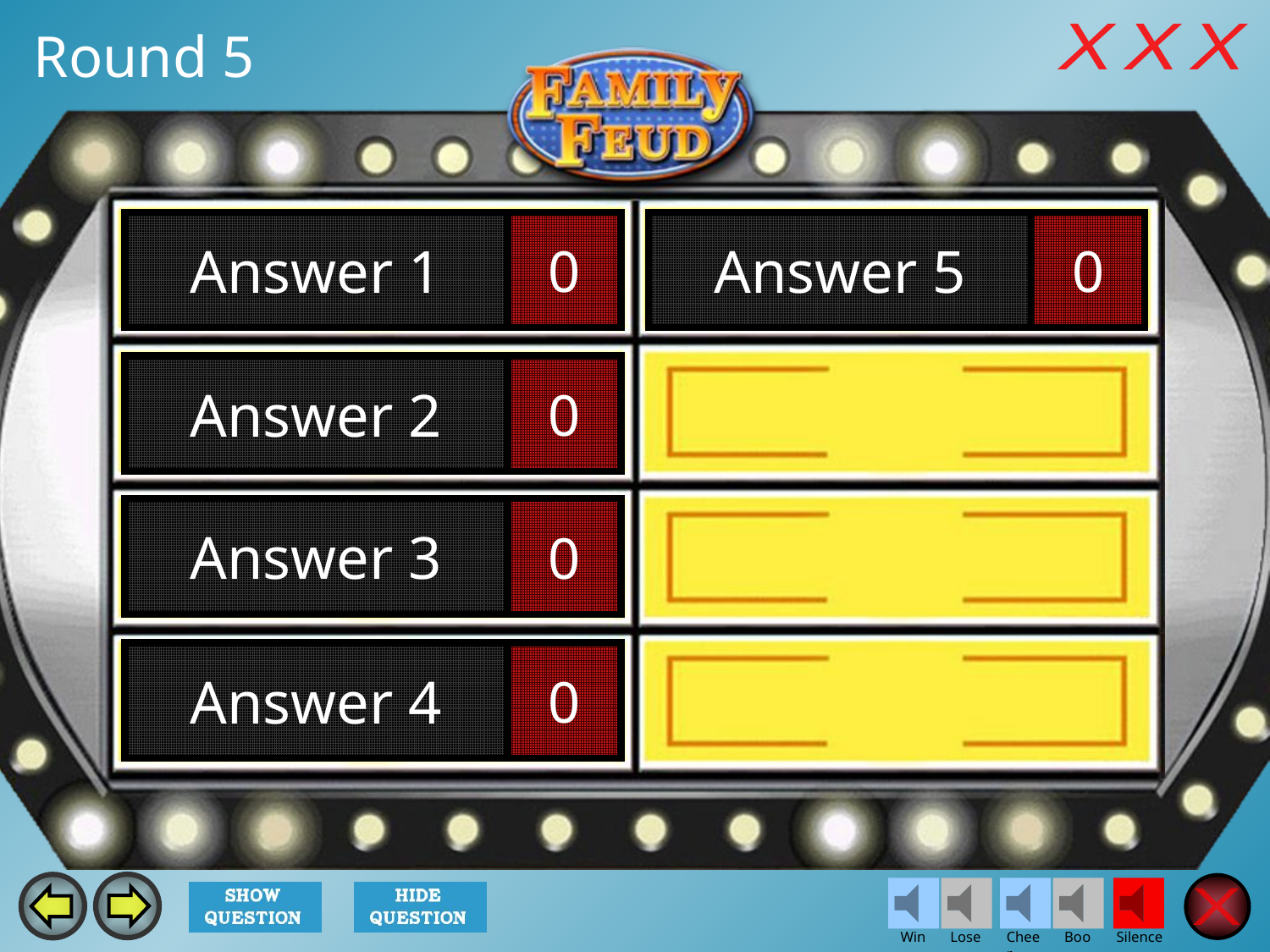

Question
X
X
X
X
X
X
Round 5
X
X
X
Answer 1
0
Answer 5
0
Answer 2
0
Answer 3
0
Answer 4
0
Win
Lose
Cheer
Boo
Silence
X
X
X

## Slide 78
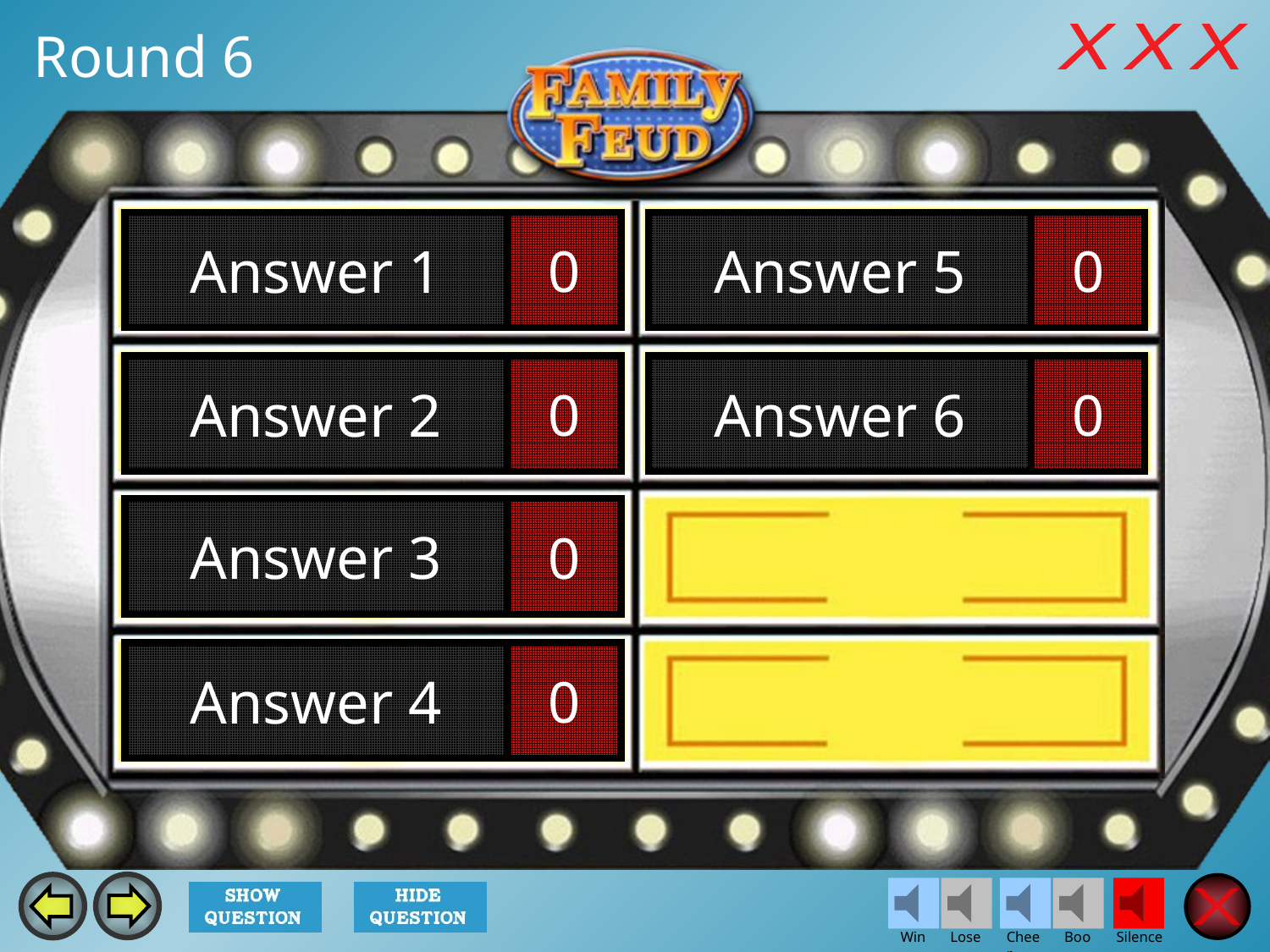

Question
X
X
X
X
X
X
Round 6
X
X
X
Answer 1
0
Answer 5
0
Answer 2
0
Answer 6
0
Answer 3
0
Answer 4
0
Win
Lose
Cheer
Boo
Silence
X
X
X

## Slide 79
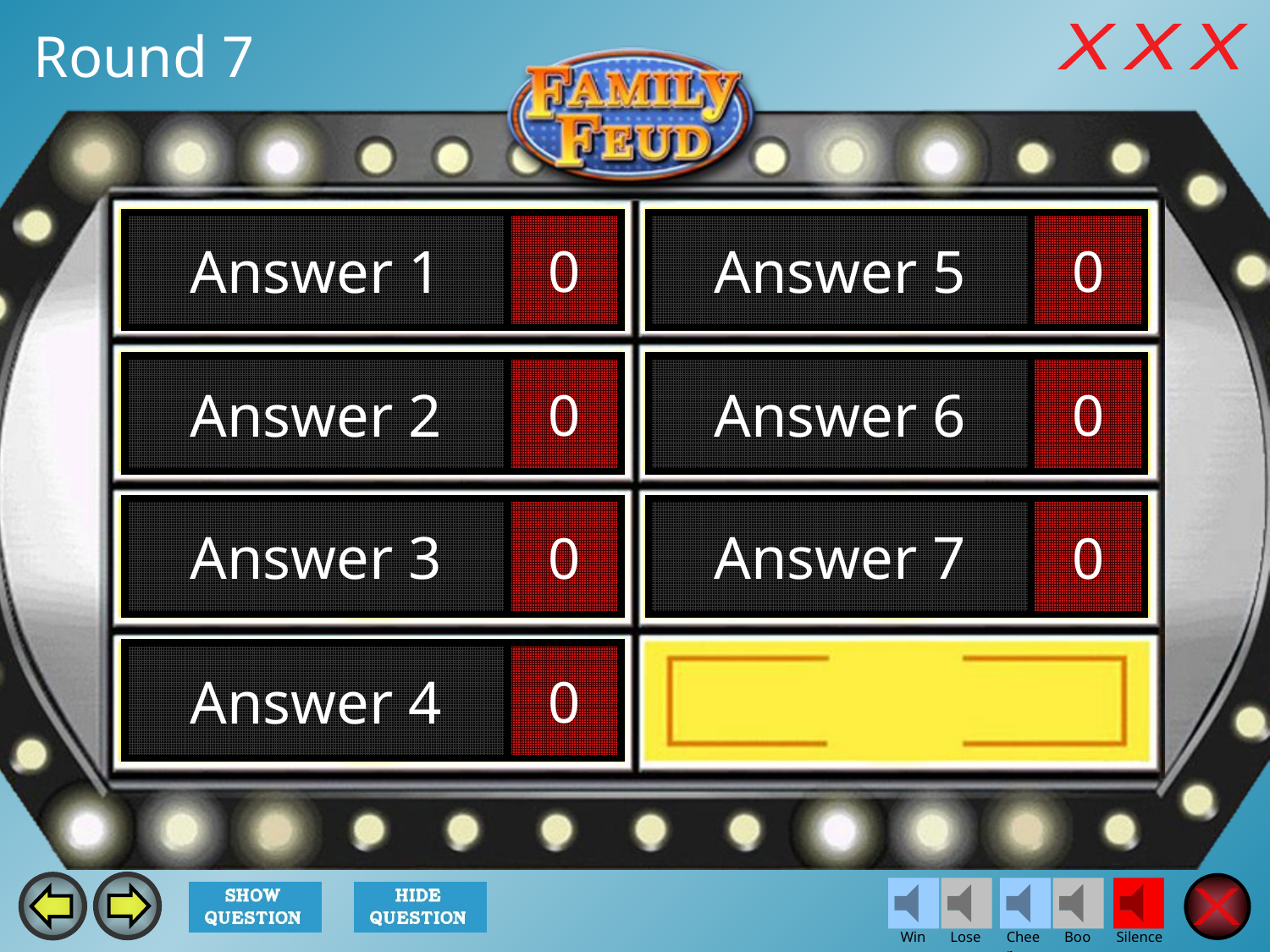

Question
X
X
X
X
X
X
Round 7
X
X
X
Answer 1
0
Answer 5
0
Answer 2
0
Answer 6
0
Answer 3
0
Answer 7
0
Answer 4
0
Win
Lose
Cheer
Boo
Silence
X
X
X

## Slide 80
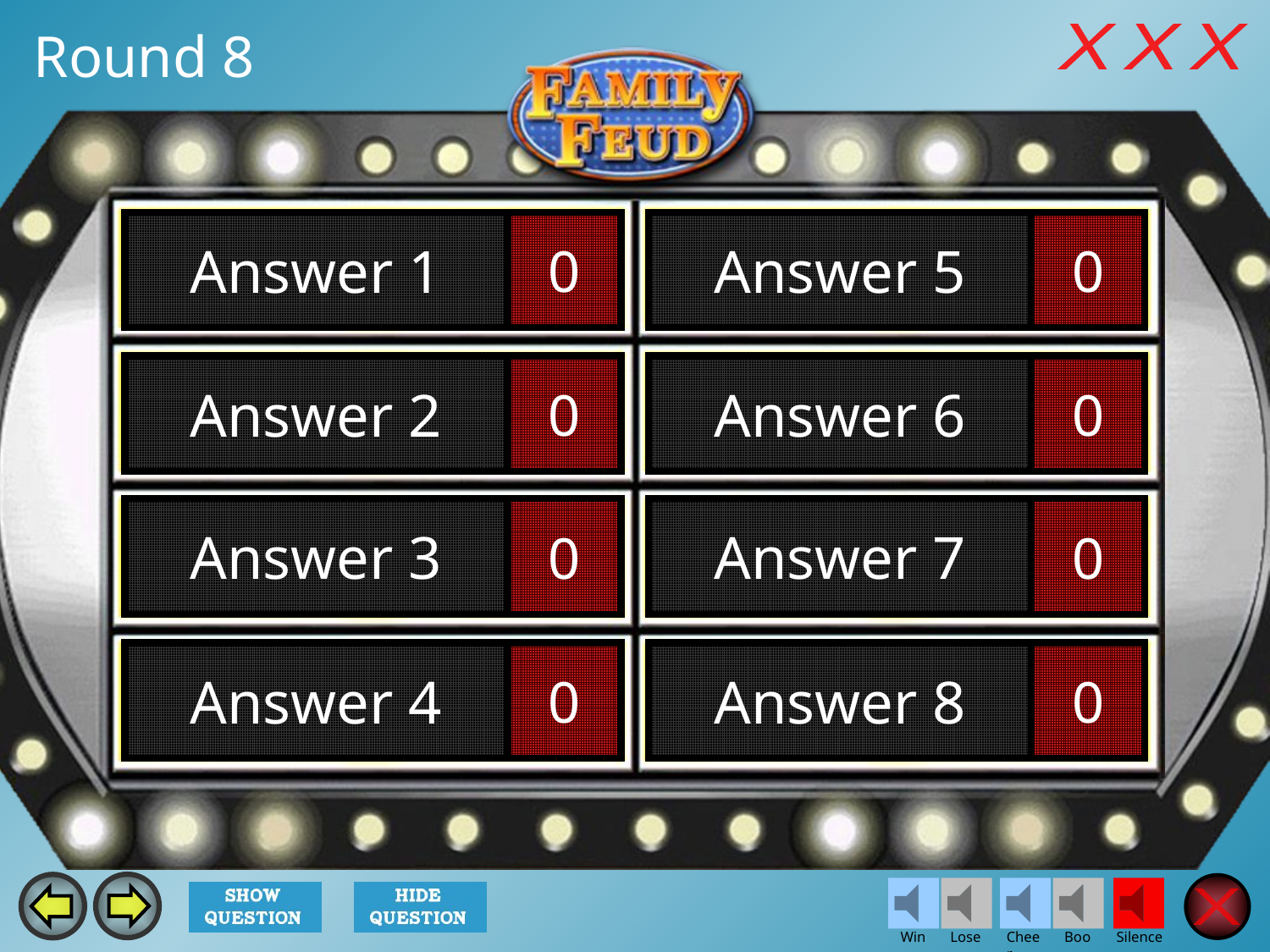

Question
X
X
X
X
X
X
Round 8
X
X
X
Answer 1
0
Answer 5
0
Answer 2
0
Answer 6
0
Answer 3
0
Answer 7
0
Answer 4
0
Answer 8
0
Win
Lose
Cheer
Boo
Silence
X
X
X
